# Asymmetric synthesis of allylic amines *via* hydroamination of allenes with benzophenone imine†
†Electronic supplementary information (ESI) available: Experimental procedures and detailed characterization data of all new compounds. See DOI: 10.1039/c5sc04984a


**DOI:** 10.1039/c5sc04984a

**Published:** 2016-02-09

**Authors:** Kun Xu, Yu-Hsuan Wang, Vahid Khakyzadeh, Bernhard Breit

**Affiliations:** a Institut für Organische , Chemie Albert-Ludwigs-Universität Freiburg , Albertstrasse 21 , 79104 Freiburg , Germany . Email: bernhard.breit@chemie.uni-freiburg.de ; Fax: +49-761-203-8715

## Abstract

Rhodium-catalyzed highly regio- and enantioselective hydroamination of allenes is reported. Exclusive branched selectivities and excellent enantioselectivities were achieved applying a rhodium(i)/Josiphos catalyst. This method permits the practical synthesis of valuable α-chiral allylic amines using benzophenone imine as ammonia carrier.

## Abstract 2

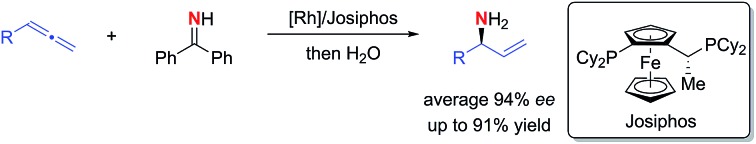
Rhodium-catalyzed highly regio- and enantioselective hydroamination of allenes is reported.

## 

α-Chiral amines are of wide interest in organic synthesis1*a* due to their broad application in pharmaceutical research,1b,c catalysis1d,e and natural product synthesis1*f* (Scheme 1). Among them, the synthesis of α-chiral allylic amines is particularly important because of the versatility of the allylic moiety for further structural elaboration.^2^ In the past few decades, significant efforts have advanced the efficiency towards their synthesis. Many elegant approaches including allylic substitution,^3^ Overman rearrangements,^4^ allylic C–H amination^5^ and imine vinylation^6^ have been reported. However, these methods need pre-installation of a leaving group or stoichiometric amounts of an oxidant/metal-containing reagent. In this regard, efficient synthetic methods for the synthesis of α-chiral allylic amines are highly desirable.

**Scheme 1:**
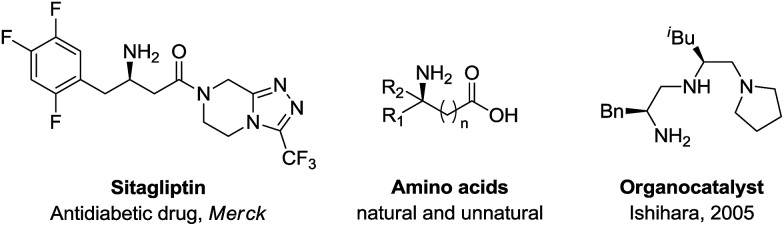
Applications of α-chiral amines.

The catalytic and enantioselective addition of simple ammonia (NH_3_) to allenes would represent one of the most atom-economic transformation towards the synthesis of α-chiral allylic amines.^7^–^9^ However, initial experiments revealed this transformation to be very challenging, presumably due to the following reasons: (1) the volatility and toxicity of gaseous NH_3_ makes it less interesting in terms of practicality; (2) the catalytic systems were inactive in the presence of ammonia, possibly due to the high basicity of sp^3^ hybridized nitrogen atom, and its difficulty to undergo oxidative addition to a transition metal center.^10^

To address these issues, we assumed that the easy-to-use and *N*-sp^2^-hybridized benzophenone imine^11^ could serve as an ammonia carrier: (1) benzophenone imine is commercially available and can be easily prepared *via* condensation of benzophenone with ammonia;^12^ (2) the sp^2^ hybridized imine nitrogen is more reactive towards allenes in the presence of a suitable transition metal catalyst; (3) the final α-chiral primary allylic amines can be obtained *via* simple hydrolysis, and the benzophenone can be recycled (Scheme 2).

**Scheme 2:**
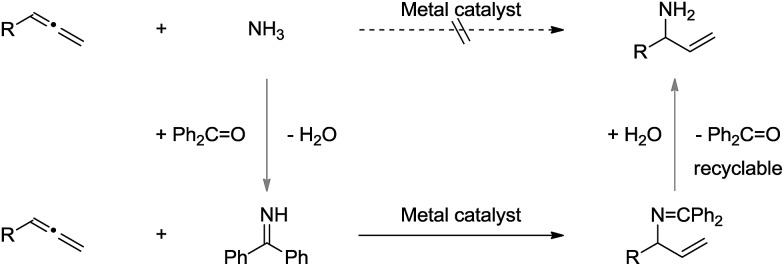
Proposed synthesis of α-chiral allylic amines using benzophenone imine as ammonia carrier.

The initial assessment was performed by coupling cyclohexylallene and benzophenone imine using [{Rh(COD)Cl}_2_] (2.5 mol%) and racemic ligand 1,4-bis(diphenylphosphino)butane (**L1**, 10 mol%) in 1,2-dichloroethane (DCE) at 80 °C (Table 1, entry 1). The reaction afforded the desired product with 10% NMR yield. We wondered whether addition of acid would facilitate the reaction by promoting the catalytic cycle. Indeed, both trifluoroacetic acid (TFA) and pyridinium *p*-toluenesulfonate (PPTS) could significantly improve the yield, while the addition of *p*-toluenesulfonic acid (PTSA) had no effect (Table 1, entry 2–4). These promising results encouraged us to test the feasibility of its asymmetric variant. The (*S*)-Segphos ligand (**L2**) only led to 10% of the desired product. The (*R*,*R*)-DIOP (**L3**) gave 76% of the isolated amide product **1a**, while only moderate ee was obtained. Further screening led to the discovery of Josiphos (**L4**), which afforded **1a** with 72% yield and 92% ee.^13^

**Table 1:** Reaction development

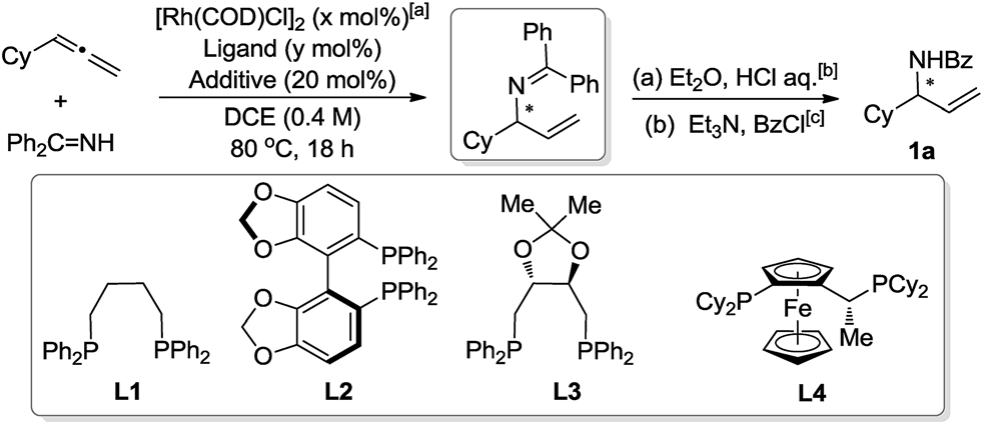					
Entry Entry	*x*	Ligand ( Ligand (*y*)	Additive Additive	Yield Yield^*d*^/%	ee ee^*e*^/%
1	2.5	**L1** (10)	—	(10)	—
2	2.5	**L1** (10)	PTSA	(11)	—
3	2.5	**L1** (10)	TFA	(50)	—
4	2.5	**L1** (10)	PPTS	(65)	—
5	2.0	**L1** (4.0)	PPTS	(59)	—
6	2.0	**L2** (4.0)	PPTS	(10)	—
7	2.0	**L3** (4.0)	PPTS	76^*f*^	54^*g*^
8	2.0	**L4** (4.0)	PPTS	72^*f*^	92

^*a*^Benzophenone imine (1.0 equiv.), cyclohexylallene (1.5 equiv.).

^*b*^Et_2_O (2.0 ml), HCl aq. (2.0 ml, 2.0 M, 4.0 mmol), room temperature, 24 hours.

^*c*^CH_2_Cl_2_ (2.0 ml), Et_3_N (223 μl, 1.6 mmol, 4.0 equiv.), benzoyl chloride (84.3 mg, 0.6 mmol, 1.5 equiv.).

^*d*^ ^1^H NMR yield of the coupling product (hydroamination step) in the crude reaction mixture using 1,3,5-trimethoxybenzene as internal standard.

^*e*^ee of **1a** was determined by chiral HPLC.

^*f*^Yield is that of the isolated product of **1a**.

^*g*^Acetonitrile was used as solvent.

With the optimized conditions in hand, we then investigated the feasibility of various allene substrates (Scheme 3, **1a–i**). Interestingly, in basically all cases perfect regioselectivities and excellent enantioselectivities were observed. Allenes containing alkyl substituents as well as ether, thio ether, phthalimide and sulfone functional groups were well tolerated.

**Scheme 3:**
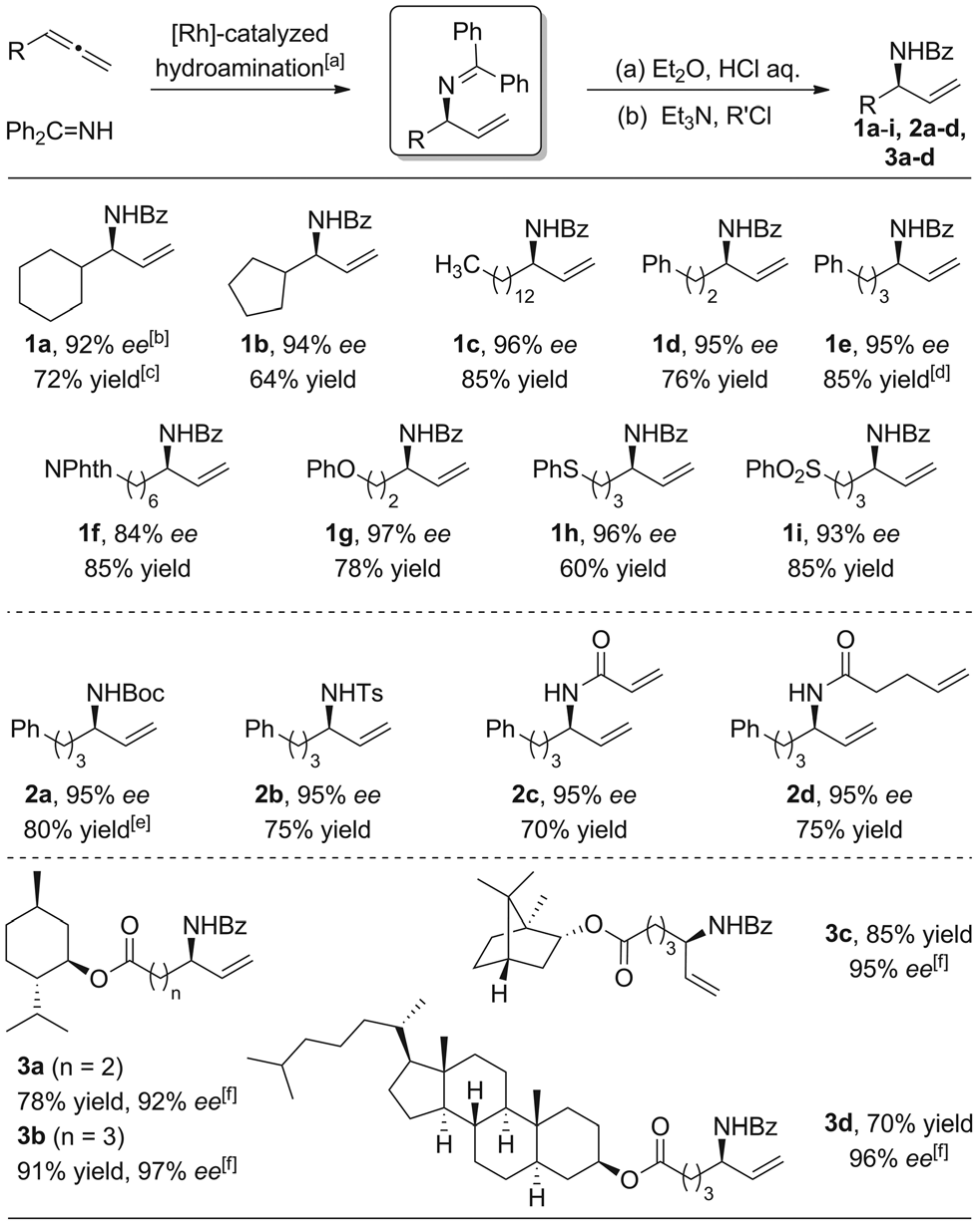
Scope of allenes and one-pot synthesis of allylic amides. [a] Benzophenone imine (1.0 equiv., 0.4 mmol), allene (1.5 equiv.), [{Rh(COD)Cl}_2_] (2.0 mol%), **L4** (4.0 mol%), PPTS (20 mol%), DCE (1.0 ml). [b] Determined by chiral HPLC. [c] Yield is that of the isolated product. [d] The reaction was performed in 0.2 mmol scale. [e] Boc_2_O was used in the protection step. Phth = phthaloyl; Ts = 4-methylbenzene-1-sulfonyl. [f] Determined by chiral HPLC after transesterification with ethanol.

**Scheme 4:**
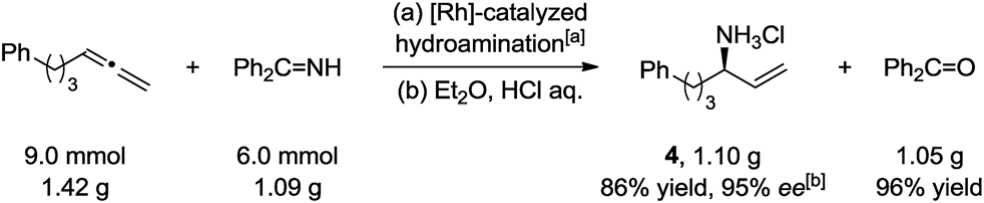
Large scale synthesis of primary allylic amine. [a] Scope conditions. [b] Determined by its amide derivative **1e***via* chiral HPLC.

Direct synthesis of branched allylic amides using simple amides and allenes was difficult. However, a one-pot synthesis of branched allylic amides or carbamate can be achieved easily upon acylation or sulfonylation of the crude allylic amine with the corresponding acyl/sulfonyl chlorides or anhydride, respectively (Scheme 3, **2a–d**).

Hydroamination with bioactive moieties containing substrates using the scope conditions resulted in the desired branched allylic amines with high yields and excellent enantioselectivities (Scheme 3, **3a–c**).

To test the practicality of this method for primary amine synthesis, chiral allylic amine HCl salt **4** was synthesized under scope conditions in a 1.1 gram scale with 86% yield and 95% ee. The released ammonia carrier (benzophenone) can be recycled after hydrolysis with 96% yield (Scheme 4).

**Scheme 5:**
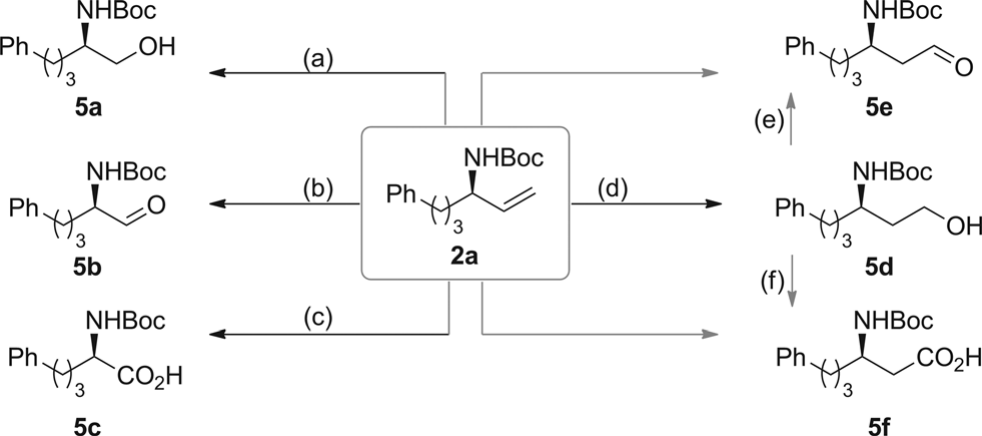
Synthetic transformations of chiral allylic amines. (a) O_3_, CH_2_Cl_2_, –78 °C; then NaBH_4_, CH_3_OH, 0 °C to r.t.; 90% yield, 95% ee (**5a**). (b): O_3_, CH_2_Cl_2_, –78 °C; then PPh_3_, –78 °C to r.t.; 85% yield, 95% ee (**5b**). (c): RuCl_3_, NaIO_4_, MeCN/CCl_4_/H_2_O (1/1/1.5), r.t.; 82% yield, 95% ee (**5c**). (d) 9-BBN, THF, –78 °C to r.t.; then EtOH, NaOH, H_2_O_2_ (30%), –10 °C to r.t.; 95% yield, 95% ee (**5d**). (e): C_5_H_5_NSO_3_, Et_3_N, DMSO, 0 °C to r.t.; 88% yield, 95% ee (**5e**) (f) PhI(OAc)_2_, TEMPO, CH_3_CN/H_2_O (1/1), r.t.; 48% yield, 95% ee (**5f**).

To exemplify the utility of chiral allylic amines, derivatization of compound **2a** was performed (Scheme 5). Ozonolysis of **2a** followed by treatment with triphenylphosphine and NaBH_4_ gave β-amino alcohol **5a** and α-amino aldehyde **5b**, respectively. Direct oxidation of **2a** could afford α-amino acid **5c**. Hydroboration of **2a** using 9-borabicyclo(3.3.1)nonane (9-BBN), then oxidation with H_2_O_2_ led to the formation of γ-amino alcohol **5d**, which could be further oxidized to the corresponding β-amino aldehyde **5e** and β-amino acid **5f**.

Isotopic labeling experiments using deuterated benzophenone imine (Ph_2_C = ND) and deuterated PPTS (D-PPTS) were conducted under scope conditions.^13^ Deuterium incorporation was only observed at the internal position of the allylic double bond. Hence, we suggest that the mechanism follows a similar pathway as for previously reported coupling reactions.9*e* Oxidative addition of the benzophenone imine N–H bond to Rh(i) generates Rh(iii) complex. Hydrometalation of the less substituted double bond could generate σ-allyl-Rh complex, which is in equilibrium with the π-allyl-Rh complex. Reductive elimination of the allyl-Rh complexes generates the branched *N*-allylic amine.

To conclude, we have developed the first highly regio- and enantioselective hydroamination of allenes using benzophenone imine as an ammonia carrier *via* a rhodium/Josiphos catalyst system. The reaction gave valuable α-chiral primary allylic amines and α-chiral allylic amides in a practical manner. Recycle of the ammonia carrier with high yield maximized the atom-economy of this protocol. Applications of this method in target oriented synthesis and using more challenging terminal alkyne as the coupling partner for the enantioselective synthesis of branched allylic amines are currently under way in our laboratories and will be reported in due course.

## Supplementary Material

Supplementary information
