# Supplementary material for: Asymmetric synthesis of allylic amines via hydroamination of allenes with benzophenone imine
Source: Chem Sci. 2016 Feb 9;7(5):3313–6. doi: 10.1039/c5sc04984a (PMC6006468; doi:10.1039/c5sc04984a)

Electronic Supplementary Material (ESI) for Chemical Science.
This journal is © The Royal Society of Chemistry 2016

Supporting Information

Asymmetric Synthesis of Allylic Amines via Hydroamination of Allenes with Benzophenone Imine

Kun Xu, Yu-Hsuan Wang, Vahid Khakyzadeh and Bernhard Breit*

Institut für Organische Chemie, Albert-Ludwigs-Universität Freiburg, Albertstrasse 21, 79104 Freiburg, Germany

Table of Contents

General and Materials S2

General Procedure for the Hydroamination of Allenes with Benzophenone Imine S3

Synthesis and Characterization of Allylic Amides S3

One-pot Synthesis of Allylic Amides S4

Hydroamination of Bioactive Moieties Containing Substrates S5

Large Scale Synthesis of Primary Allylic Amines S6

Derivatization of Allylic Amides S7

Mechanistic Investigations S8

References S9

NMR spectra S10

HPLC data S11

General

FCC (Flash Column Chromatography) was accomplished using MACHEREY-NAGEL silica gel 60® (230-400 mesh). **TLC** (Thin Layer Chromatography) was performed on aluminum plates pre-coated with silica gel (MERCK, $60F_{254}$), which were visualized by UV fluorescence (λ_{max} = 254 nm) and/or by staining with 1% w/v $KMnO_4$ in 0.5 M aqueous K_2CO_3. **NMR** (Nuclear Magnetic Resonance) spectra were acquired on a BRUKER Avance spectrometer (300, 400, or 500 MHz and 100.6, 126 MHz for ^{1}H and ^{13}C respectively). All ^{1}H NMR spectra are reported in parts per million (ppm) downfield of TMS and were measured relative to the signals at 7.26 ppm ($CHCl_3$) or 7.16 ppm (C_6D_6). All ^{13}C NMR spectra were reported in ppm relative to residual $CHCl_3$ (77.16 ppm) and were obtained with ^{1}H-decoupling. Data for ^{1}H NMR are described as following: chemical shift (δ in ppm), multiplicity (s, singlet; d, doublet; t, triplet; q, quartet; quin, quintet; sx, sextet; m, multiplet; app, apparent; br, broad signal), coupling constant (Hz), integration. Data for ^{13}C NMR spectra are described in terms of chemical shift (δ in ppm). **HRMS** (High resolution mass spectra) were obtained on a FINNIGAN MAT 8200 instrument (CI/NH_3: 110 eV; EI: 70 eV). **Chiral HPLC** was performed on a MERCK HITACHI HPLC apparatus (pump: L-7100, UV detector: D-7400, oven: L-7360; columns: AD-H, AD-3, OD-3, OJ-H, L-C2, L-C3, AD-3R, OD-3R, OJ-R, and OJ-3R 15-25 cm 4.6 cm, DAICEL). The **Optical Rotation** of chiral compounds was determined on a PERKIN-ELMER PE 241 apparatus and transformed for a given temperature according to the following formula:

$$[\boldsymbol{\alpha}]_{\boldsymbol{D}}^{\boldsymbol{T}} = \frac{\alpha \cdot 100}{c \cdot d}$$

α: measured value for optical rotation; *c*: concentration in g/100 ml; *d*: length of the cuvette in dm; T: temperature in °C.

Materials

Solvents: 1,2-Dichloroethane (DCE) was freshly distilled over CaH_2 and degassed by three Freeze-Pump-Thaw cycles prior to use. Solvents employed for work-up and column chromatography were purchased in technical grade quality and distilled by rotary evaporator before use.

Substrates: Benzophenone imine and allenes, if commercially available, were purchased from Sigma-Aldrich, ABCR, Alfa Aesar and used without further purification. Non-commercially available allenes were synthesized in our group according to literatures.[1]

Ligands and catalysts: The ligands and $[Rh(COD)Cl]_2$ were purchased from Sigma-Aldrich, ABCR, Alfa Aesar and used without further purification.

Deuterated samples: Ph_2C=ND was prepared by reaction of CD_3OD with Ph_2C=NLi, which was generated by reaction of n-BuLi with 1 equivalent of Ph_2C=NH in THF at -78 °C.[2] Deuterated pyridinium p-toluenesulfonate (D-PPTS) was prepared via deuterium exchange with CD_3OD for five times.

General procedure for the synthesis of allylic amides

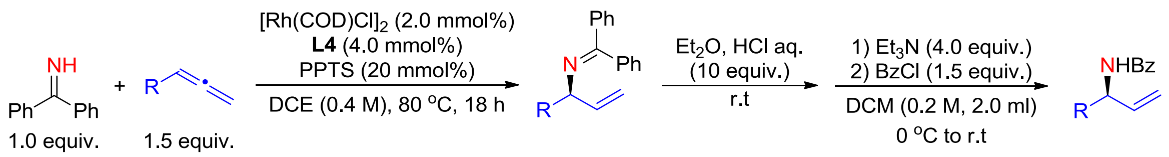

Step 1, **Hydroamination**: To a Schlenk tube was added $[Rh(COD)Cl]_2$ (3.94 mg, 0.008 mmol, 2 mol%), Ligand **L4** (9.70 mg, 0.016 mmol, 4 mol%), PPTS (20.10 mg, 0.08 mmol, 20%), benzophenone imine (72.5 mg, 0.4 mmol, 1.0 equiv), DCE (1.0 ml, 0.4 M) and allene (0.6 mmol, 1.5 equiv). The Schlenk tube was sealed and the mixture was stirred for 18 h at 80 °C. After cooling to room temperature, the solvent was removed under vacuum.

Step 2, **Hydrolysis**: To the hydroamination reaction mixture of *step 1* was added Et_2O (2.0 ml) and HCl aq. (2.0 ml, 2.0 M, 4 mmol) sequentially. The reaction was stirred at r.t for 24 hours. The volatiles were removed under vacuum.

Step 3, **Benzoyl protection**: To the resulting allylic amine HCl salt curde mixture of *step 2* was added CH_2Cl_2 (2.0 ml) and Et_3N (223 µl, 161.9 mg, 1.6 mmol, 4.0 equiv) and benzoyl chloride (84.3 mg, 0.6 mmol, 1.5 equiv) sequentially. The reaction mixture was stirred for 3 hours. The volatiles were removed and the residue was purified by FCC to obtain benzenphenone ketone and desired allylic amides.

Synthesis and characterization of allylic amides (1a-j)

1 (*S*)-*N*-(1-cyclohexylallyl)benzamide **(1a)**

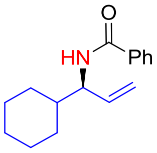

The reaction was performed with cyclohexylallene (87 µl, 73.3 mg, 0.6 mmol). The crude product was purified by FCC on silica gel (EA/CH = 1/4, R_f = 0.34) to afford the product as a white solid (70.0 mg, 72 %).

m.p.: 119 – 120 °C; **^{1}H NMR** (400MHz, $CDCl_3$) δ = 7.81 - 7.76 (m, 2 H), 7.52 - 7.41 (m, 3 H), 6.12 - 6.00 (m, 1 H), 5.85 (ddd, *J* = 6.1, 10.4, 17.2 Hz, 1 H), 5.25 - 5.15 (m, 2 H), 4.60 - 4.52 (m, 1 H), 1.84 - 1.73 (m, 4 H), 1.72 - 1.63 (m, 1 H), 1.63 - 1.52 (m, 1 H), 1.32 - 1.00 (m, 5 H); **^{13}C NMR** (100.6MHz, $CDCl_3$) δ = 166.9, 137.0, 135.1, 131.5, 128.7, 126.9, 115.9, 56.6, 42.4, 29.6, 29.0, 26.5, 26.24, 26.21; **HRMS-ESI** (MeOH, m/z): $[M+H]^+$ calcd for $C_{16}H_{22}NO$, 244.17014; found, 244.17030; **HPLC** (CHIRALCEL® AD-H, *n*-heptane / ^{i}PrOH = 95:5, 1 mL/min) t_R = 7.22 min (minor), t_R = 8.34 min (major), 92% *ee* (***S***); $[\alpha]_D^{25}$ = - 36.00 (c = 0.383, $CHCl_3$).

Recovery of benzophenone via FCC (EA/CH = 1/4, R_f = 0.65, Eluent: EA/CH = 1/15): 71.0 mg, 97%, analytical data is identical with literature.[3]

2 (*S*)-*N*-(1-cyclopentylallyl)benzamide **(1b)**

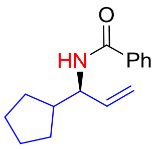

The reaction was performed with propa-1,2-dien-1-ylcyclopentane (64.9 mg, 0.6 mmol). The crude product was purified by FCC on silica gel (EA/CH = 1/4, R_f = 0.33) to afford the product as a white solid (59.0 mg, 64 %).

m.p.: 86 – 87 °C; **^{1}H NMR** (400MHz, $CDCl_3$) δ = 7.80 - 7.75 (m, 2 H), 7.50 - 7.45 (m, 1 H), 7.44 - 7.38 (m, 2 H), 6.19 (d, *J* = 6.9 Hz, 1 H), 5.86 (ddd, *J* = 5.9, 10.4, 17.1 Hz, 1 H), 5.25 - 5.18 (m, 1 H), 5.13 (td, *J* = 1.4, 10.4 Hz, 1 H), 4.60 - 4.51 (m, 1 H), 2.08 (sxt, *J* = 8.2 Hz, 1 H), 1.82 - 1.70 (m, 2 H), 1.70 - 1.48 (m, 4 H), 1.44 - 1.28 (m, 2 H); **^{13}C NMR** (100.6MHz, $CDCl_3$) δ = 166.9, 137.8, 135.0, 131.4, 128.6, 127.0, 115.3, 56.0, 44.5, 29.5, 29.4, 25.6, 25.4; **HRMS-APCI** (MeOH, m/z): $[M+H]^+$ calcd for $C_{15}H_{20}NO$, 230.15394; found, 230.15404; **HPLC** (CHIRALCEL® AD-H, *n*-heptane / ^{i}PrOH = 98:2, 1 mL/min) t_R = 39.90 min (minor), t_R = 47.15 min (major), 94% *ee* (***S***); $[\alpha]_D^{25}$ = - 35.80 (c = 0.392, $CHCl_3$).

Determination of absolute configuration

Absolute configuration was determined by comparing the specific rotation of **1b** with literature.[4] Absolute configurations for other allylation products were assignied by analogy.

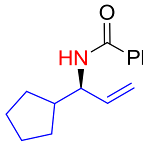

(*S*)-*N*-(1-cyclopentylallyl)benzamide

Observed Specific Rotation:
$[\alpha]_D^{25}$ = - 35.80 (c = 0.392, $CHCl_3$), 94% *ee*

Literature value [(*S*)-enantiomer]:
$[\alpha]_D^{22}$ = - 16.7 (c = 1, $CHCl_3$), 82% *ee*

3 (*R*)-*N*-(hexadec-1-en-3-yl)benzamide **(1c)**

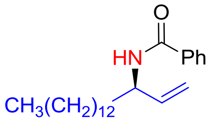

The reaction was performed with hexadeca-1,2-diene (133.4 mg, 0.6 mmol). The crude product was purified by FCC on silica gel (EA/CH = 1/4, R_f = 0.33) to afford the product as a white solid (117.0 mg, 85 %).

m.p.: 85 – 86 °C; **^{1}H NMR** (400MHz, $CDCl_3$) δ = 7.81 - 7.75 (m, 2 H), 7.53 - 7.48 (m, 1 H), 7.47 - 7.41 (m, 2 H), 6.01 - 5.92 (m, 1 H), 5.86 (ddd, *J* = 5.6, 10.4, 17.2 Hz, 1 H), 5.23 (td, *J* = 1.4, 17.2 Hz, 1 H), 5.15 (td, *J* = 1.4, 10.4 Hz, 1 H), 4.73 - 4.61 (m, 1 H), 1.72 - 1.55 (m, 2 H), 1.44 - 1.20 (m, 22 H), 0.91 - 0.85 (m, 3 H); **^{13}C NMR** (100.6MHz, $CDCl_3$) δ = 166.9, 138.6, 135.0, 131.5, 128.7, 127.0, 115.0, 51.9, 35.2, 32.0, 29.81, 29.78, 29.77, 29.76, 29.70, 29.65, 29.6, 29.5, 25.9, 22.8, 14.2; **HRMS-APCI** (MeOH, m/z): $[M+H]^+$ calcd for $C_{23}H_{38}NO$, 344.2948; found, 344.2950; **HPLC** (CHIRALCEL® OD3, *n*-heptane / ^{i}PrOH = 95:5, 1 mL/min) t_R = 5.66 min (major), t_R = 7.51 min (minor), 96% *ee* (***R***); $[\alpha]_D^{25}$ = - 19.30 (c = 0.351, $CHCl_3$).

4 (*R*)-*N*-(5-phenylpent-1-en-3-yl)benzamide **(1d)**

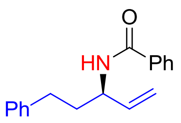

The reaction was performed with penta-3,4-dien-1-ylbenzene (86.5 mg, 0.6 mmol). The crude product was purified by FCC on silica gel (EA/CH = 1/4, R_f = 0.32) to afford the product as a white solid (84.9 mg, 80 %).

m.p.: 71 – 72 °C; **^{1}H NMR** (400MHz, $CDCl_3$) δ = 7.74 - 7.68 (m, 2 H), 7.51 - 7.46 (m, 1 H), 7.43 - 7.38 (m, 2 H), 7.31 - 7.27 (m, 2 H), 7.23 - 7.17 (m, 3 H), 6.23 (d, J = 8.5 Hz, 1 H), 5.90 (ddd, J = 5.6, 10.4, 17.2 Hz, 1 H), 5.25 (td, J = 1.4, 17.2 Hz, 1 H), 5.19 (td, J = 1.4, 10.4 Hz, 1 H), 4.80 - 4.71 (m, 1 H), 2.74 (t, J = 7.9 Hz, 2 H), 2.06 - 1.94 (m, 2 H); **^{13}C NMR** (100.6MHz, $CDCl_3$) δ = 166.8, 141.6, 138.1, 134.6, 131.4, 128.6, 128.5, 128.4, 126.9, 126.0, 115.4, 51.7, 36.4, 32.2; **HRMS-ESI** (MeOH, m/z): $[M+Na]^+$ calcd for $C_{18}H_{19}NONa$, 288.13589; found, 288.13606; **HPLC** (CHIRALCEL® L-C2, *n*-heptane / ^{i}PrOH = 95:5, 1 mL/min) t_R = 32.89 min (major), t_R = 52.67 min (minor), 95% *ee* (***R***); $[\alpha]_D^{25}$ = - 26.70 (c = 0.570, $CHCl_3$).

5 (*R*)-*N*-(6-phenylhex-1-en-3-yl)benzamide **(1e)**

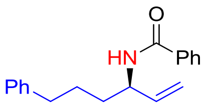

The reaction was performed in a 0.2 mmol scale, all the materials used were halved. Hexa-4,5-dien-1-ylbenzene (47.5 mg, 0.3 mmol). The crude product was purified by FCC on silica gel (EA/CH = 1/4, R_f = 0.31) to afford the product as a white solid (47.5 mg, 85 %).

m.p.: 110 - 111 °C; **^{1}H NMR** (400MHz, $CDCl_3$) δ = 7.79 - 7.73 (m, 2 H), 7.53 - 7.47 (m, 1 H), 7.46 - 7.40 (m, 2 H), 7.30 - 7.25 (m, 2 H), 7.21 - 7.15 (m, 3 H), 5.96 (d, J = 8.3 Hz, 1 H), 5.84 (ddd, J = 5.7, 10.4, 17.2 Hz, 1 H), 5.26 - 5.19 (m, 1 H), 5.15 (td, J = 1.4, 10.4 Hz, 1 H), 4.77 - 4.68 (m, 1 H), 2.73 - 2.60 (m, 2 H), 1.81 - 1.61 (m, 4 H); **^{13}C NMR** (100.6MHz, $CDCl_3$) δ = 166.9, 142.1, 138.4, 134.8, 131.5, 128.7, 128.5, 128.4, 126.9, 125.9, 115.3, 51.7, 35.7, 34.5, 27.7; **HRMS-APCI** (MeOH, m/z): $[M+H]^+$ calcd for $C_{19}H_{22}NO$, 280.17014; found, 280.17020; **HPLC** (CHIRALCEL® L-C2, *n*-heptane / ^{i}PrOH = 98:2, 1 mL/min) t_R = 37.28 min (major), t_R = 45.39 min (minor), 95% *ee* (***R***); $[\alpha]_D^{25}$ = - 14.50 (c = 0.400, $CHCl_3$).

6 (*R*)-*N*-(9-(1,3-dioxoisoindolin-2-yl)non-1-en-3-yl)benzamide **(1f)**

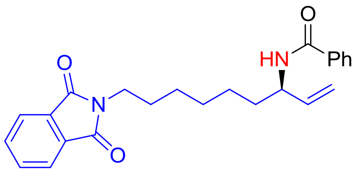

The reaction was performed with 2-(nona-7,8-dien-1-yl)isoindoline-1,3-dione (161.6 mg, 0.6 mmol). The crude product was purified by FCC on silica gel (EA/CH = 1/2, R_f = 0.30) to afford the product as a white solid (133.0 mg, 85 %).

m.p.: 119 – 120 °C; **^{1}H NMR** (400MHz, $CDCl_3$) δ = 7.86 - 7.75 (m, 4 H), 7.73 - 7.66 (m, 2 H), 7.51 - 7.34 (m, 3 H), 6.01 (d, *J* = 7.6 Hz, 1 H), 5.90 - 5.79 (m, 1 H), 5.22 (dd, *J* = 0.9, 17.2 Hz, 1 H), 5.14 (dd, *J* = 0.9, 10.5 Hz, 1 H), 4.66 (quin, *J* = 6.8 Hz, 1 H), 3.67 (t, *J* = 7.3 Hz, 2 H), 1.73 - 1.53 (m, 4 H), 1.47 - 1.28 (m, 6 H); **^{13}C NMR** (100.6MHz, $CDCl_3$) δ = 168.5, 166.9, 138.5, 134.9, 133.9, 132.3, 131.5, 128.7, 127.0, 123.3, 115.1, 51.8, 38.0, 35.0, 29.1, 28.6, 26.8, 25.7; **HRMS-ESI** (MeOH, m/z): $[M+Na]^+$ calcd for $C_{24}H_{27}N_2O_3$, 391.20162; found, 391.20123; **HPLC** (CHIRALCEL® OD3, *n*-heptane / EtOH = 85:15, 1 mL/min) t_R = 5.94 min (major), t_R = 7.21 min (minor), 84% *ee* (***R***); $[\alpha]_D^{25}$ = - 13.30 (c = 0.565, $CHCl_3$).

7 (*R*)-*N*-(5-phenoxypent-1-en-3-yl)benzamide **(1g)**

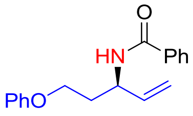

The reaction was performed with (penta-3,4-dien-1-yloxy)benzene (96.1 mg, 0.6 mmol). The crude product was purified by FCC on silica gel (EA/CH = 1/4, R_f = 0.26) to afford the product as a white solid (90.0 mg, 78 %).

m.p.: 94 – 95 °C; **^{1}H NMR** (400MHz, $CDCl_3$) δ = 7.85 - 7.79 (m, 2 H), 7.53 - 7.35 (m, 3 H), 7.32 - 7.25 (m, 2 H), 6.99 - 6.87 (m, 4 H), 5.92 (ddd, *J* = 5.3, 10.5, 17.2 Hz, 1 H), 5.27 (td, *J* = 1.4, 17.2 Hz, 1 H), 5.20 (td, *J* = 1.3, 10.5 Hz, 1 H), 5.00 - 4.89 (m, 1 H), 4.24 - 4.07 (m, 2 H), 2.32 - 2.22 (m, 1 H), 2.17 - 2.06 (m, 1 H); **^{13}C NMR** (100.6MHz, $CDCl_3$) δ = 166.8, 158.5, 137.3, 134.8, 131.5, 129.6, 128.6, 127.0, 121.2, 115.7, 114.6, 65.3, 50.4, 33.6; **HRMS-APCI** (MeOH, m/z): $[M+H]^+$ calcd for $C_{18}H_{20}NO_2$, 282.1489; found, 282.1490; **HPLC** (CHIRALCEL® AD-H, *n*-heptane / ^{i}PrOH = 90:10, 1 mL/min) t_R = 14.53 min (major), t_R = 16.34 min (minor), 97% *ee* (***R***); $[\alpha]_D^{25}$ = - 38.70 (c = 0.470, $CHCl_3$).

8 (*R*)-*N*-(6-(phenylthio)hex-1-en-3-yl)benzamide **(1h)**

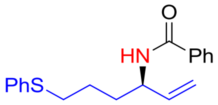

The reaction was performed with hexa-4,5-dien-1-yl(phenyl)sulfane (114.2 mg, 0.6 mmol). The crude product was purified by FCC on silica gel (EA/CH = 1/4, R_f = 0.20) to afford the product as a yellowish solid (74.7 mg, 60 %).

m.p.: 59 – 60 °C; **^{1}H NMR** (400MHz, $CDCl_3$) δ = 7.76 - 7.71 (m, 2 H), 7.52 - 7.47 (m, 1 H), 7.45 - 7.39 (m, 2 H), 7.33 - 7.30 (m, 2 H), 7.27 - 7.22 (m, 2 H), 7.17 - 7.13 (m, 1 H), 6.00 (d, *J* = 8.0 Hz, 1 H), 5.84 (ddd, *J* = 5.7, 10.5, 17.2 Hz, 1 H), 5.23 (ddd, *J* = 1.0, 1.6, 17.2 Hz, 1 H), 5.15 (td, *J* = 1.3, 10.4 Hz, 1 H), 4.75 - 4.62 (m, 1 H), 3.04 - 2.91 (m, 2 H), 1.90 - 1.68 (m, 4 H); **^{13}C NMR** (100.6MHz, $CDCl_3$) δ = 166.9, 138.1, 136.4, 134.7, 131.6, 129.4, 129.0, 128.7, 127.0, 126.1, 115.5, 51.4, 33.9, 33.6, 25.5; **HRMS-APCI** (MeOH, m/z): $[M+H]^+$ calcd for $C_{19}H_{22}NOS$, 312.14166; found, 312.14169; **HPLC** (CHIRALCEL® AD-3, *n*-heptane / EtOH = 85:15, 1 mL/min) t_R = 6.32 min (major), t_R = 6.90 min (minor), 96% *ee* (***R***); $[\alpha]_D^{25}$ = - 6.70 (c = 0.420, $CHCl_3$).

9 (*R*)-*N*-(6-(phenylsulfonyl)hex-1-en-3-yl)benzamide **(1i)**

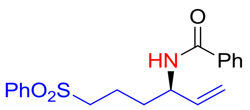

The reaction was performed with (hexa-4,5-dien-1-ylsulfonyl)benzene (133.4 mg, 0.6 mmol). The crude product was purified by FCC on silica gel (EA/CH = 1/2, R_f = 0.16) to afford the product as a yellowish oil (116.8 mg, 85 %).

^{1}H NMR (400MHz, $CDCl_3$) δ = 7.89 - 7.85 (m, 2 H), 7.75 - 7.71 (m, 2 H), 7.63 - 7.59 (m, 1 H), 7.54 - 7.49 (m, 3 H), 7.45 - 7.41 (m, 2 H), 6.08 (d, *J* = 8.7 Hz, 1 H), 5.82 (ddd, *J* = 5.8, 10.5, 17.2 Hz, 1 H), 5.26 - 5.20 (m, 1 H), 5.17 (td, *J* = 1.2, 10.4 Hz, 1 H), 4.67 - 4.58 (m, 1 H), 3.25 - 3.05 (m, 2 H), 1.93 - 1.61 (m, 4 H); **^{13}C NMR** (100.6MHz, $CDCl_3$) δ = 167.0, 139.2, 137.5, 134.4, 133.8, 131.7, 129.4, 128.7, 128.1, 127.0, 116.2, 55.7, 51.1, 33.3, 19.4; **HRMS-APCI** (MeOH, m/z): $[M+H]^+$ calcd for $C_{19}H_{22}NO_3S$, 344.13149; found, 344.13165; **HPLC** (CHIRALCEL® AD3, *n*-heptane / ^{i}PrOH = 85:15, 1 mL/min) t_R = 16.53 min (major), t_R = 17.96 min (minor), 93% *ee* (***R***); $[\alpha]_D^{25}$ = + 11.10 (c = 0.345, $CHCl_3$).

One-pot Synthesis of Allylic Amides

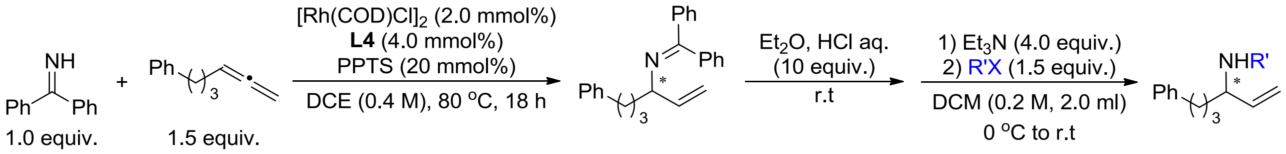

Step 1, **Hydroamination**: To a Schlenk tube was added $[Rh(COD)Cl]_2$ (3.94 mg, 0.008 mmol, 2 mol%), Ligand **L4** (9.70 mg, 0.016 mmol, 4 mol%), PPTS (20.1 mg, 0.08 mmol, 20%), benzophenone imine (72.5 mg, 0.4 mmol, 1.0 equiv), DCE (1.0 ml, 0.4 M) and Hexa-4,5-dien-1-ylbenzene (47.5 mg, 0.6 mmol, 1.5 equiv). The Schlenk tube was sealed and the mixture was stirred for 18 h at 80 °C. After cooling to room temperature, the solvent was removed under vacuum.

Step 2, **Hydrolysis**: To the hydroamination reaction mixture of *step 1* was added Et_2O (2.0 ml) and HCl aq. (2.0 ml, 2.0 M, 4 mmol) sequentially. The reaction was stirred at r.t for 24 hours. The volatiles were removed under vacuum.

Step 3, **Amide formation**: To the resulting allylic amine HCl salt curde mixture of *step 2* was added CH_2Cl_2 (2.0 ml) and Et_3N (223 µl, 161.9 mg, 1.6 mmol, 4.0 equiv) and corresponding acyl/sulfonyl chlorides or anhydride (0.6 mmol, 1.5 equiv) sequentially. The reaction mixture was stirred for 3 hours. The volatiles were removed and the residue was purified by FCC to give the desired allylic amides.

Synthesis and characterization of allylic amides (2a-d)

1 (*R*)-*tert*-butyl (6-phenylhex-1-en-3-yl)carbamate **(2a)**

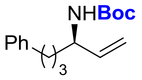

The reaction was performed with di-*tert*-butyl dicarbonate (130.9 mg, 0.6 mmol). The crude product was purified by FCC on silica gel (EA/CH = 1/20, R_f = 0.30) to afford the product as a white solid (88.2 mg, 80 %).

m.p.: 35 – 36 °C; **^{1}H NMR** (400MHz, $CDCl_3$) δ = 7.30 - 7.25 (m, 2 H), 7.21 - 7.15 (m, 3 H), 5.73 (ddd, *J* = 5.7, 10.4, 17.1 Hz, 1 H), 5.14 (td, *J* = 1.4, 17.2 Hz, 1 H), 5.08 (td, *J* = 1.4, 10.4 Hz, 1 H), 4.41 (br. s., 1 H), 4.21 - 4.04 (m, 1 H), 2.70 - 2.57 (m, 2 H), 1.73 - 1.64 (m, 2 H), 1.62 - 1.47 (m, 2 H), 1.45 - 1.44 (m, 9 H); **^{13}C NMR** (100.6MHz, $CDCl_3$) δ = 155.5, 142.3, 139.1, 128.5, 128.4, 125.9, 114.5, 35.7, 34.9, 28.5, 27.6; **HRMS-APCI** (MeOH, m/z): $[M+NH_4]^+$ calcd for $C_{17}H_{29}N_2O_2$, 293.22235; found, 293.22235; **HPLC** (CHIRALCEL® L-A2, *n*-heptane / EtOH = 98:2, 1 mL/min) t_R = 10.14 min (major), t_R = 12.74 min (minor), 95% *ee* (***R***); $[\alpha]_D^{25}$ = - 13.90 (c = 0.392, $CHCl_3$).

2 (*R*)-4-methyl-*N*-(6-phenylhex-1-en-3-yl)benzenesulfonamide **(2b)**

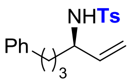

The reaction was performed with 4-methylbenzene-1-sulfonyl chloride (114.0 mg, 0.6 mmol). The crude product was purified by FCC on silica gel (EA/CH = 1/4, R_f = 0.40) to afford the product as a white solid (98.8 mg, 75 %).

m.p.: 40 – 41 °C; **^{1}H NMR** (400MHz, $CDCl_3$) δ = 7.74 - 7.69 (m, 2 H), 7.29 - 7.23 (m, 4 H), 7.20 - 7.15 (m, 1 H), 7.11 - 7.06 (m, 2 H), 5.53 (ddd, *J* = 6.4, 10.5, 17.1 Hz, 1 H), 5.00 - 4.93 (m, 2 H), 4.50 - 4.36 (m, 1 H), 3.85 - 3.73 (m, 1 H), 2.56 - 2.50 (m, 2 H), 2.41 (s, 3 H), 1.66 - 1.45 (m, 4 H); **^{13}C NMR** (101MHz, $CDCl_3$) δ = 143.3, 141.9, 138.3, 137.8, 129.6, 128.45, 128.42, 127.3, 125.9, 116.1, 56.3, 35.4, 35.2, 27.1, 21.6; **HRMS-ESI** (MeOH, m/z): $[M+Na]^+$ calcd for $C_{19}H_{23}NO_2SNa$, 352.13417; found, 352.13437; **HPLC** (CHIRALCEL® AD-3, *n*-heptane / EtOH = 90:10, 1 mL/min) t_R = 7.94 min (major), t_R = 9.65 min (minor), 95% *ee* (***R***); $[\alpha]_D^{25}$ = - 20.00 (c = 0.522, $CHCl_3$).

3 (*R*)-*N*-(6-phenylhex-1-en-3-yl)acrylamide **(2c)**

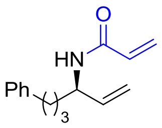

The reaction was performed with acryloyl chloride (54.0 mg, 0.6 mmol). The crude product was purified by FCC on silica gel (EA/CH = 1/2, R_f = 0.36) to afford the product as a yellowish oil (64.2 mg, 70 %).

^{1}H NMR (400MHz, $CDCl_3$) δ = 7.35 - 7.27 (m, 2 H), 7.21 - 7.14 (m, 3 H), 6.28 (dd, *J* = 1.4, 16.9 Hz, 1 H), 6.12 - 6.04 (m, 1 H), 5.76 (ddd, *J* = 5.8, 10.4, 17.2 Hz, 1 H), 5.64 (dd, *J* = 1.5, 10.3 Hz, 1 H), 5.38 (br. s., 1 H), 5.21 - 5.14 (m, 1 H), 5.11 (td, *J* = 1.3, 10.4 Hz, 1 H), 4.63 - 4.53 (m, 1 H), 2.67 - 2.61 (m, 2 H), 1.72 - 1.55 (m, 4 H); **^{13}C NMR** (100.6MHz, $CDCl_3$) δ = 164.9, 142.1, 138.2, 131.0, 128.53, 128.46, 126.6, 126.0, 115.3, 51.4, 35.7, 34.5, 27.6; **HRMS-ESI** (MeOH, m/z): $[M+H]^+$ calcd for $C_{15}H_{20}NO$, 230.15394; found, 230.15413; **HPLC** (CHIRALCEL® L-A2, *n*-heptane / EtOH = 95:5, 1 mL/min) t_R = 17.09 min (major), t_R = 23.61 min (minor), 95% *ee* (***R***); $[\alpha]_D^{25}$ = + 13.30 (c = 0.541, $CHCl_3$).

4 (*R*)-*N*-(6-phenylhex-1-en-3-yl)pent-4-enamide **(2d)**

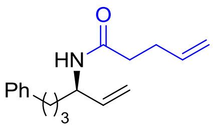

The reaction was performed with pent-4-enoyl chloride (71.1 mg, 0.6 mmol). The crude product was purified by FCC on silica gel (EA/CH = 1/2, R_f = 0.32) to afford the product as a yellowish oil (77.2 mg, 75 %).

^{1}H NMR (400MHz, $CDCl_3$) δ = 7.30 - 7.26 (m, 2 H), 7.20 - 7.14 (m, 3 H), 5.88 - 5.69 (m, 2 H), 5.32 (d, *J* = 8.3 Hz, 1 H), 5.16 - 5.04 (m, 3 H), 5.00 (tdd, *J* = 1.2, 2.0, 10.2 Hz, 1 H), 4.51 (ttdd, *J* = 1.5, 6.0, 7.4, 8.8 Hz, 1 H), 2.70 - 2.56 (m, 2 H), 2.44 - 2.36 (m, 2 H), 2.31 - 2.24 (m, 2 H), 1.71 - 1.47 (m, 4 H); **^{13}C NMR** (100.6MHz, $CDCl_3$) δ = 171.6, 142.1, 138.5, 137.2, 128.5, 128.4, 125.9, 115.8, 115.0, 51.1, 36.2, 35.7, 34.5, 29.8, 27.6;

HRMS-ESI (MeOH, m/z): $[M+H]^+$ calcd for $C_{17}H_{24}NO$, 258.18524; found, 258.18546; **HPLC** (CHIRALCEL® AD-3, *n*-heptane / EtOH = 97:3, 1 mL/min) t_R = 11.33 min (major), t_R = 15.00 min (minor), 95% *ee* (***R***); $[\alpha]_D^{25}$ = -8.20 (c = 0.511, $CHCl_3$).

Hydroamination of Bioactive Moieties Containing Substrates

Following the scope conditions, late-stage hydroamination with bioactive moiety containing substrates resulted in the desired branched allylic amines (**3a-d**). The *ee* values of compounds (**3a**, **3b**, **3c** and **3d**) were measured via their derivatives (**3aee**, **3bee**, **3cee** and **3dee** respectively), which were derived from **(3a-3d)** according to the literature procedure via transesterification with ethanol.[5]

1 (*R*)-(1*R*,2*S*,5*R*)-2-isopropyl-5-methylcyclohexyl 4-benzamidohex-5-enoate **(3a)**

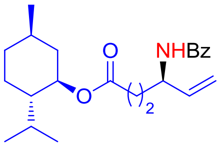

The reaction was performed with (1*R*,2*S*,5*R*)-2-isopropyl-5-methylcyclohexyl hexa-4,5-dienoate (150.2 mg, 0.6 mmol). The crude product was purified by FCC on silica gel (EA/CH = 1/4, R_f = 0.30) to afford the product as a yellowish oil (116.0 mg, 78 %).

^{1}H NMR (500MHz, $CDCl_3$, mixture of diastereoisomers) δ = 7.82 - 7.78 (m, 2 H), 7.51 - 7.47 (m, 1 H), 7.45 - 7.40 (m, 2 H), 6.48 (d, *J* = 7.3 Hz, 1 H), 5.86 (ddd, *J* = 5.6, 10.4, 17.2 Hz, 1 H), 5.29 - 5.23 (m, 1 H), 5.18 (td, *J* = 1.3, 10.4 Hz, 1 H), 4.73 - 4.65 (m, 2 H), 2.51 - 2.37 (m, 2 H), 2.07 - 1.95 (m, 3 H), 1.85 - 1.73 (m, 1 H), 1.71 - 1.63 (m, 2 H), 1.53 - 1.43 (m, 1 H), 1.39 - 1.32 (m, 1 H), 1.09 - 0.92 (m, 2 H), 0.91 - 0.89 (m, 3 H), 0.86 - 0.83 (m, 3 H), 0.67 (d, *J* = 7.0 Hz, 3 H); **^{13}C NMR** (126MHz, $CDCl_3$, mixture of diastereoisomers) δ = 173.7, 166.9, 137.7, 134.5, 131.6, 128.6, 127.1, 115.7, 74.8, 51.9, 47.0, 41.0, 34.3, 31.5, 31.3, 29.2, 26.4, 23.5, 22.1, 20.8, 16.3; **HRMS-APCI** (MeOH, m/z): $[M+H]^+$ calcd for $C_{23}H_{34}O_3N$, 372.25332; found, 372.25345. $[\alpha]_D^{25}$ = - 16.20 (c = 0.420, $CHCl_3$).

The *ee* value of **3a** was measured via its derivative (*R*)-ethyl 4-benzamidohex-5-enoate (**3aee**).

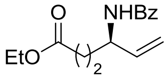

^{1}H NMR (400MHz, $CDCl_3$) δ = 7.83 - 7.78 (m, 2 H), 7.53 - 7.41 (m, 3 H), 6.45 (d, *J* = 7.8 Hz, 1 H), 5.91 - 5.80 (m, 1 H), 5.29 - 5.22 (m, 1 H), 5.20 - 5.16 (m, 1 H), 4.76 - 4.66 (m, 1 H), 4.17 - 4.03 (m, 2 H), 2.56 - 2.37 (m, 2 H), 2.10 - 1.92 (m, 2 H), 1.20 (t, *J* = 7.1 Hz, 3 H); **^{13}C NMR** (101MHz, $CDCl_3$) δ = 174.1, 166.8, 137.7, 134.5, 131.6, 128.7, 127.0, 115.7, 60.8, 51.8, 31.0, 29.3, 14.3; **HRMS-APCI** (MeOH, m/z): $[M+H]^+$ calcd for $C_{15}H_{20}O_3N$, 262.14377; found, 262.14383. **HPLC** (CHIRALCEL® OD3, n-Heptane / ^{i}PrOH = 90:10, 1.0 mL/min) t_R = 6.12 min (major), t_R = 7.60 min (minor), 92% *ee* (***R***); $[\alpha]_D^{25}$ = - 14.70 (c = 0.380, $CHCl_3$).

2 (*R*)-(1*R*,2*S*,5*R*)-2-isopropyl-5-methylcyclohexyl 5-benzamidohept-6-enoate **(3b)**

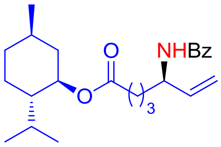

The reaction was performed with (1*R*,2*S*,5*R*)-2-isopropyl-5-methylcyclohexyl hepta-5,6-dienoate (158.6 mg, 0.6 mmol). The crude product was purified by FCC on silica gel (EA/CH = 1/4, R_f = 0.30) to afford the product as a yellowish oil (140.3 mg, 91 %).

^{1}H NMR (500MHz, $CDCl_3$, mixture of diastereoisomers) δ = 7.82 - 7.77 (m, 2 H), 7.52 - 7.47 (m, 1 H), 7.45 - 7.41 (m, 2 H), 6.19 (d, *J* = 7.9 Hz, 1 H), 5.85 (ddd, *J* = 5.6, 10.4, 17.2 Hz, 1 H), 5.24 (td, *J* = 1.3, 17.2 Hz, 1 H), 5.16 (td, *J* = 1.3, 10.4 Hz, 1 H), 4.72 - 4.63 (m, 2 H), 2.39 - 2.29 (m, 2 H), 2.00 - 1.93 (m, 1 H), 1.87 - 1.80 (m, 1 H), 1.79 - 1.62 (m, 5 H), 1.54 - 1.43 (m, 1 H), 1.39 - 1.31 (m, 1 H), 1.09 - 0.92 (m, 2 H), 0.91 - 0.72 (m, 10 H); **^{13}C NMR** (126MHz, $CDCl_3$, mixture of diastereoisomers) δ = 173.1, 167.0, 138.1, 134.7, 131.6, 128.7, 127.0, 115.4, 74.3, 51.6, 47.1, 41.1, 34.4, 34.2, 31.5, 26.4, 23.5, 22.1, 21.3, 20.8, 16.4; **HRMS-APCI** (MeOH, m/z): $[M+H]^+$ calcd for $C_{24}H_{36}O_3N$, 386.26897; found, 386.26892. $[\alpha]_D^{25}$ = - 13.10 (c = 0.477, $CHCl_3$).

The *ee* value of **3b** was measured via its derivative (*R*)-ethyl 5-benzamidohept-6-enoate **(3bee)**.

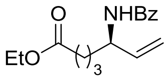

^{1}H NMR (400MHz, $CDCl_3$) δ = 7.82 - 7.78 (m, 2 H), 7.52 - 7.48 (m, 1 H), 7.46 - 7.41 (m, 2 H), 6.13 (d, *J* = 8.1 Hz, 1 H), 5.86 (ddd, *J* = 5.7, 10.4, 17.2 Hz, 1 H), 5.29 - 5.22 (m, 1 H), 5.16 (td, *J* = 1.3, 10.4 Hz, 1 H), 4.73 - 4.63 (m, 1 H), 4.13 (q, *J* = 7.1 Hz, 2 H), 2.40 - 2.33 (m, 2 H), 1.79 - 1.59 (m, 4 H), 1.25 (t, *J* = 7.1 Hz, 3 H); **^{13}C NMR** (101MHz, $CDCl_3$) δ = 173.5, 167.0, 138.2, 134.8, 131.6, 128.7, 127.0, 115.5, 60.5, 51.6, 34.2, 33.9, 21.2, 14.4; **HRMS-APCI** (MeOH, m/z): $[M+H]^+$ calcd for $C_{16}H_{22}O_3N$, 276.15942; found, 276.15930. **HPLC** (CHIRALCEL® L-C1, n-Heptane / ^{i}PrOH = 95:5, 1.0 mL/min) t_R = 17.02 min (major), t_R = 20.81 min (minor), 97% *ee* (***R***); $[\alpha]_D^{25}$ = - 12.00 (c = 0.500, $CHCl_3$).

3 (*R*)-(1*S*,2*R*,4*S*)-1,7,7-trimethylbicyclo[2.2.1]heptan-2-yl 5-benzamidohept-6-enoate **(3c)**

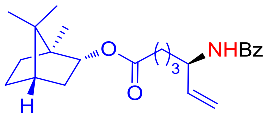

The reaction was performed with (1*S*,2*R*,4*S*)-1,7,7-trimethylbicyclo[2.2.1]heptan-2-yl hepta-5,6-dienoate (157.4 mg, 0.6 mmol). The crude product was purified by FCC on silica gel (EA/CH = 1/4, R_f = 0.23) to afford the product as a yellowish oil (130.4 mg, 85 %).

^{1}H NMR (500MHz, $CDCl_3$, mixture of diastereoisomers) δ = 7.83 - 7.77 (m, 2 H), 7.52 - 7.47 (m, 1 H), 7.45 - 7.39 (m, 2 H), 6.45 - 6.37 (m, 1 H), 5.87 - 5.74 (m, 1 H), 5.26 - 5.10 (m, 2 H), 4.69 - 4.59 (m, 2 H), 2.38 - 2.24 (m, 2 H), 1.83 - 1.46 (m, 9 H), 1.16 - 0.99 (m, 2 H), 0.91 (s, 3 H), 0.80 - 0.77 (m, 6 H); **^{13}C NMR** (126MHz, $CDCl_3$, mixture of diastereoisomers) δ = 173.0, 167.0, 138.1, 134.7, 131.5, 128.6, 127.0, 115.4, 81.1, 51.6, 48.7, 47.0,

45.1, 38.9, 34.3, 34.2, 33.8, 27.1, 21.3, 20.2, 20.0, 11.6; **HRMS-APCI** (MeOH, m/z): $[M+Na]^+$ calcd for $C_{24}H_{33}O_3NNa$, 406.23527; found, 406.23532. $[\alpha]_D^{25}$ = - 13.20 (c = 0.431, $CHCl_3$).

The *ee* value of **3c** was measured via its derivative **3cee**.

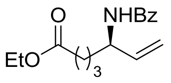

HPLC (CHIRALCEL® L-C1, n-Heptane / ^{i}PrOH = 95:5, 1.0 mL/min) t_R = 16.66 min (major), t_R = 20.39 min (minor), 95% *ee* (***R***); $[\alpha]_D^{25}$ = - 14.30 (c = 0.560, $CHCl_3$).

4 (*R*)-(3*R*,5*R*,8*S*,9*R*,10*R*,13*S*,14*R*,17*S*)-10,13-dimethyl-17-((*S*)-6-methylheptan-2-yl)hexadecahydro-1*H*-cyclopenta[a]phenanthren-3-yl 5-benzamidohept-6-enoate **(3d)**

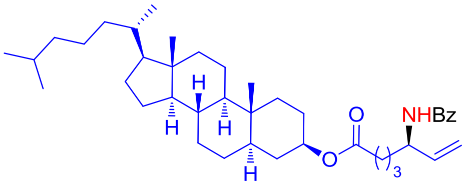

The reaction was performed with (3*R*,5*R*,8*S*,9*R*,10*R*,13*S*,14*R*,17*S*)-10,13-dimethyl-17-((*S*)-6-methylheptan-2-yl)hexadecahydro-1*H*-cyclopenta[a]phenanthren-3-yl hepta-5,6-dienoate (298.1 mg, 0.6 mmol). The crude product was purified by FCC on silica gel (EA/CH = 1/4, R_f = 0.30) to afford the product as a yellowish oil (173.0 mg, 70 %).

^{1}H NMR (500MHz, $CDCl_3$, mixture of diastereoisomers) δ = 7.84 - 7.79 (m, 2 H), 7.54 - 7.49 (m, 1 H), 7.47 - 7.41 (m, 2 H), 6.43 - 6.36 (m, 1 H), 5.88 - 5.77 (m, 1 H), 5.27 - 5.10 (m, 2 H), 4.72 - 4.58 (m, 2 H), 3.62 - 3.52 (m, 1 H), 2.39 - 2.25 (m, 2 H), 1.96 - 1.88 (m, 2 H), 1.81 - 0.57 (m, 47 H); **^{13}C NMR** (126MHz, $CDCl_3$, mixture of diastereoisomers) δ = 173.1, 167.0, 138.2, 134.7, 131.6, 128.7, 127.0, 115.4, 73.9, 71.5, 56.6, 56.5, 56.4, 56.4, 54.5, 54.3, 51.6, 45.0, 44.8, 42.7, 40.2, 40.1, 39.6, 38.4, 37.1, 36.9, 36.3, 35.9, 35.6, 35.6, 34.2, 34.2, 34.2, 32.2, 32.1, 31.7, 28.9, 28.7, 28.4, 28.1, 27.6, 24.3, 24.3, 24.0, 22.9, 22.7, 21.4, 21.3, 21.3, 18.8, 12.5, 12.3, 12.2; **HRMS-APCI** (MeOH, m/z): $[M+H]^+$ calcd for $C_{41}H_{64}O_3N$, 618.48807; found, 618.48755. $[\alpha]_D^{25}$ = - 42.10 (c = 0.421, $CHCl_3$).

The *ee* value of **3d** was measured via its derivative **3dee**.

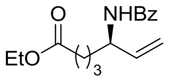

HPLC (CHIRALCEL® L-C1, n-Heptane / ^{i}PrOH = 95:5, 1.0 mL/min) t_R = 16.66 min (major), t_R = 2.36 min (minor), 96% *ee* (***R***); $[\alpha]_D^{25}$ = - 36.40 (c = 0.440, $CHCl_3$).

Large Scale Synthesis of Primary Allylic Amines

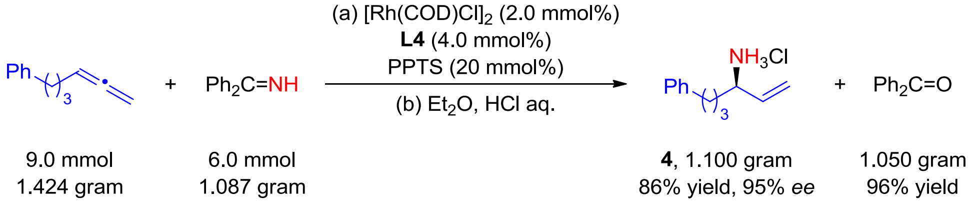

Step 1, **Hydroamination**: To a Schlenk tube was added $[Rh(COD)Cl]_2$ (59.2 mg, 0.12 mmol, 2 mol%), Ligand **L4** (145.6 mg, 0.24 mmol, 4 mol%), PPTS (301.6 mg, 1.2 mmol, 20%), benzophenone imine (1.09 g, 6.0 mmol, 1.0 equiv), DCE (15 ml, 0.4 M) and hexa-4,5-dien-1-ylbenzene (1.42 g, 9.0 mmol, 1.5 equiv). The Schlenk tube was sealed and the mixture was stirred for 18 h at 80 ℃. After cooling to room temperature, the solvent was removed under vacuum.

Step 2, **Hydrolysis**: To the hydroamination reaction mixture of *step 1* was added Et_2O (30 ml) and HCl aq. (30 ml, 2.0 M, 4 mmol) sequentially. The reaction was stirred at r.t for 24 hours. The volatiles were removed under vacuum.

Step 3, **Purification**: The two phase mixture was separated, the aqueous phase was extracted with Et_2O (20 ml x 3). 1) The combined organic phase was concentrated via rotary evaporation, and the organic residue was purified via FCC to recycle the benzophenone (EA/CH = 1/15, 1.05 g, 96% yield). 2) Water in the aqueous phase was removed via azeotropic distillation with toluene. The resulted solid was washed with cold Et_2O then pentane to obtain the desired HCl salt (*R*)-6-phenylhex-1-en-3-aminium chloride (**4**) as a white solid (1.10 g, 86% yield). The *ee* value of compound **4** was measured via its amide derivative (**4ee**) after benzoyl protection following the scope condition. The enantiomeric excess of compound **4ee** is the same with **1e** (95% *ee*).

m.p.: 135-136 °C; **^{1}H NMR** (400MHz, $CDCl_3$) δ = 8.58 (br. s., 3 H), 7.31 - 7.23 (m, 2 H), 7.21 - 7.12 (m, 3 H), 5.86 (ddd, *J* = 7.6, 10.1, 17.3 Hz, 1 H), 5.44 (d, *J* = 17.2 Hz, 1 H), 5.35 (d, *J* = 10.5 Hz, 1 H), 3.71 (m, 1 H), 2.70 - 2.55 (m, 2 H), 2.01 - 1.91 (m, 1 H), 1.87 - 1.79 (m, 1 H), 1.77 - 1.68 (m, 2 H); **^{13}C NMR** (101MHz, $CDCl_3$) δ = 141.5, 133.4, 128.5, 128.5, 126.1, 121.0, 54.6, 35.4, 33.0, 27.2; **HRMS-ESI** (MeOH, m/z): $[M-Cl]^+$ calcd for $C_{12}H_{18}N$, 176.14338; found, 176.14349. $[\alpha]_D^{25}$ = - 10.80 (c = 0.300, $CHCl_3$).

Derivatization of Allylic Amides

1 Synthesis of (*R*)-*tert*-butyl (1-hydroxy-5-phenylpentan-2-yl)carbamate (**5a**)

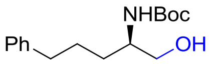

The reaction was performed according to the modified literature procedure.[6a] Allylic amine **2a** (110.2 mg, 0.4 mmol) was dissolved in CH_2Cl_2 (4.0 ml, 0.1 M) and cooled to – 78 °C. Ozone was directly bubbled in the solution. When the solution remained light blue about 5 min, indicating excess ozone, the ozone flow was stopped and N_2 was bubbled for 15 min to remove excess of ozone. The solvent was removed via rotary evaporation, the resulted ozonide was dissolved in methanol (4.0 ml), then $NaBH_4$ (454.0 mg, 12 mmol) was added at 0 °C. The resulting slurry was stirred at r.t. for 10 hours, then HCl (aq. 1.0 M) was slowly added to the mixture at 0 °C to quenched excess $NaBH_4$. Water and Et_2O were added and the aqueous phase was separated, and extracted with Et_2O. The combined organic phases were washed with brined, $MgSO_4$, then filtered and concentrated under vacuum. The crude product was purified by FCC on silica gel (EA/CH = 1/4, R_f = 0.33) to afford the product as a white solid (100.6 mg, 90 %).

m.p.: 67 – 68 °C; **^{1}H NMR** (500MHz, $CDCl_3$) δ = 7.30 - 7.25 (m, 2 H), 7.21 - 7.14 (m, 3 H), 4.62 (d, *J* = 7.9 Hz, 1 H), 3.65 (d, *J* = 8.7 Hz, 2 H), 3.58 - 3.48 (m, 1 H), 2.70 - 2.57 (m, 2 H), 1.77 - 1.62 (m, 2 H), 1.60 - 1.51 (m, 1 H), 1.49 - 1.40 (m, 10 H); **^{13}C NMR** (126MHz, $CDCl_3$) δ = 156.6, 142.1, 128.5, 128.4, 125.9, 79.8, 66.1, 52.8, 35.7, 31.2, 28.0; **HRMS-ESI** (MeOH, m/z): $[M+Na]^+$ calcd for $C_{16}H_{25}NO_3Na$, 302.17266; found, 302.17282; **HPLC** (CHIRALCEL® LC-1, *n*-heptane / ^{i}PrOH = 95:5, 1 mL/min) t_R = 8.95 min (minor), t_R = 10.15 min (major), 95% *ee* (***R***); $[\alpha]_D^{25}$ = - 4.50 (c = 0.375, $CHCl_3$).

2 Synthesis of (*R*)-*tert*-butyl (1-oxo-5-phenylpentan-2-yl)carbamate (**5b**)

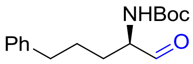

The reaction was performed according to the modified literature procedure.[6a] Allylic amine **2a** (110.2 mg, 0.4 mmol) was dissolved in CH_2Cl_2 (4.0 ml, 0.1 M) and cooled to – 78 °C. Ozone was directly bubbled in the solution. When the solution remained light blue about 5 min, indicating excess ozone, the ozone flow was stopped and N_2 was bubbled for 15 min to remove excess of ozone. To the reaction mixture was added PPh_3 (104.9 mg, 0.4 mmol) at – 78 °C, and the resulting solution was stirred at r.t. for 2 hours. The crude reaction mixture was concentrated and purified by FCC on silica gel (EA/CH = 1/4, R_f = 0.33) to afford the product as a yellowish solid (94.3 mg, 85 %). The *ee* value of **5b** was obtained according to the *ee* of its derivative (**5a**).

m.p.: 50 – 51 °C; **^{1}H NMR** (500MHz, $CDCl_3$) δ = 9.55 (s, 1 H), 7.30 - 7.26 (m, 2 H), 7.21 - 7.14 (m, 3 H), 5.06 (m, 1 H), 4.25 (m, 1 H), 2.73 - 2.57 (m, 2 H), 1.96 - 1.86 (m, 1 H), 1.78 - 1.64 (m, 2 H), 1.62 - 1.53 (m, 1 H), 1.45 (s, 9 H); **^{13}C NMR** (126MHz, $CDCl_3$) δ = 199.8, 155.6, 141.5, 128.5, 128.4, 126.1, 80.2, 59.8, 35.5, 28.8, 28.4, 27.0; **HRMS-ESI** (MeOH, m/z): $[M+Na]^+$ calcd for $C_{16}H_{23}NO_3Na$, 300.15701; found, 300.15714; $[\alpha]_D^{25}$ = - 137.60 (c = 0.300, $CHCl_3$).

3 Synthesis of (*R*)-2-((tert-butoxycarbonyl)amino)-5-phenylpentanoic acid (**5c**)

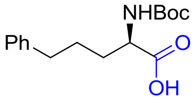

The reaction was performed according to the modified literature procedure.[6b] Allylic amine **2a** (70.0 mg, 0.25 mmol) was dissolved in a mixture of $CH_3CN/CCl_4/H_2O$ (2.0 ml/2.0 ml/3.0 ml), then $NaIO_4$ (219.2 mg, 1.025 mmol, 4.1 equiv) and ruthenium trichloride hydrate (1.1 mg, 0.0055 mmol, 2.2%) were added subsequently. The reaction mixture was vigorously stirred at room temperature for 2 hours. The CH_2Cl_2 (10 ml) was added and the phases were separated. The upper aqueous phase was extracted three times with CH_2Cl_2. The combined organic extracts were dried over $MgSO_4$, filtered and concentrated. The crude mixture was purified by FCC on silica gel (CH_3OH/CH_2Cl_2 = 1/16, R_f = 0.30) to afford the product as a yellowish oil (60.0 mg, 82 %). The *ee* value of **5c** was obtained according to the *ee* of its derivative (**5a**).

^{1}H NMR (400MHz, $CDCl_3$) δ = 7.30 - 7.25 (m, 2 H), 7.21 - 7.14 (m, 3 H), 4.94 (br. s., 1 H), 4.41 - 4.26 (m, 1 H), 2.72 - 2.57 (m, 2 H), 1.97 - 1.83 (m, 1 H), 1.80 - 1.63 (m, 3 H), 1.44 (s, 9 H); **^{13}C NMR** (101MHz, $CDCl_3$) δ = 177.2, 155.8, 141.7, 128.5, 126.1, 80.4, 53.3, 35.4, 32.0, 28.4, 27.2; **HRMS-APCI** (MeOH, m/z): $[M+NH_4]^+$ calcd for $C_{16}H_{27}N_2O_4$, 311.19653; found, 311.19638; $[\alpha]_D^{25}$ = + 3.10 (c = 0.315, $CHCl_3$).

4 Synthesis of (*R*)-*tert*-butyl (1-hydroxy-6-phenylhexan-3-yl)carbamate (**5d**)

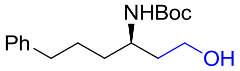

The reaction was performed according to the modified literature procedure.[6a] Allylic amine **2a** (251.0 mg, 0.9 mmol) was dissolved in THF (10 ml) and cooled to – 78 °C, then 9-BBN (4.6 ml, 272.5 mg, 2.25 mmol, 0.5 M in THF) was slowly added. The reaction was stirred for 15 min at 0 °C, and then slowly warmed up to room temperature, at which it was stirred overnight. The reaction mixture was cooled to – 10 °C, then EtOH (1.0 ml), NaOH (1.0 ml, 2 M) and H_2O_2 (1.0 ml, 30% in H_2O) were added slowly in the given order. The reaction was warmed up to room temperature and stirred for 3 hours. The reaction mixture was diluted with dichloromethane and transferred into a separation funnel. The aqueous phase was extracted with dichloromethane (3x), the combined organic layers were dried over Na_2SO_4, filtered and concentrated by rotary evaporation. The crude product was purified by FCC on silica gel (EA/CH = 1/2, R_f = 0.33) to afford the product as a white solid (252.0 mg, 95 %).

m.p.: 67 – 68 °C; **^{1}H NMR** (400MHz, $CDCl_3$) δ = 7.31 - 7.25 (m, 2 H), 7.22 - 7.14 (m, 3 H), 4.31 (br. s., 1 H), 3.85 - 3.72 (m, 1 H), 3.67 - 3.55 (m, 2 H), 2.72 - 2.27 (m, 3 H), 1.87 - 1.61 (m, 3 H), 1.57 - 1.35 (m, 11 H), 1.34 - 1.20 (m, 1 H); **^{13}C NMR** (101MHz, $CDCl_3$) δ = 157.2, 142.1, 128.50, 128.46, 125.9, 79.9, 58.9, 47.3, 39.2, 35.7, 35.3, 28.5, 28.1; **HRMS-ESI** (MeOH, m/z): $[M]^+$ calcd for $C_{17}H_{27}NO_3Na$, 316.18831; found, 316.18848; **HPLC** (CHIRALCEL® AD-3, *n*-heptane / EtOH = 90:10, 1 mL/min) t_R = 3.79 min (minor), t_R = 4.58 min (major), 95% *ee* (***R***); $[\alpha]_D^{25}$ = - 6.40 (c = 0.375, $CHCl_3$).

5 Synthesis of (*R*)-tert-butyl (1-oxo-6-phenylhexan-3-yl)carbamate (**5e**)

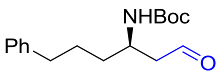

The reaction was performed according to the modified literature procedure.[6c] To a solution of **5d** (58.7 mg, 0.2 mmol) in DMSO (0.5 ml) was added Et_3N (80 µl, 0.6 mmol). The solution was cooled to 0 °C and sulfur trioxide pyridine complex (95.0 mg, 0.6 mmol) in DMSO (0.5 ml) was added. The mixture was stirred at r.t. for 1 hour, then poured into ice-water and extracted with ethyl acetate. The combined organic layers were washed with acetic acid (10%), water, $NaHCO_3$ (5%) and brine. The organic residue was dried over $MgSO_4$, filtered and concentrated. The crude reaction mixture was purified by FCC on silica gel (EA/CH = 1/4, R_f = 0.30) to afford the product as a colorless oil (51.3 mg, 88 %). The *ee* value of **5e** was obtained according to the *ee* of its derivative (**5d**).

^{1}H NMR (400MHz, $CDCl_3$) δ = 9.75 - 9.73 (m, 1 H), 7.30 - 7.24 (m, 2 H), 7.21 - 7.14 (m, 3 H), 4.71 - 4.53 (m, 1 H), 4.12 - 3.99 (m, 1 H), 2.69 - 2.50 (m, 4 H), 1.78 - 1.51 (m, 4 H), 1.43 (s, 9 H); **^{13}C NMR** (101MHz, $CDCl_3$) δ = 201.1, 155.5, 142.0, 128.5, 126.0, 79.7, 49.3, 46.5, 35.5, 34.7, 28.5, 28.0; **HRMS-APCI** (MeOH, m/z): $[M+H]^+$ calcd for $C_{17}H_{26}NO_3$, 292.19072; found, 292.19061; $[\alpha]_D^{25}$ = + 10.00 (c = 0.350, $CHCl_3$).

6 Synthesis of (*R*)-3-((*tert*-butoxycarbonyl)amino)-6-phenylhexanoic acid (**5f**)

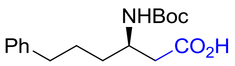

The reaction was performed according to the modified literature procedure.[6d] A mixture of **5c** (234.7 mg, 0.8 mmol), $PhI(OAc)_2$ (515.4 mg, 1.6 mmol), and TEMPO (37.5 mg, 0.24 mmol) was stirred in aq. CH_3CN (1/1, 1.5 ml) at room temperature overnight. The mixture was extracted with ethyl acetate, then the organic phase was extracted with aq. $NaHCO_3$. The aqueous solution was acidified to pH 3 using aq. Citric acid (5%), then extracted with EA. The combined organic phases was dried over $MgSO_4$ and concentrated. The crude product was purified by FCC on silica gel ($MeOH/CH_2Cl_2$ = 1/16, R_f = 0.30) to afford the product as a white solid (118.0 mg, 48 %). The *ee* value of **5f** was obtained according to the *ee* of its precursor (**5d**).

m.p.: 109 – 110 °C; **^{1}H NMR** (400MHz, $CDCl_3$) δ = 7.30 - 7.25 (m, 2 H), 7.20 - 7.14 (m, 3 H), 4.89 (br. s., 1 H), 4.05 - 3.77 (m, 1 H), 2.72 - 2.44 (m, 4 H), 1.77 - 1.52 (m, 4 H), 1.44 (s, 9 H); **^{13}C NMR** (101MHz, $CDCl_3$) δ = 176.6, 155.8, 142.1, 128.5, 128.4, 125.9, 79.7, 47.4, 39.4, 35.6, 34.2, 28.5, 28.0; **HRMS-ESI** (MeOH, m/z): $[M]^+$ calcd for $C_{17}H_{25}NO_4Na$, 330.16758; found, 330.16776; $[\alpha]_D^{25}$ = + 7.70 (c = 0.330, $CHCl_3$).

Mechanistic Investigations

1 Isotopic-labeling experiments

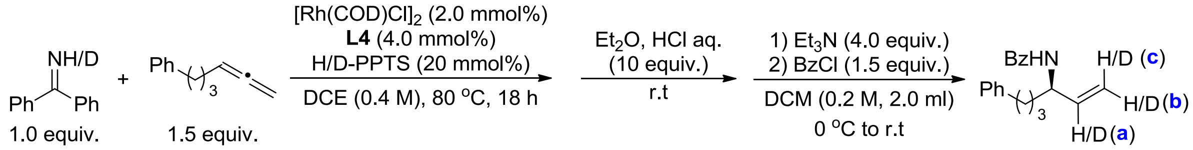

The reactions were performed according to the scope procedure using deuterated samples. The crude products were purified by flash column chromatography on silica gel (EA / CH = 1:30, R_f = 0.35) to afford the deuterated products.

2 Results

Nr.	Ph_2C=NH/D	H/D-PPTS	Yield	Deuterium Distribution
1	Ph_2C=NH	D-PPTS	84%	H_a = 8%, H_b = H_c = 0
2	Ph_2C=ND	H-PPTS	79%	H_a = 29%, H_b = H_c = 0
3	Ph_2C=ND	D-PPTS	75%	H_a = 34%, H_b = H_c = 0
4	Ph_2C=ND	-	34%	H_a = 32%, H_b = H_c = 0

3 ^{1}H NMR of isotopic labeling experiments

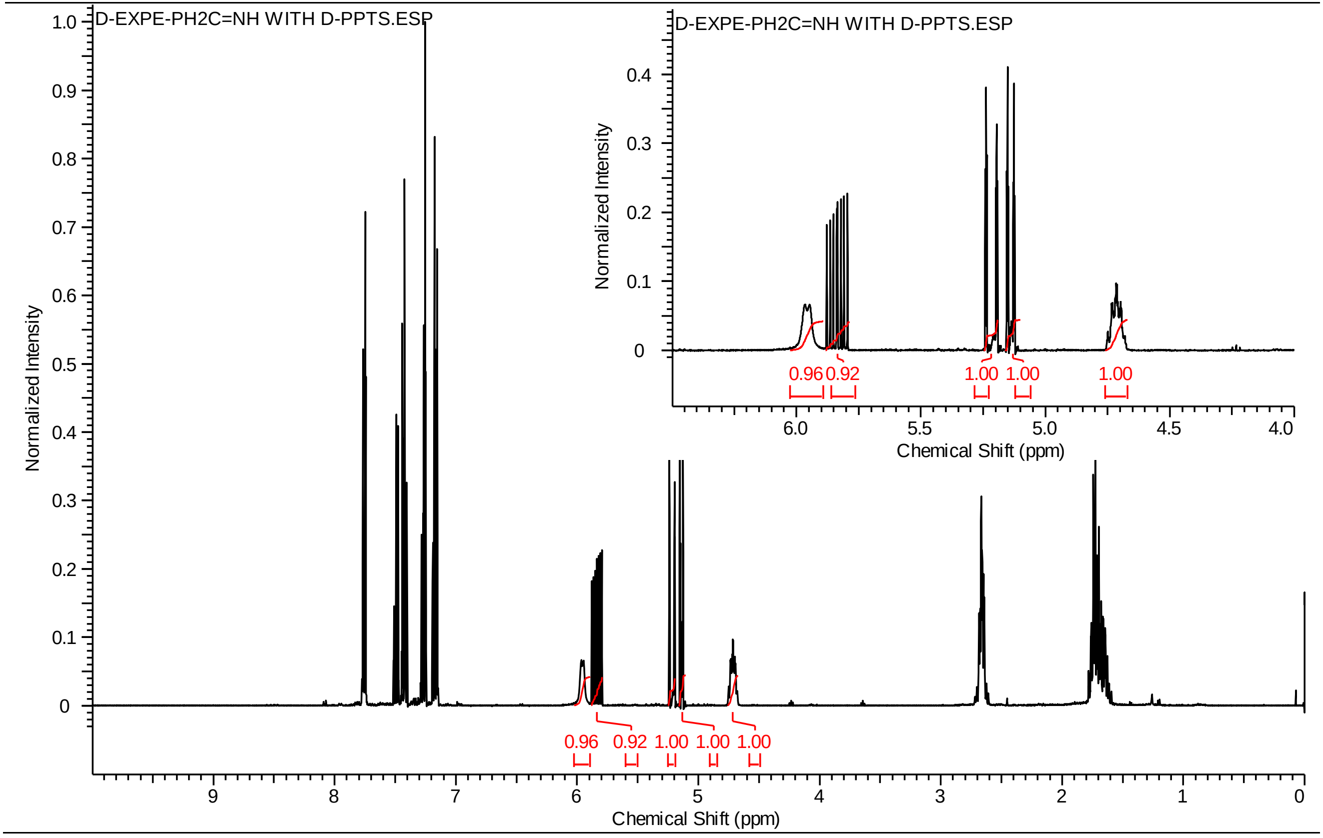

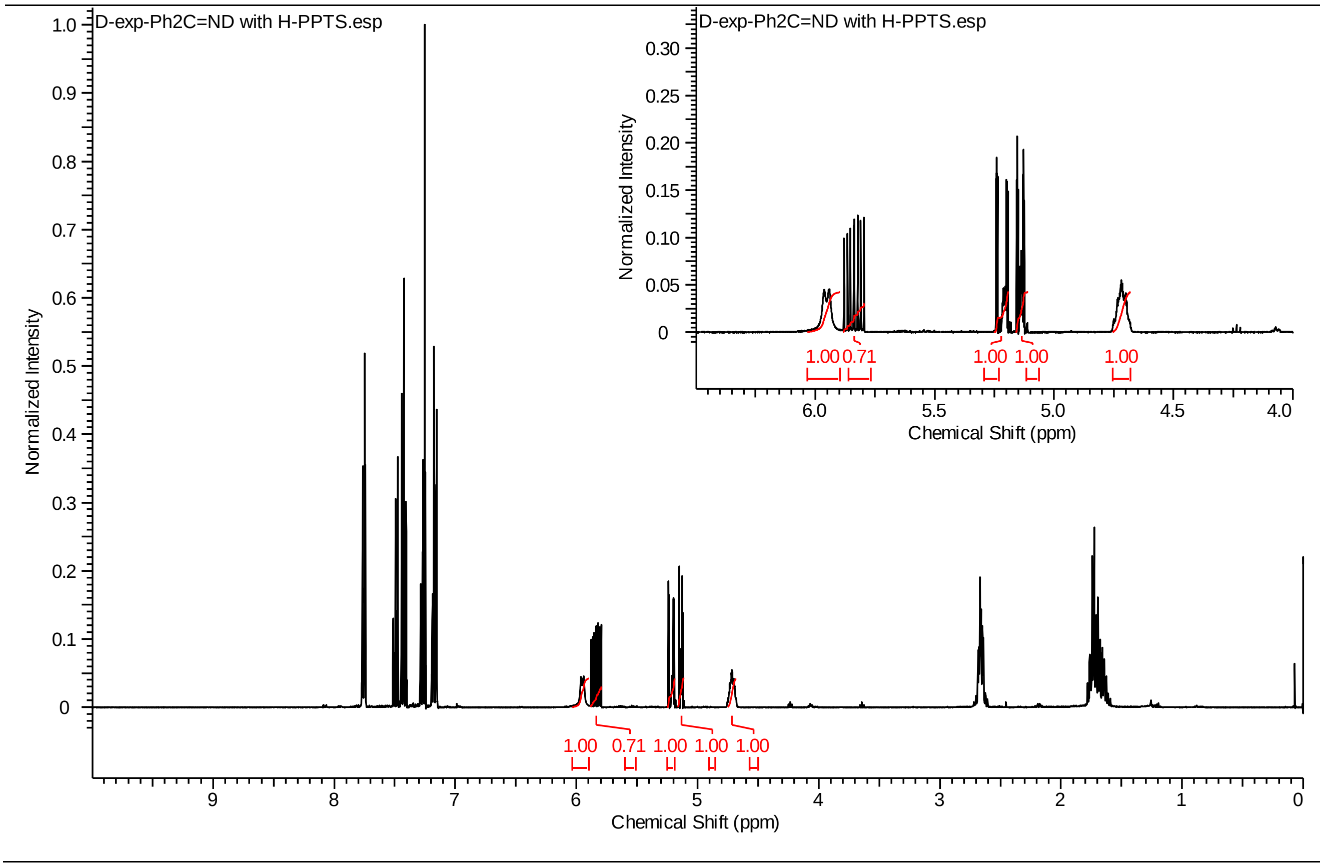
D-exp-Ph2C=ND with H-PPTS.esp
Normalized Intensity
Chemical Shift (ppm)
1.00
0.71
1.00
1.00
1.00
D-exp-Ph2C=ND with H-PPTS.esp
1.00
0.71
1.00
1.00
1.00

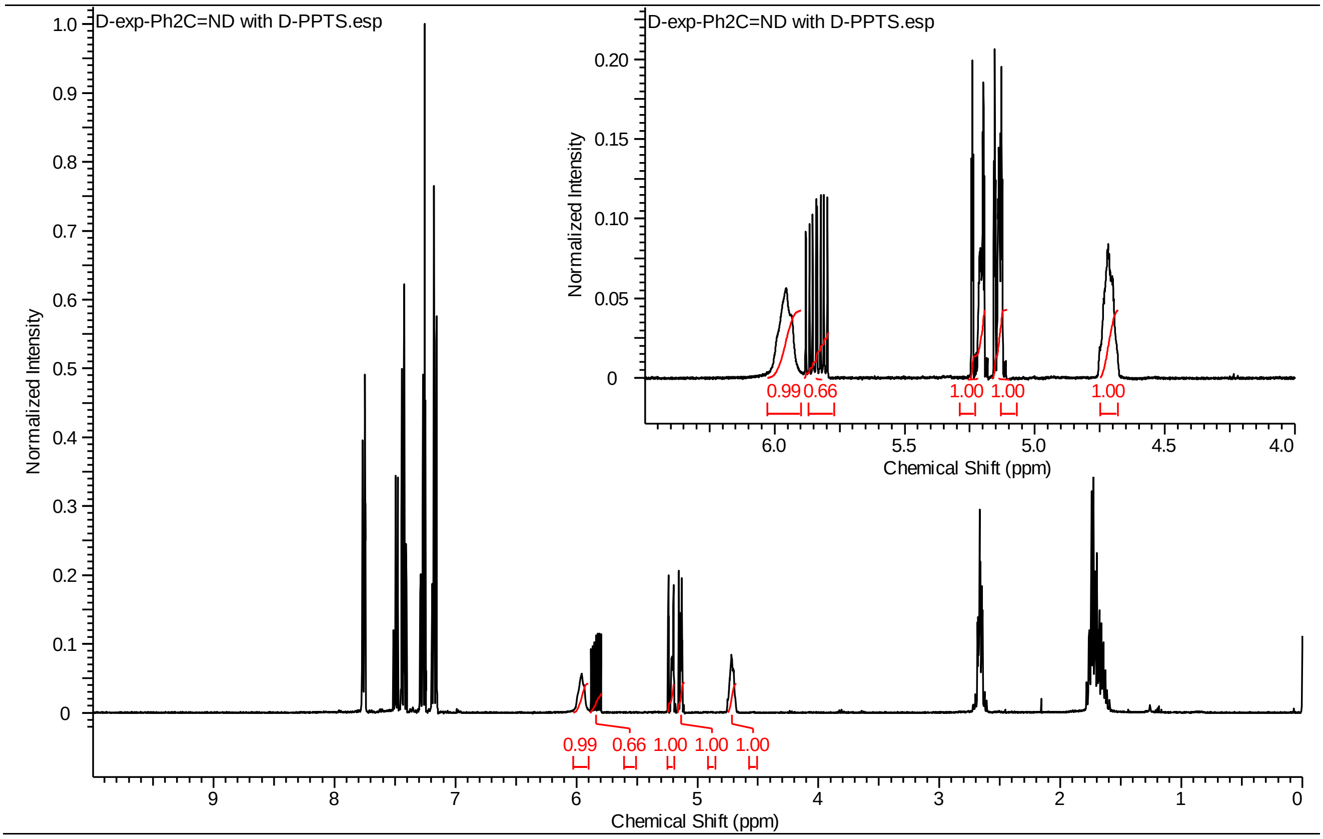
D-exp-Ph2C=ND with D-PPTS.esp
Normalized Intensity
Chemical Shift (ppm)
0.99
0.66
1.00
1.00
1.00
D-exp-Ph2C=ND with D-PPTS.esp
0.99
0.66
1.00
1.00
1.00

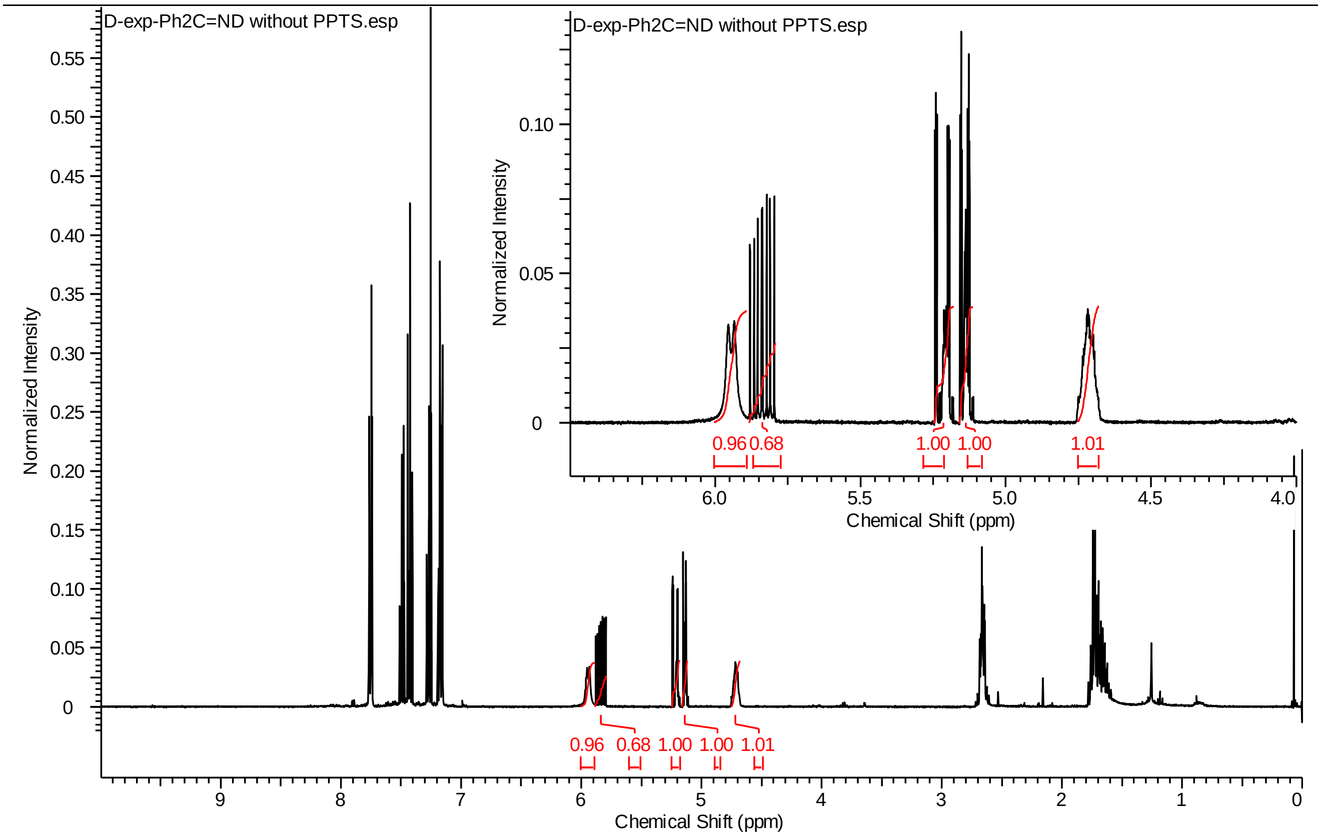

4 Proposed mechanism

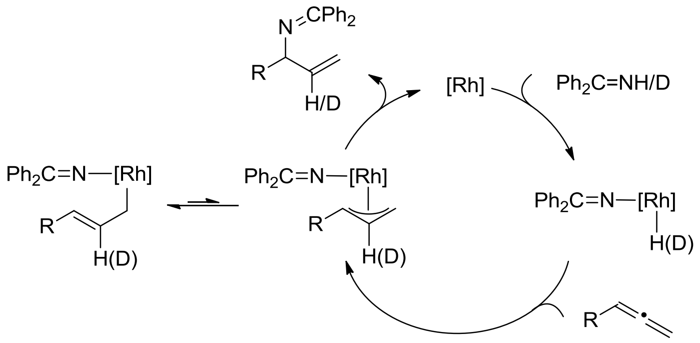

References

1 a) Kuang, J.; Ma, S. *J. Org. Chem*. **1999**, *74*, 1763-1765. b) Xu, K.; Thieme, N.; Breit, B. *Angew. Chem. Int. Ed*. **2014**, *53*, 2162-2165.

2 Hou, Z.; Yoda, C.; Koizumi, T.; Nishiura, M.; Wakatsuki, Y.; Fukuzawa, S.; Takats, J. *Organometallics.* **2003**, *22*, 3586-3592.

3 Manley, D. W.; Walton, J. C. *Org. Lett*. **2014**, *16*, 5394-5397.

4 Lafrance, M.; Roggen, M.; Carreira, E. M. *Angew. Chem. Int. Ed*. **2012**, *51*, 3470-3473.

5 Veitch, G. E.; Bridgwood, K. L.; Ley, S. V. *Org. Lett*. **2008**, *10*, 3623-3625.

6 a) Xu, K.; Thieme, N.; Breit, B. *Angew. Chem. Int. Ed*. **2014**, *53*, 7268-7271. b) Singh, O. V.; Han, H. *Tetrahedron Lett*. **2007**, *48*, 7094-7098. c) Panek, J. S.; Masse, C. E. *J. Org. Chem*. **1997**, *62*, 8290-8291. d) Bollans, L.; Bacsa, J.; O'Farrell, D. A.; Waterson, S.; Stachulski, A. V. *Tetrahedron. Lett*. **2010**, *51*, 2160-2163.

^{1}H NMR and ^{13}C NMR spectra

(*S*)-*N*-(1-cyclohexylallyl)benzamide **(1a)**

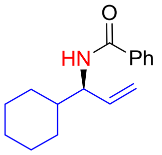

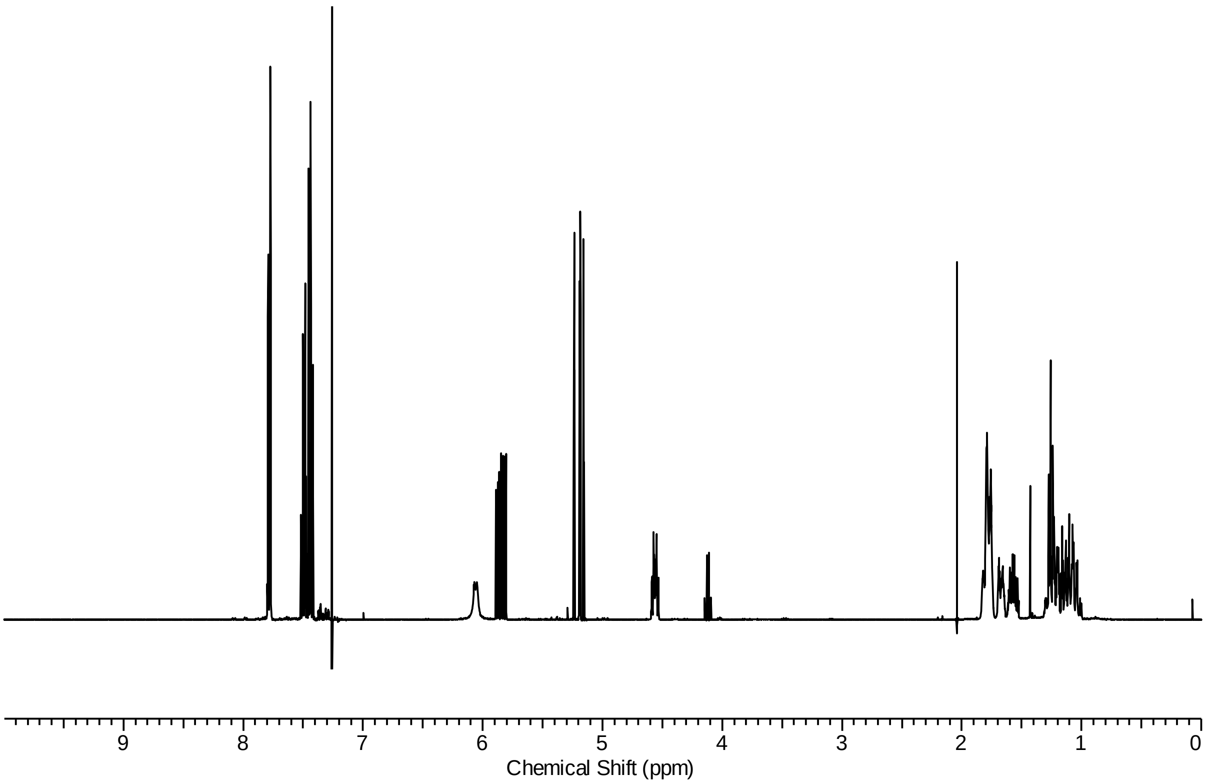

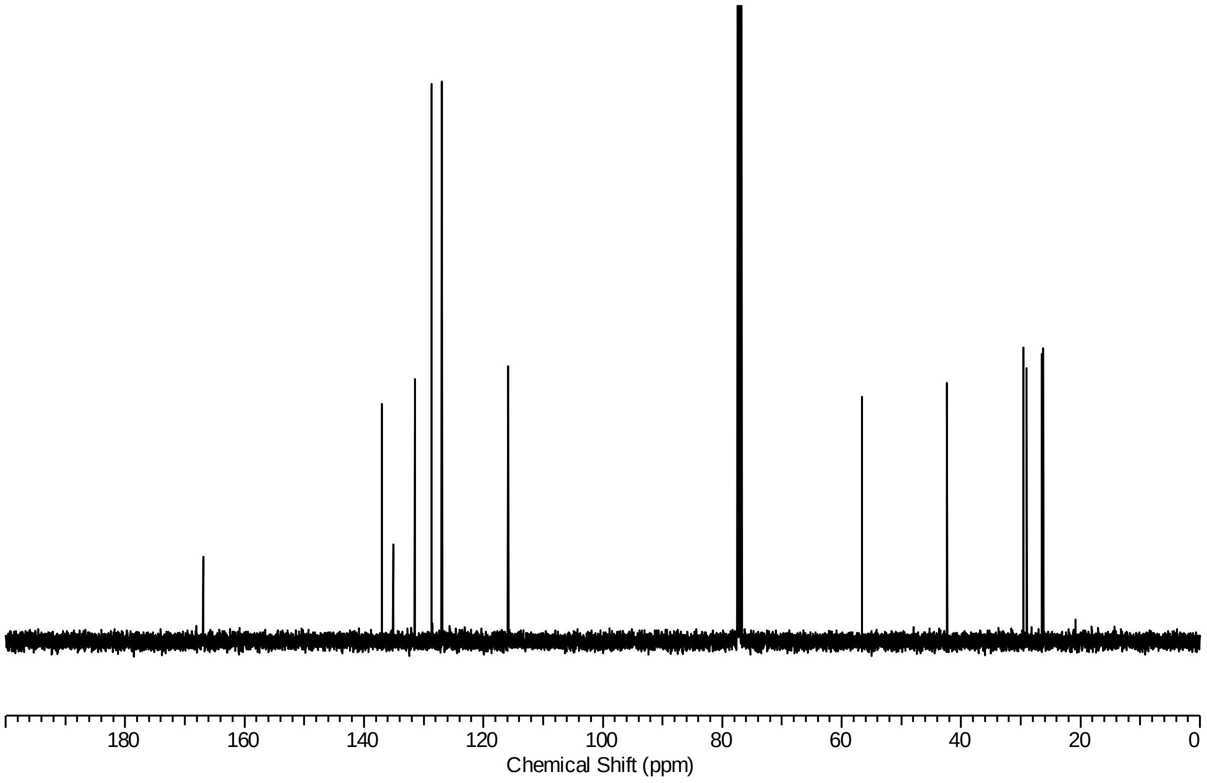

(*S*)-*N*-(1-cyclopentylallyl)benzamide **(1b)**

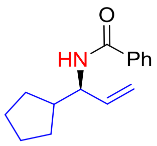

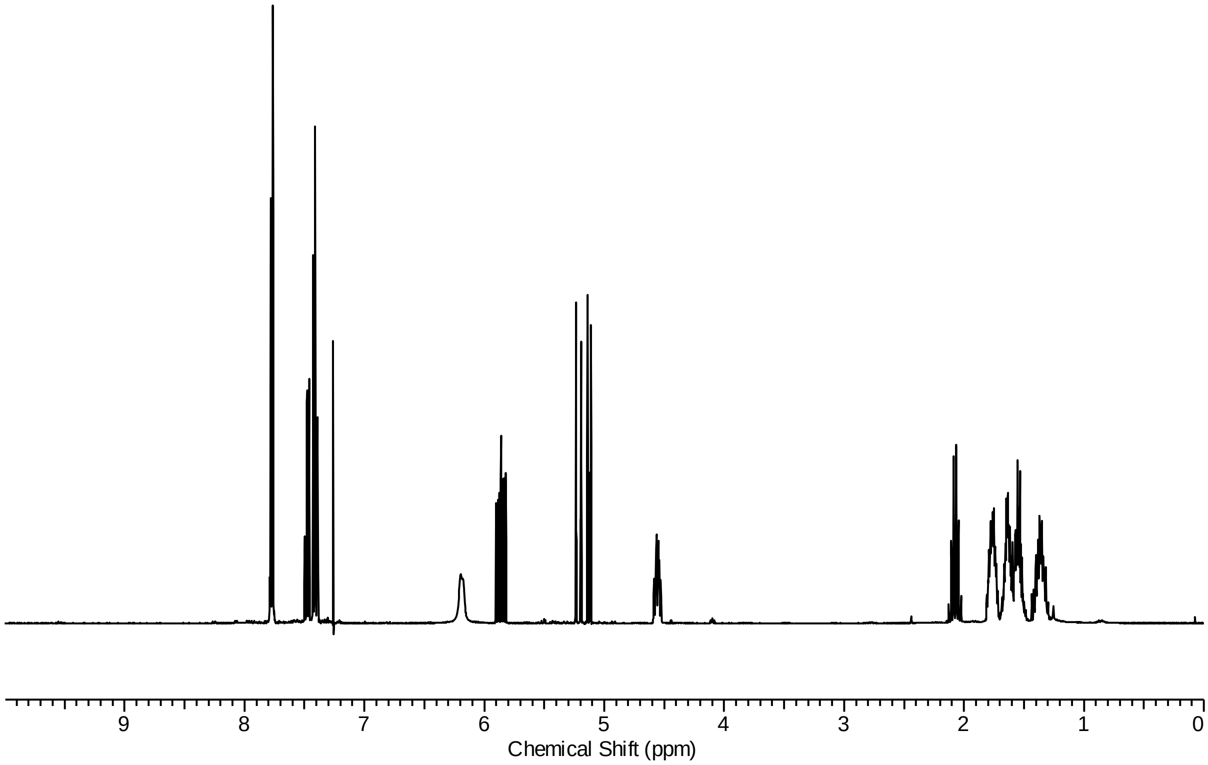

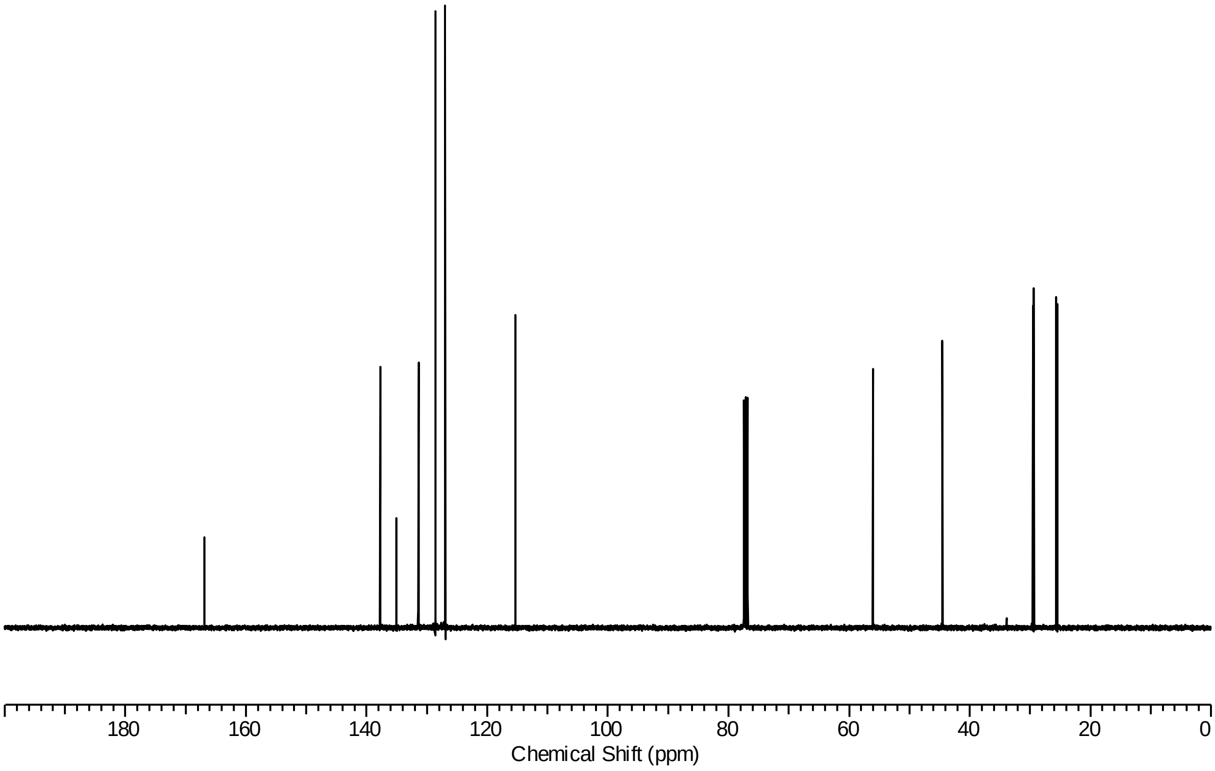

(*R*)-*N*-(hexadec-1-en-3-yl)benzamide **(1c)**

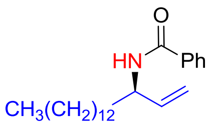

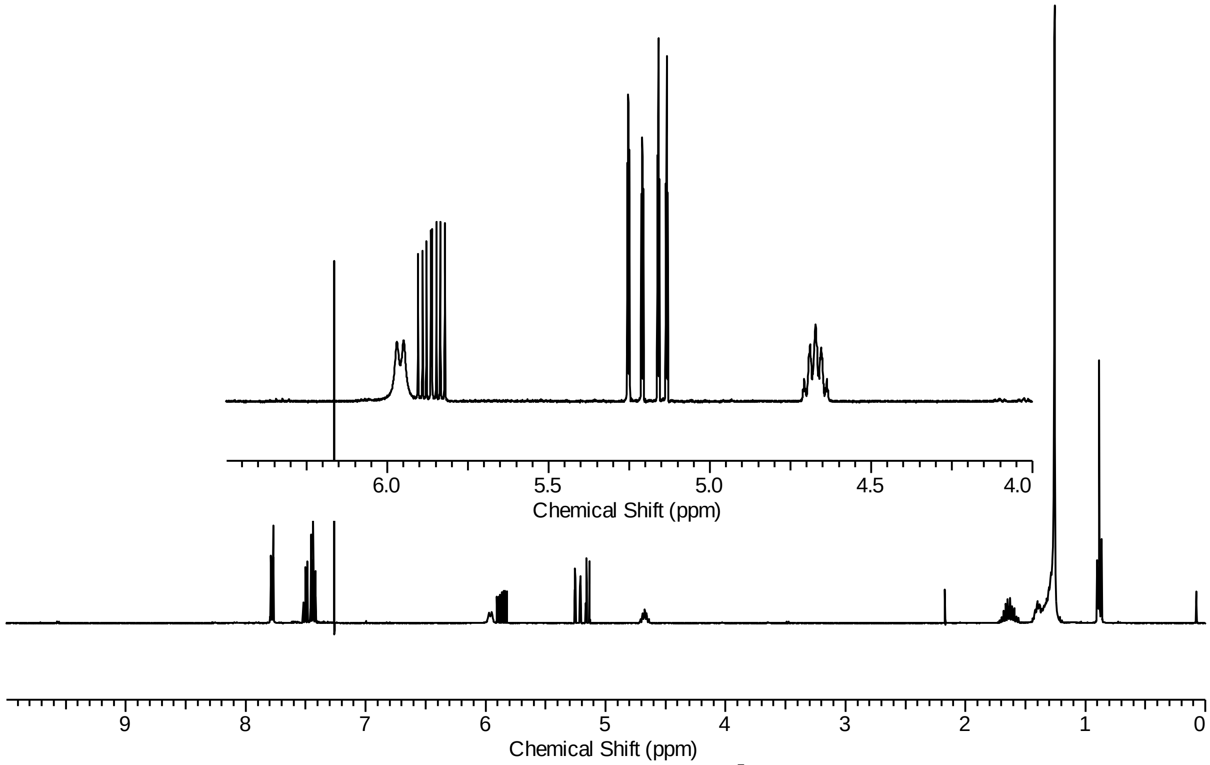

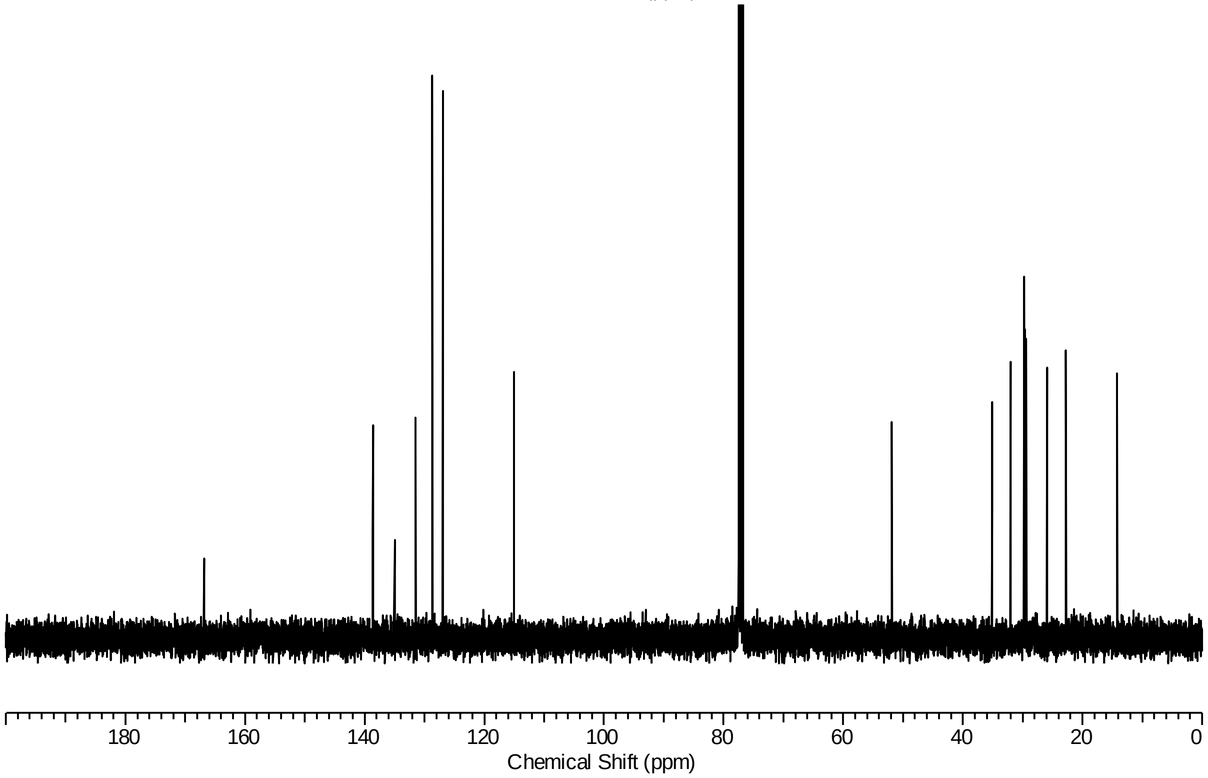

(*R*)-*N*-(5-phenylpent-1-en-3-yl)benzamide **(1d)**

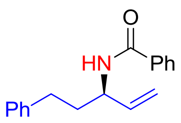

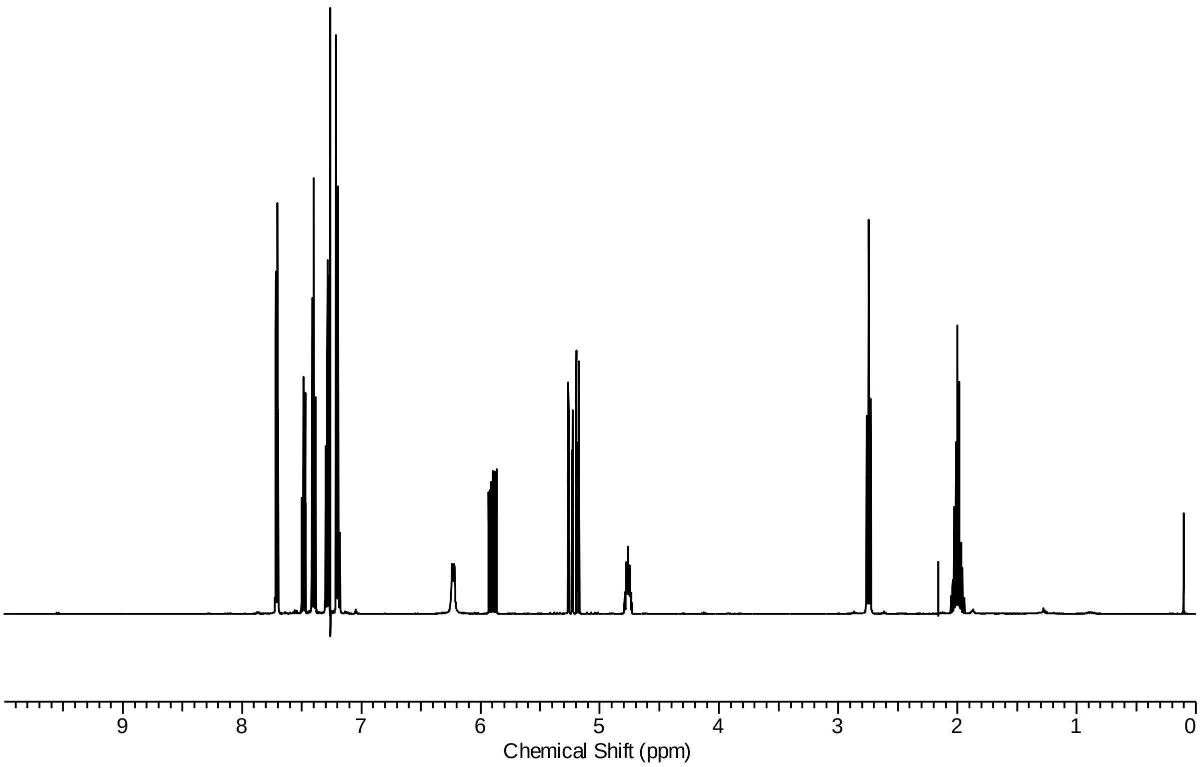

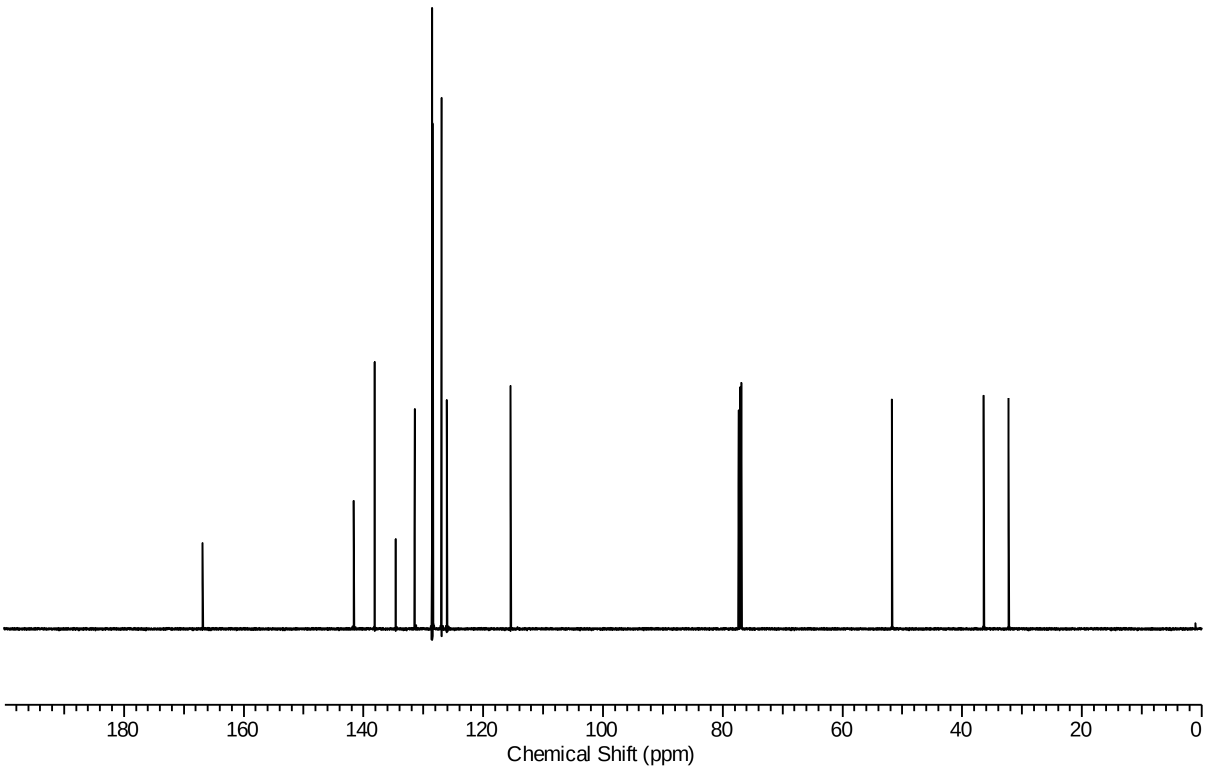

(*R*)-*N*-(6-phenylhex-1-en-3-yl)benzamide **(1e)**

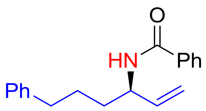

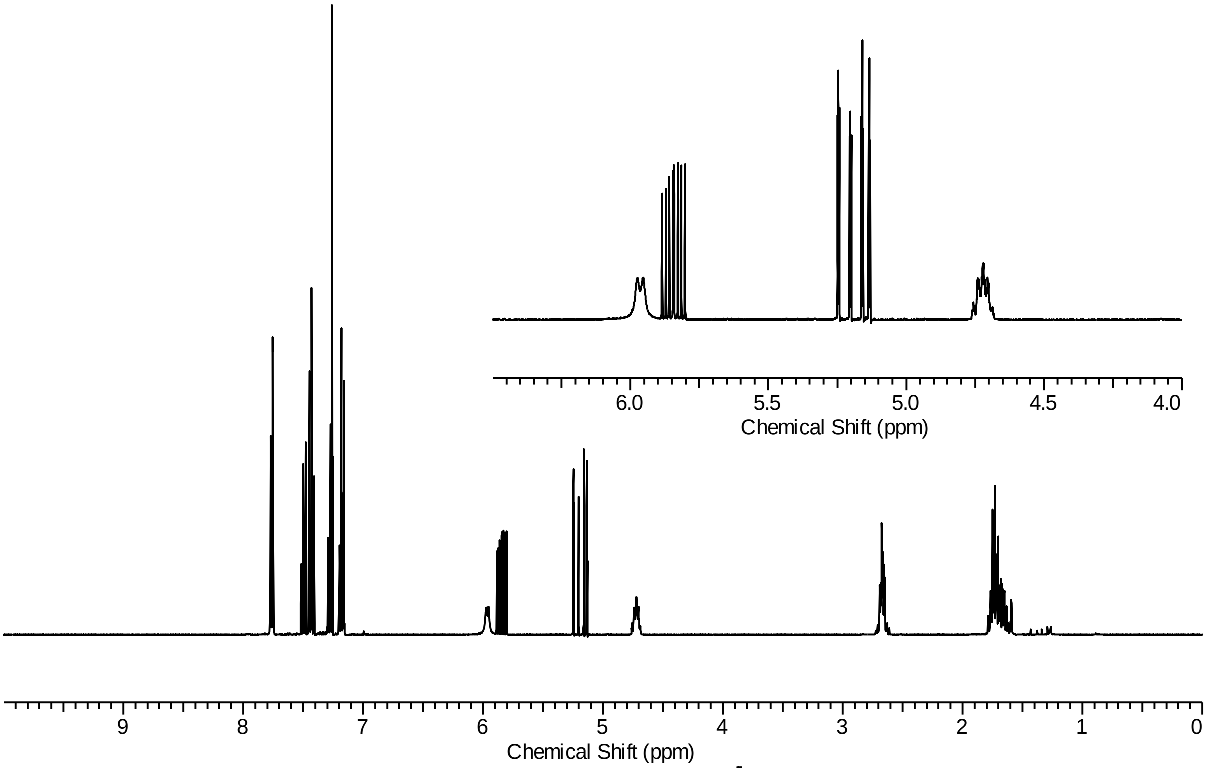

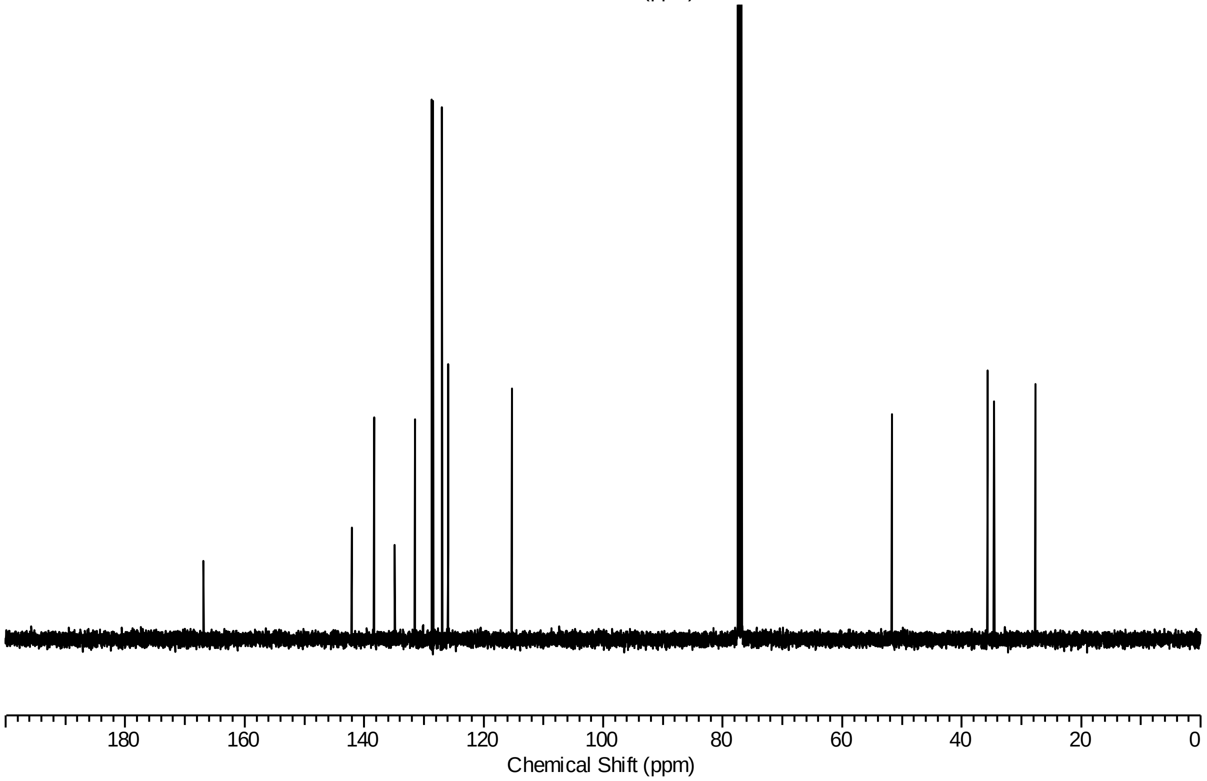

(*R*)-*N*-(9-(1,3-dioxoisoindolin-2-yl)non-1-en-3-yl)benzamide **(1f)**

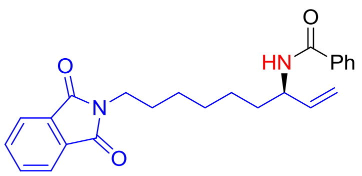

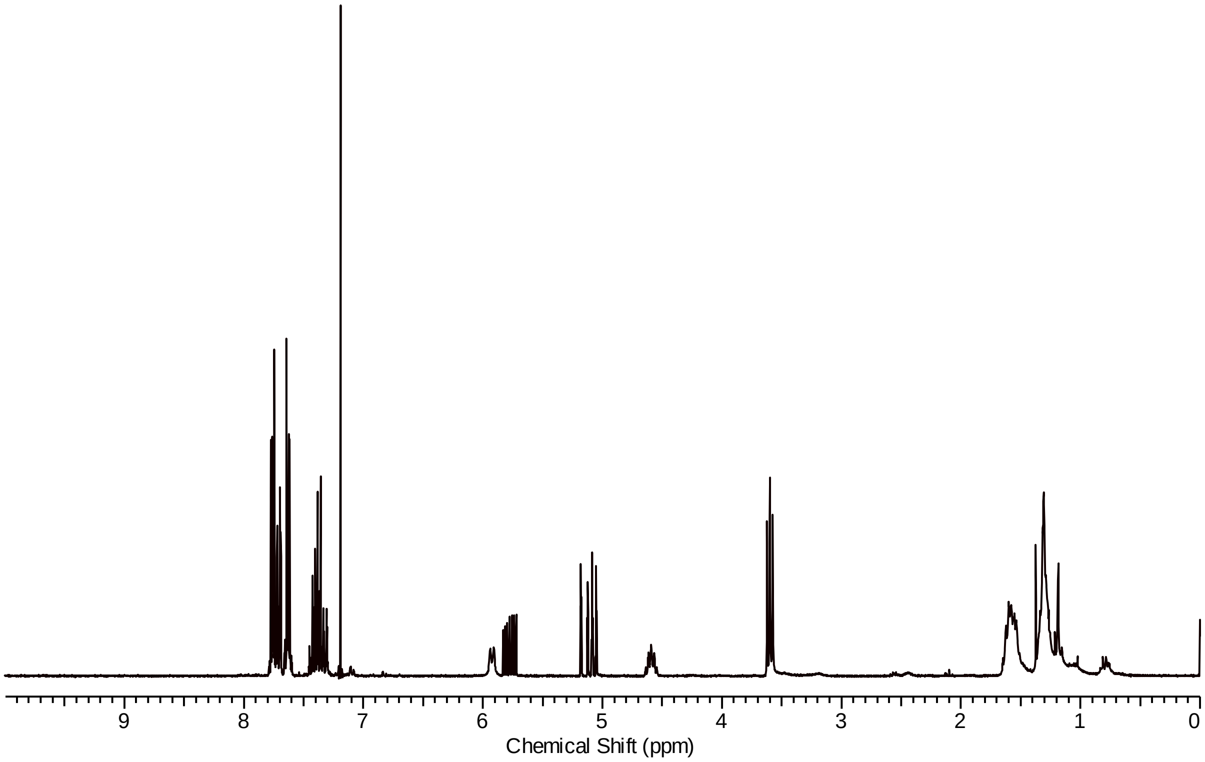

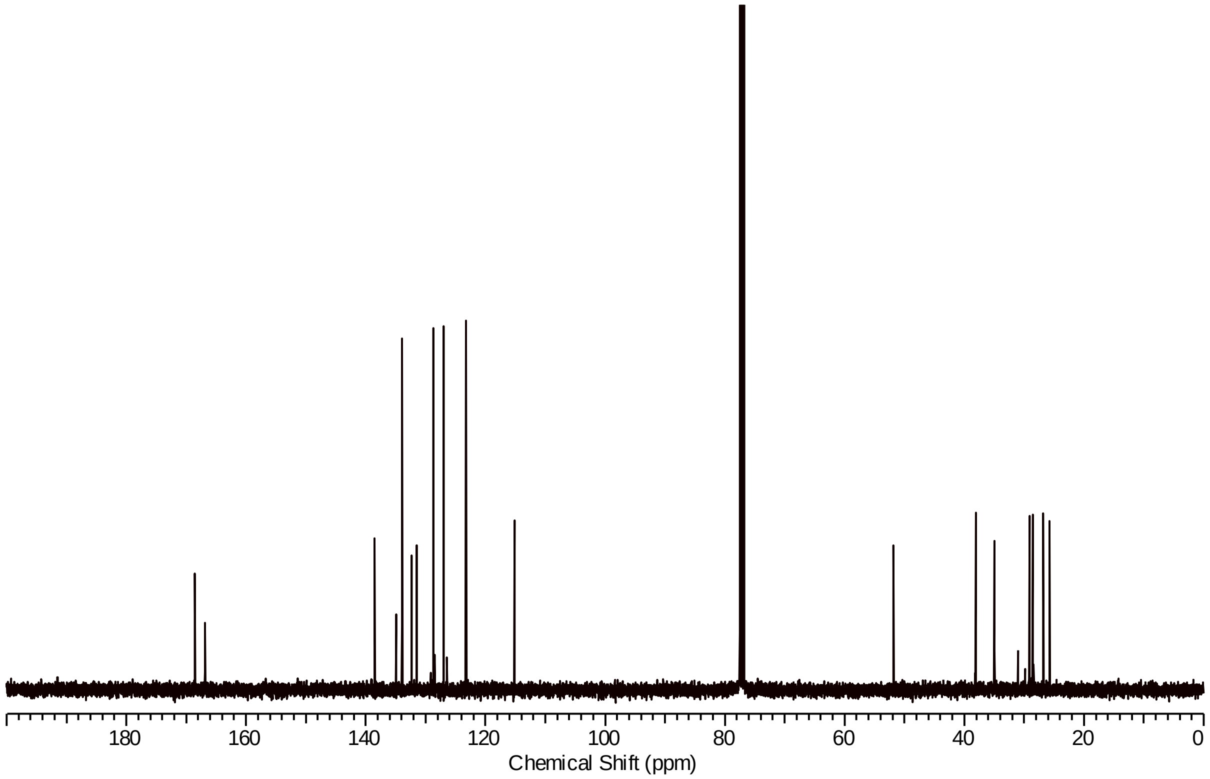

(*R*)-*N*-(5-phenoxypent-1-en-3-yl)benzamide **(1g)**

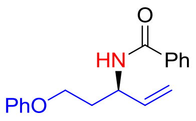

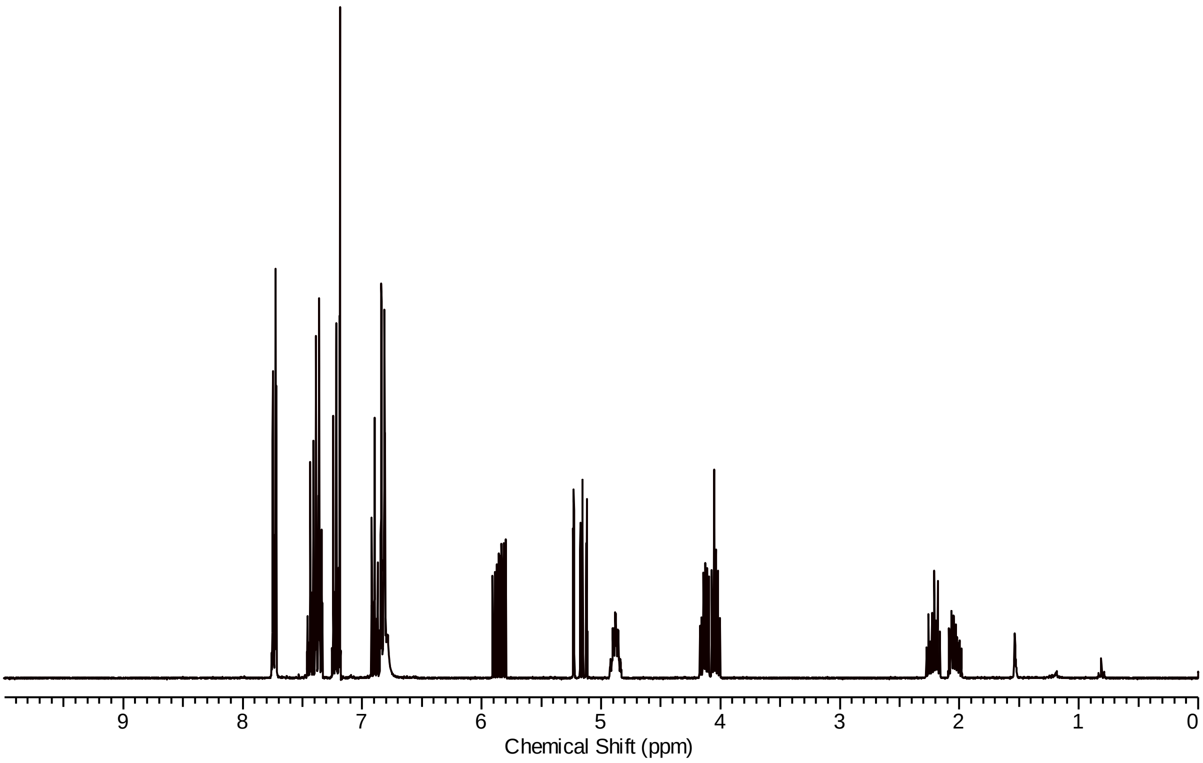

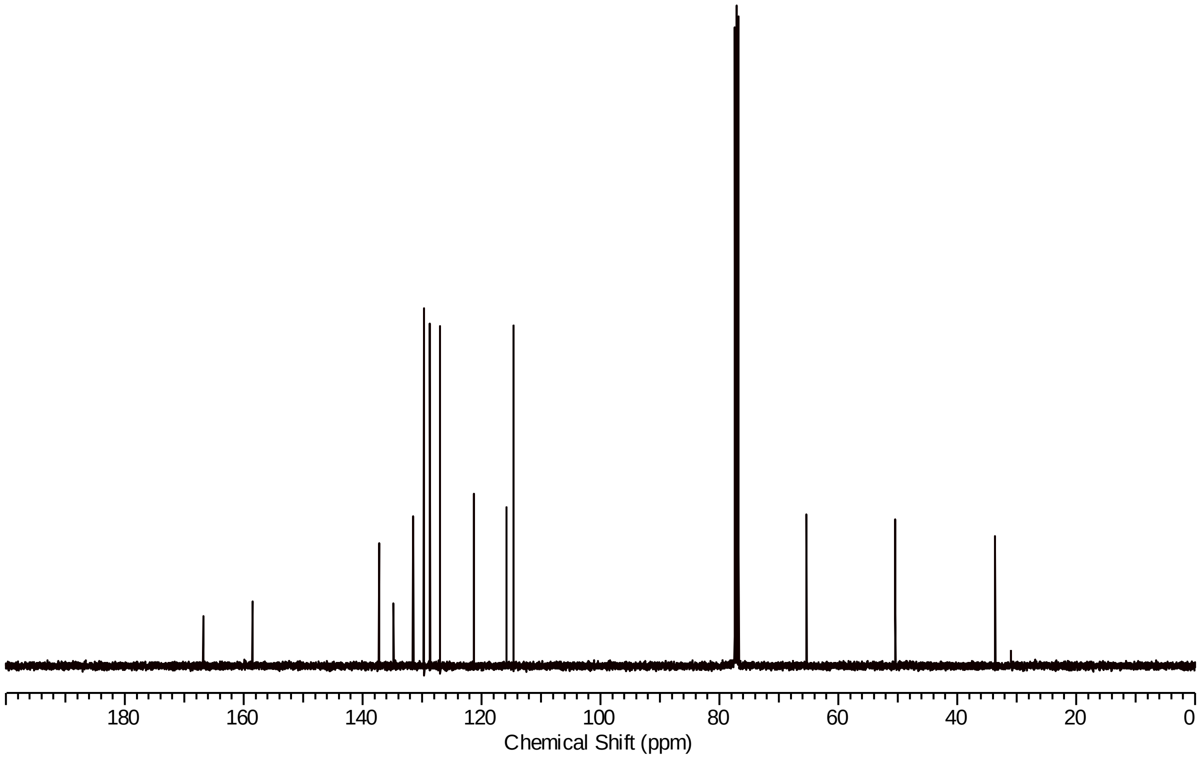

(*R*)-*N*-(6-(phenylthio)hex-1-en-3-yl)benzamide **(1h)**

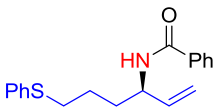

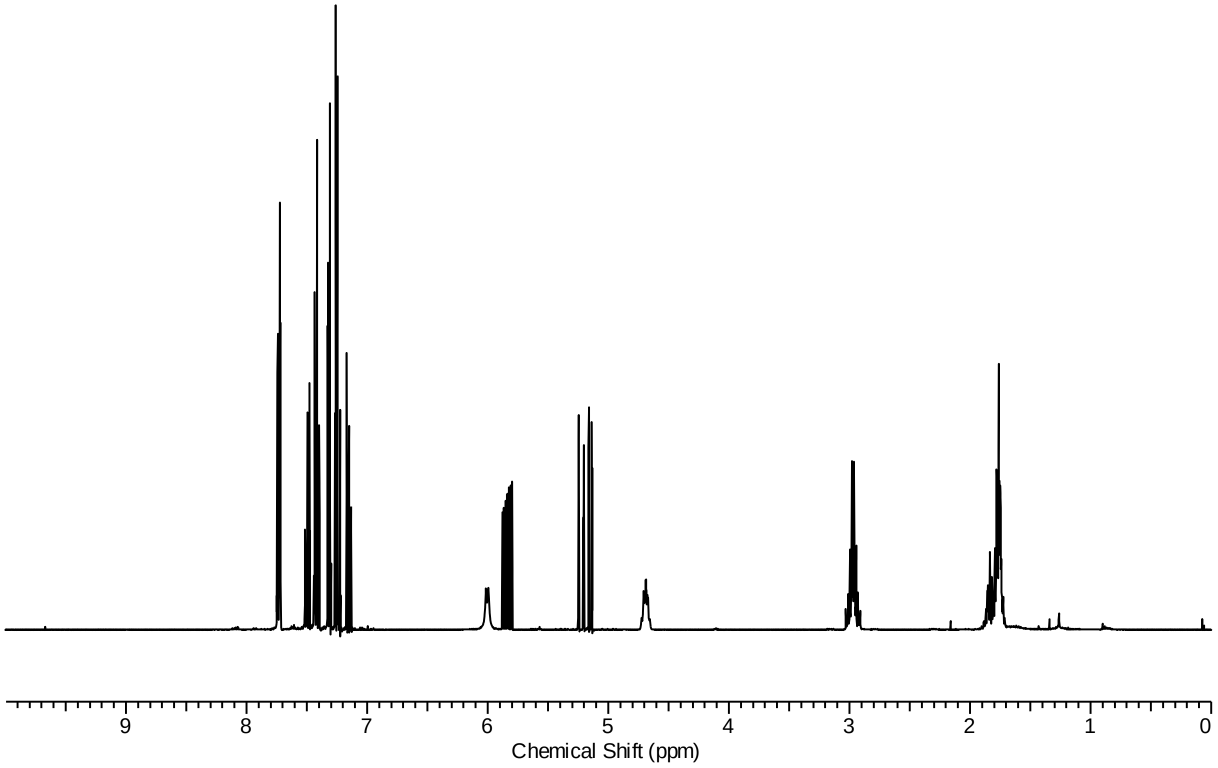

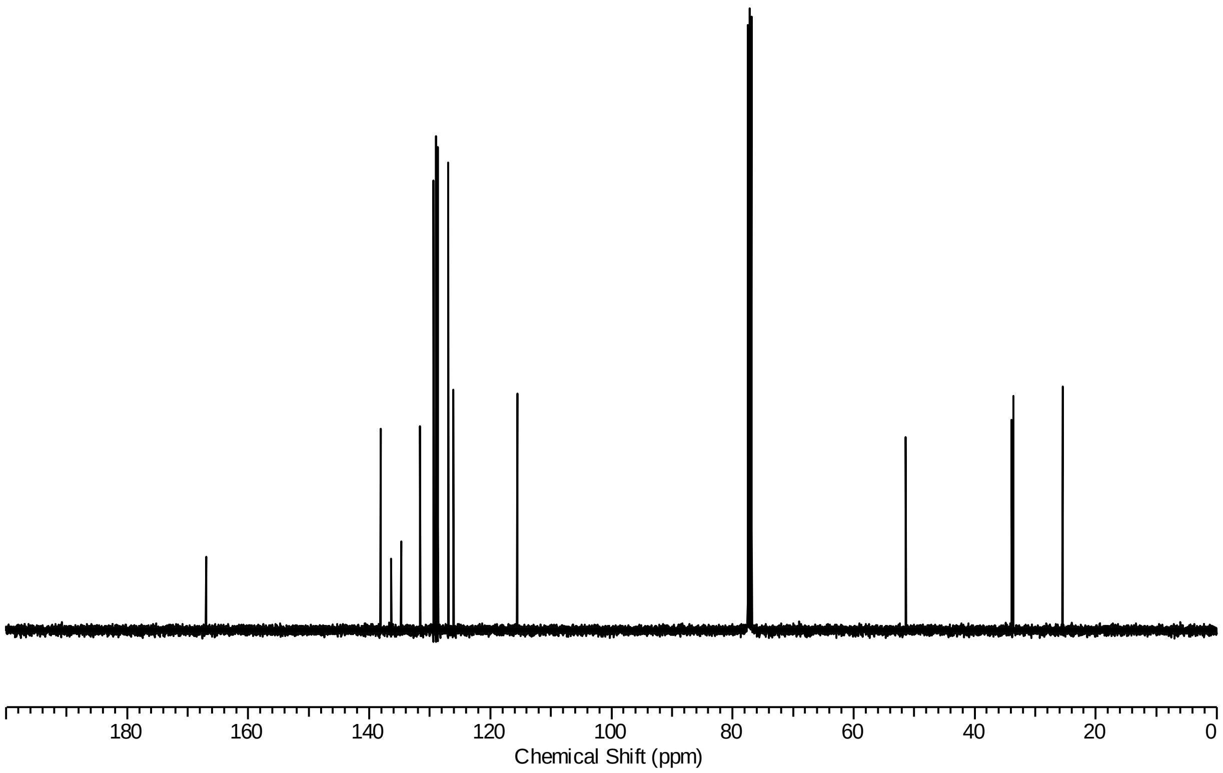

(*R*)-*N*-(6-(phenylsulfonyl)hex-1-en-3-yl)benzamide **(1i)**

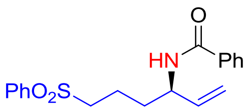

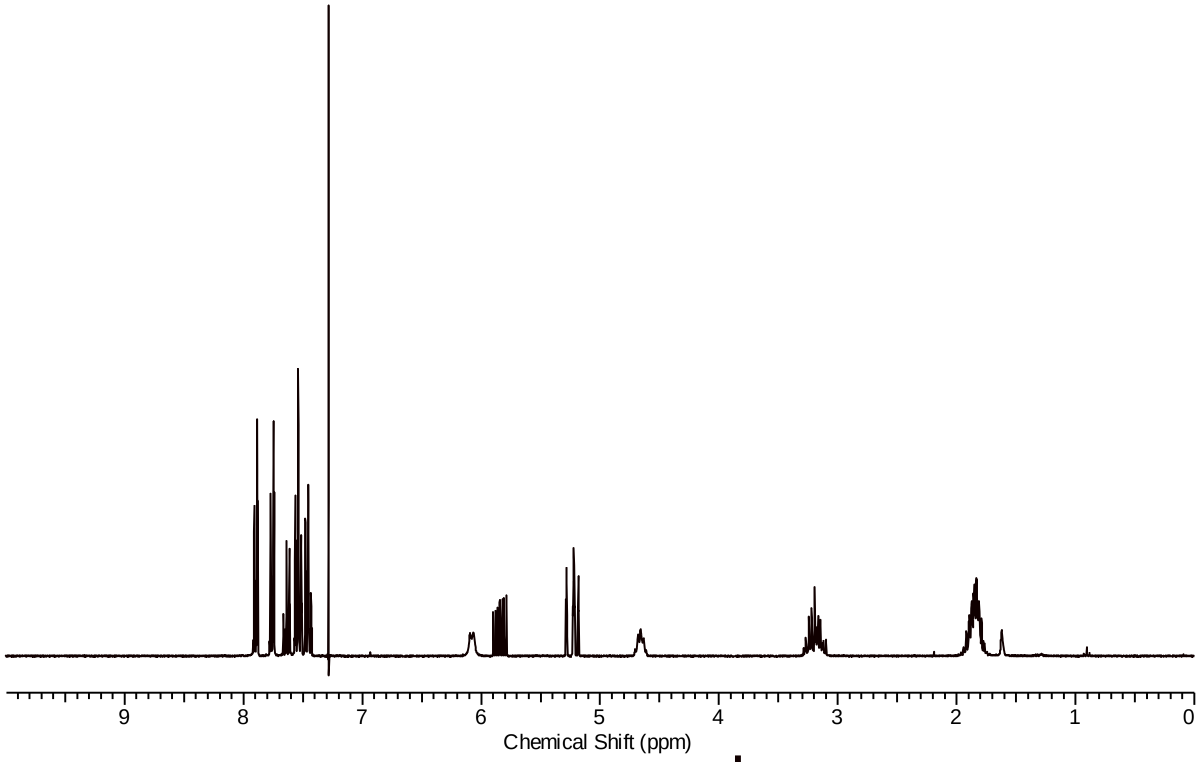

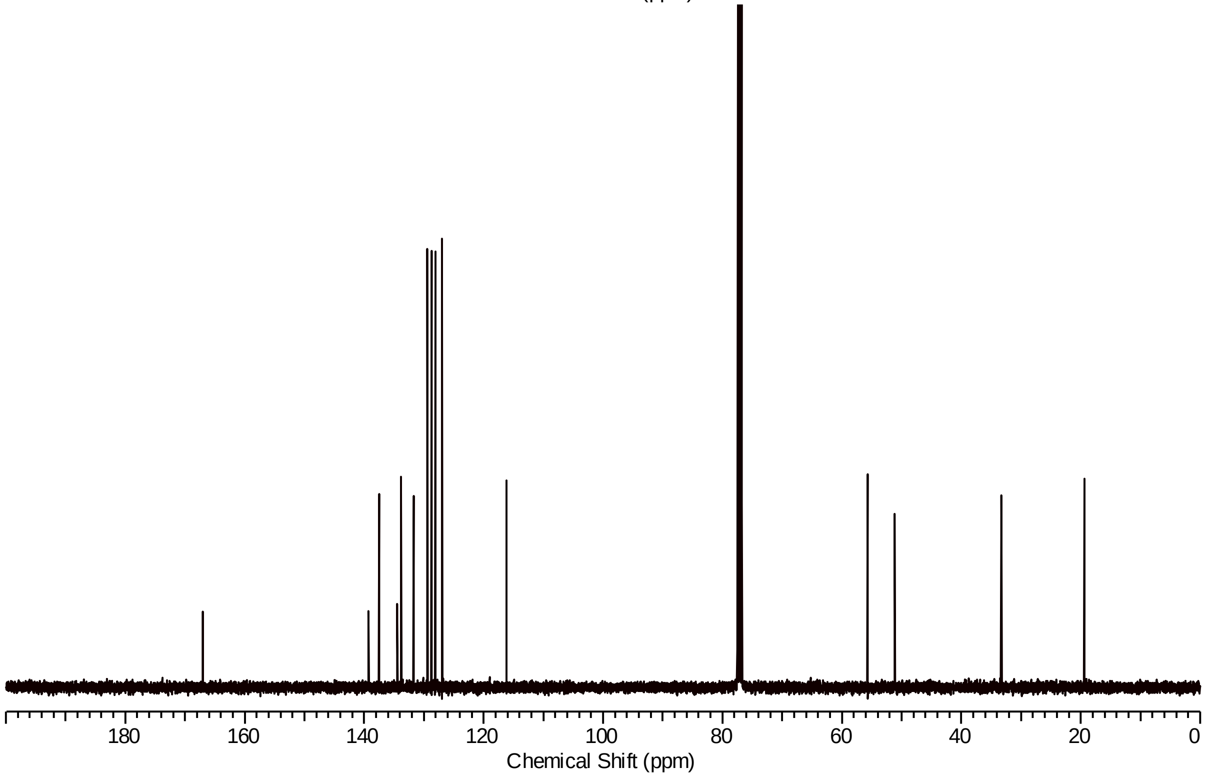

(*R*)-*tert*-butyl (6-phenylhex-1-en-3-yl)carbamate **(2a)**

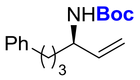

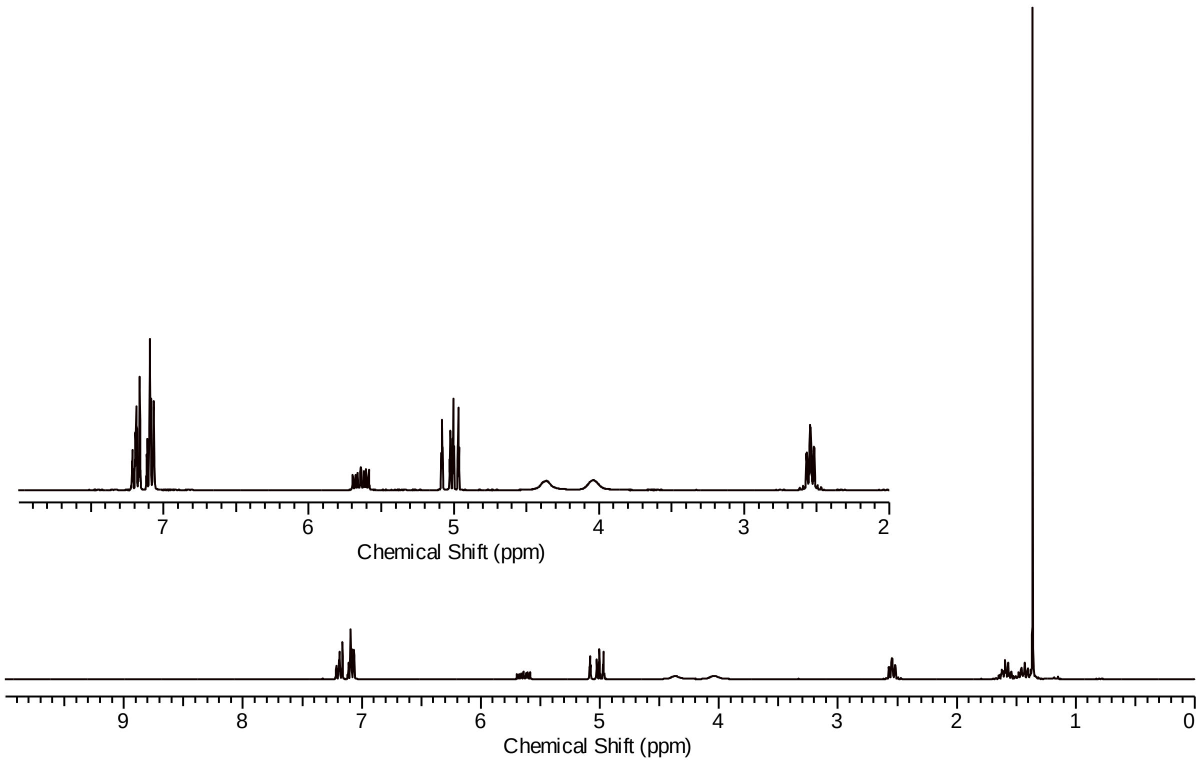

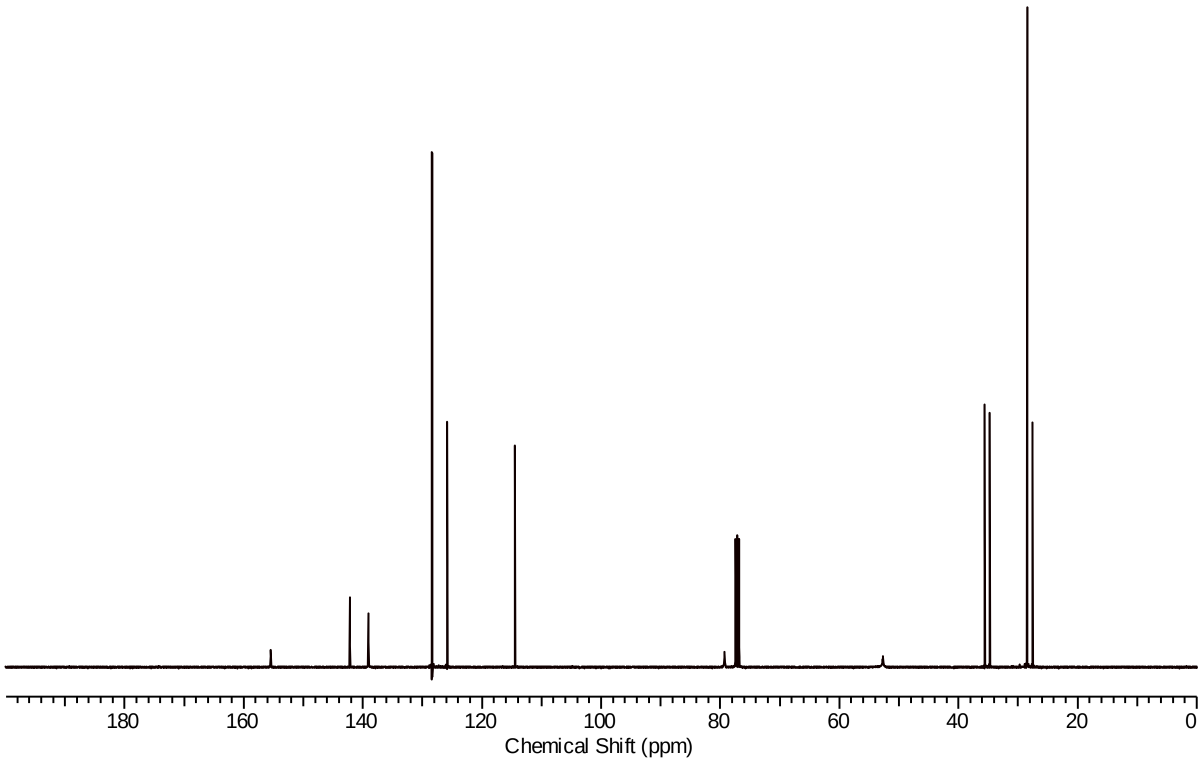

(*R*)-4-methyl-*N*-(6-phenylhex-1-en-3-yl)benzenesulfonamide **(2b)**

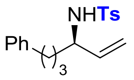

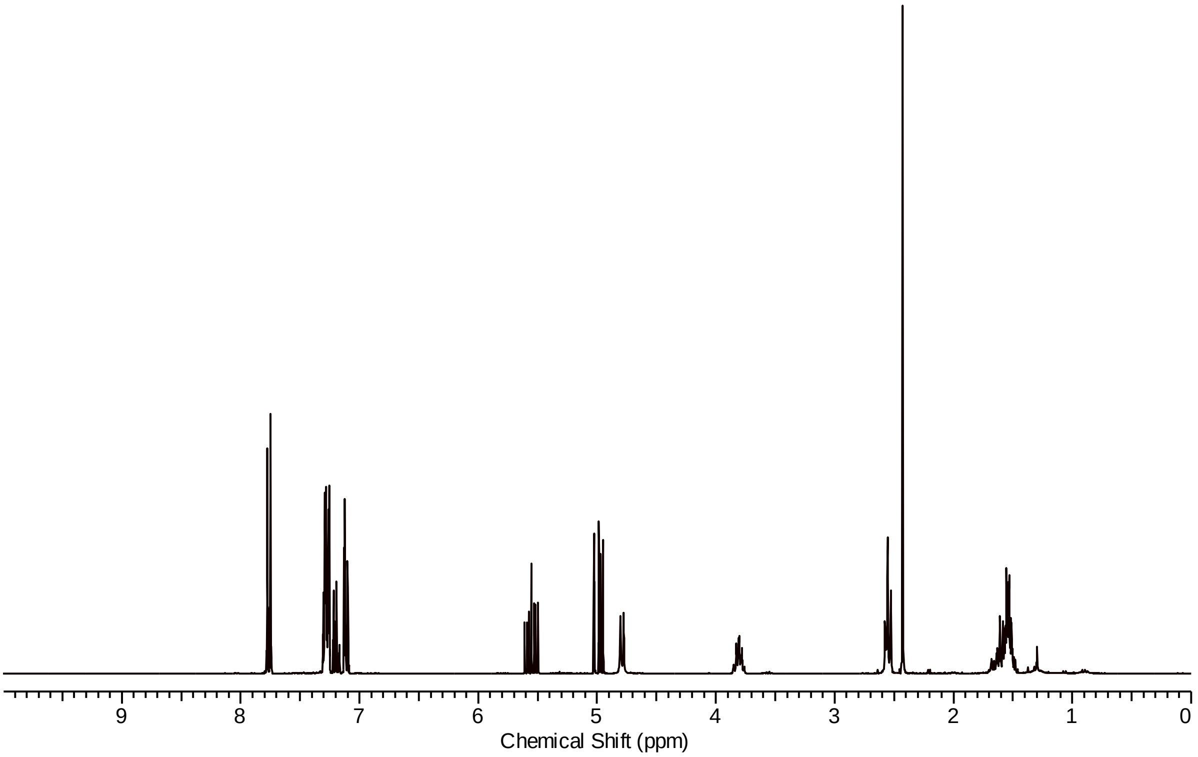

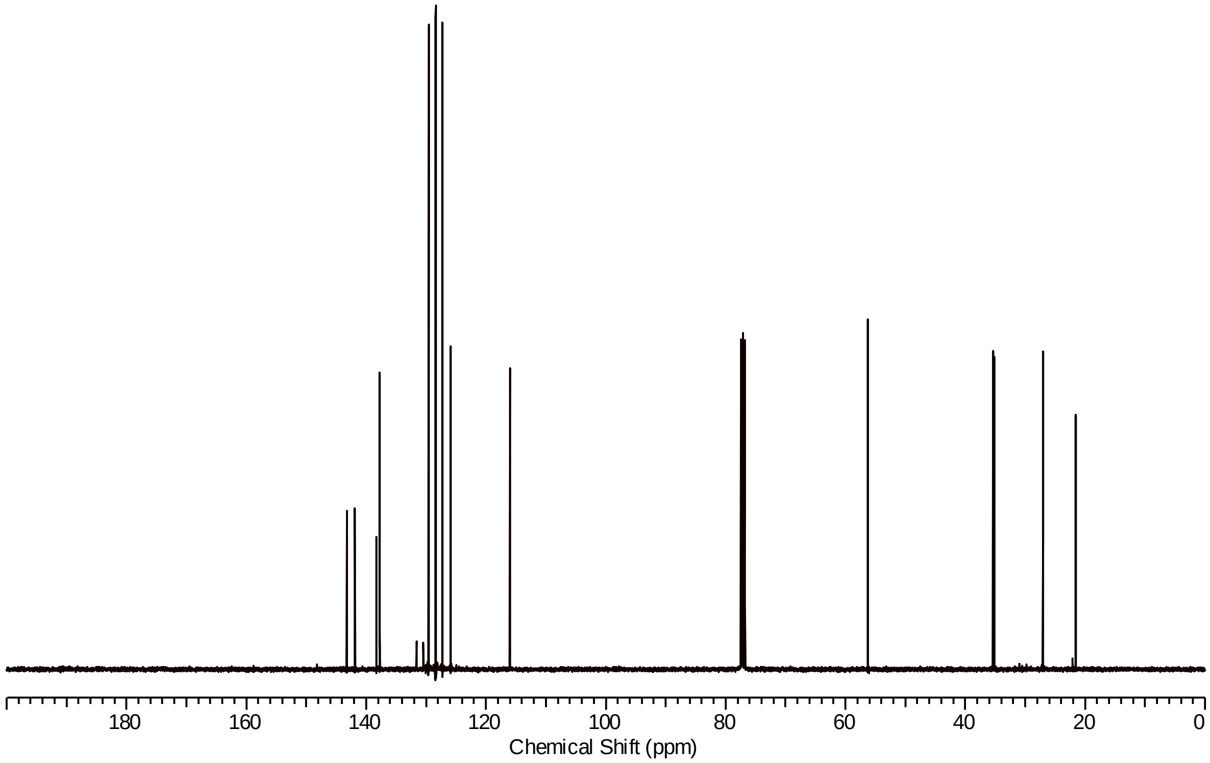

(*R*)-*N*-(6-phenylhex-1-en-3-yl)acrylamide **(2c)**

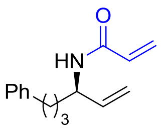

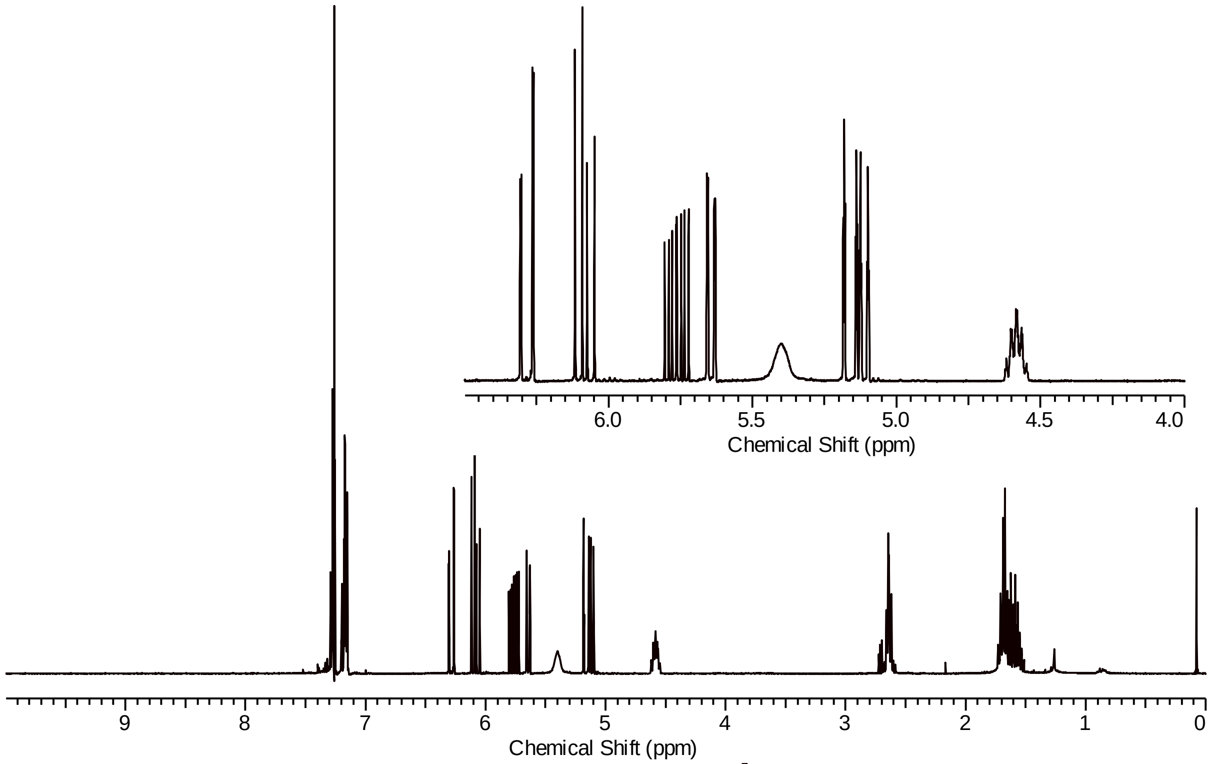

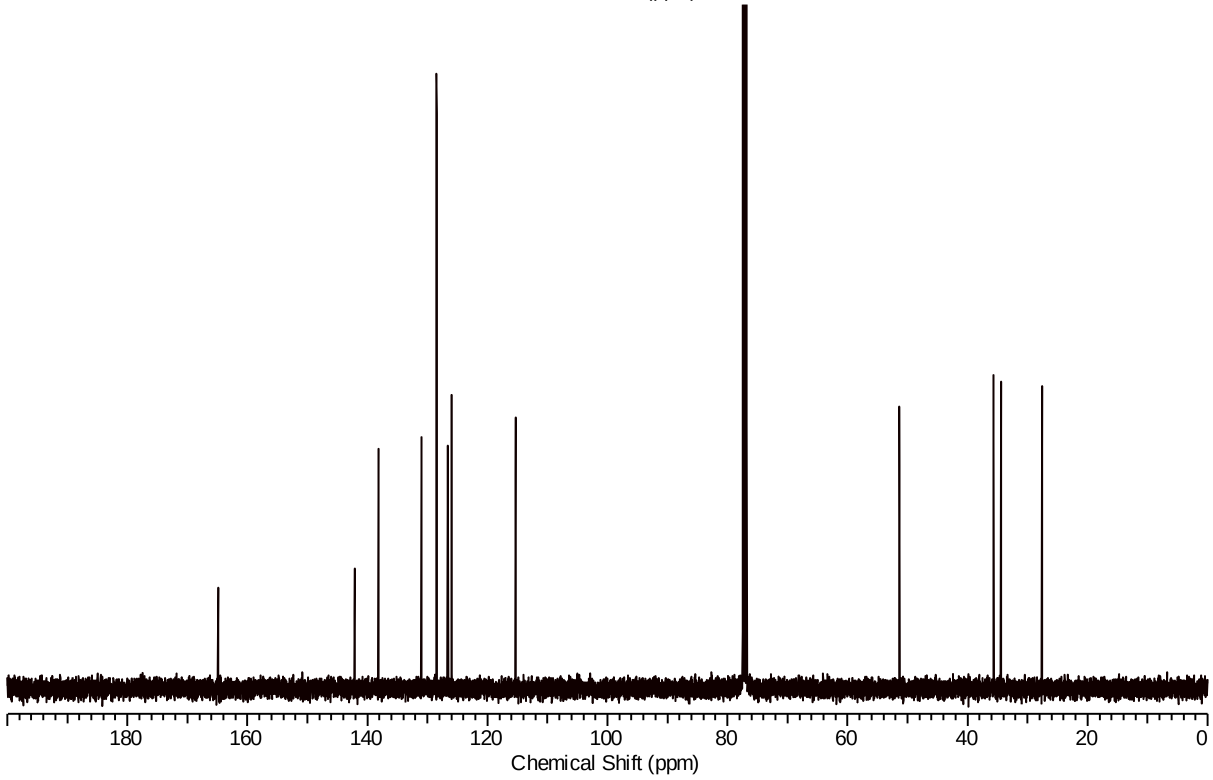

(*R*)-*N*-(6-phenylhex-1-en-3-yl)pent-4-enamide **(2d)**

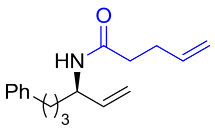

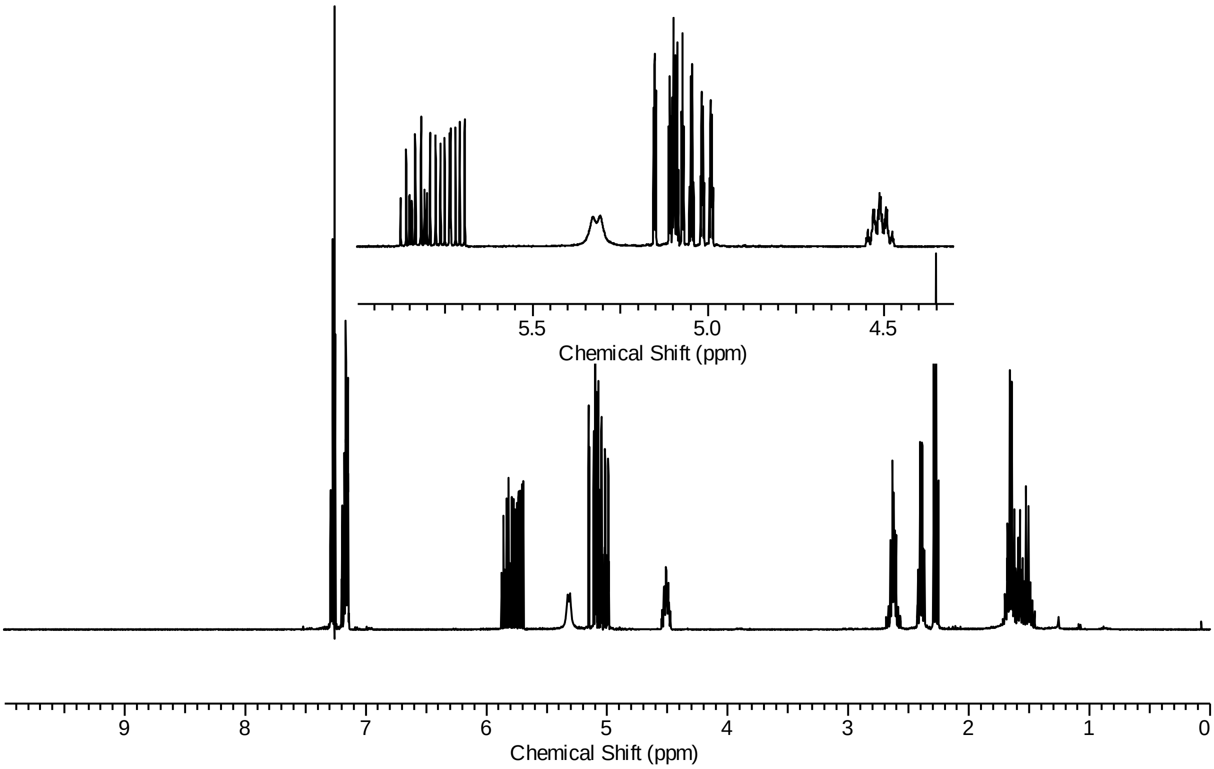

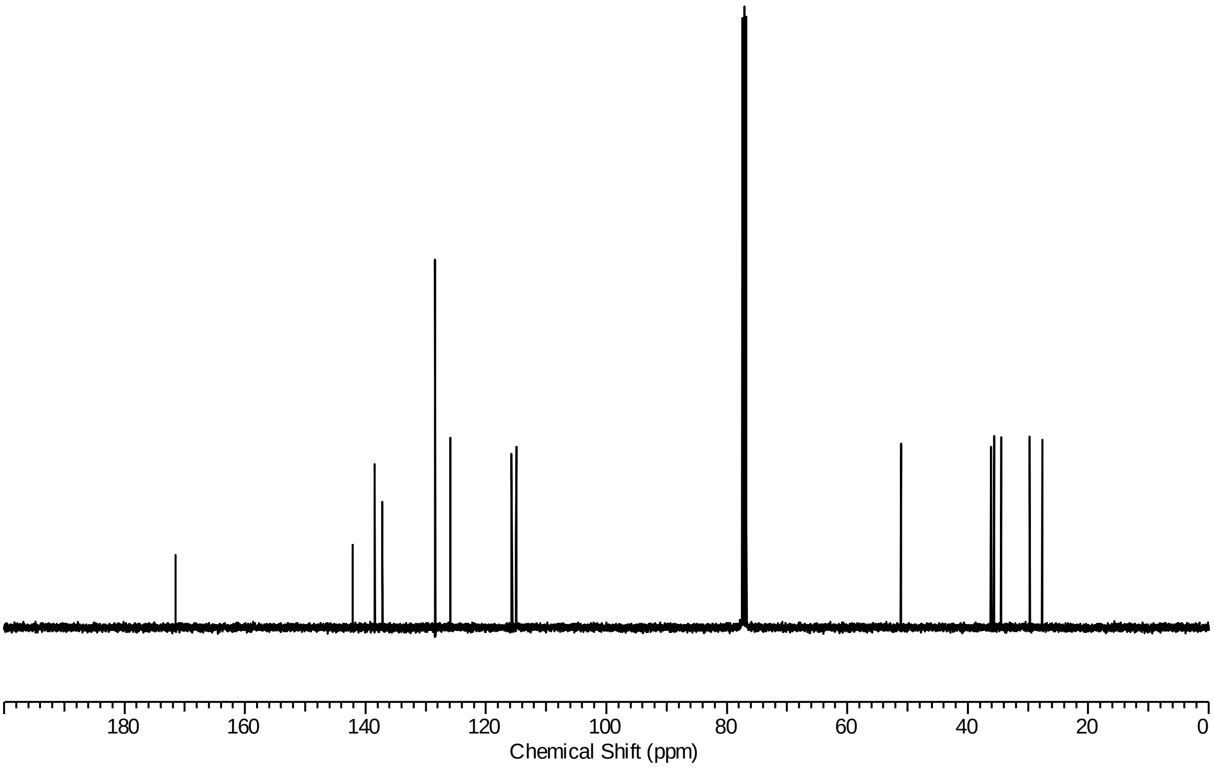

(*R*)-(1*R*,2*S*,5*R*)-2-isopropyl-5-methylcyclohexyl 4-benzamidohex-5-enoate **(3a)**

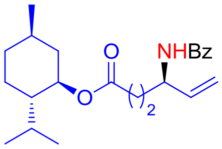

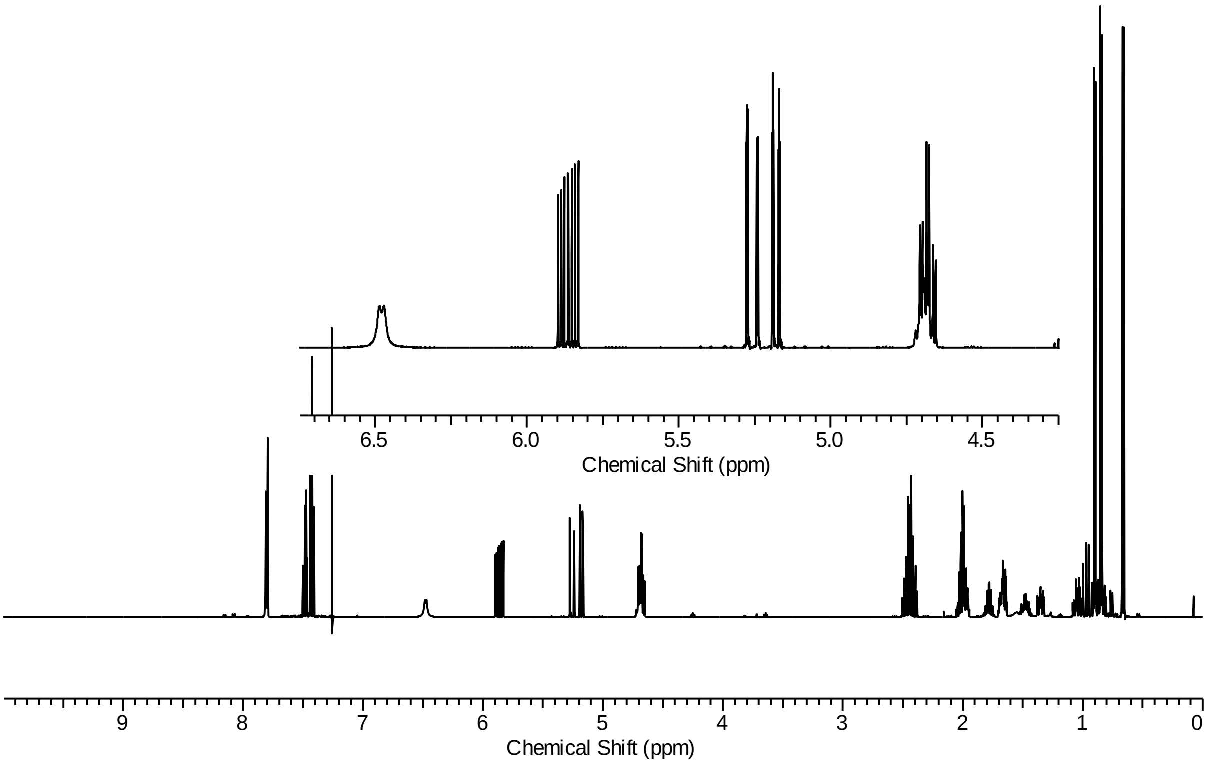

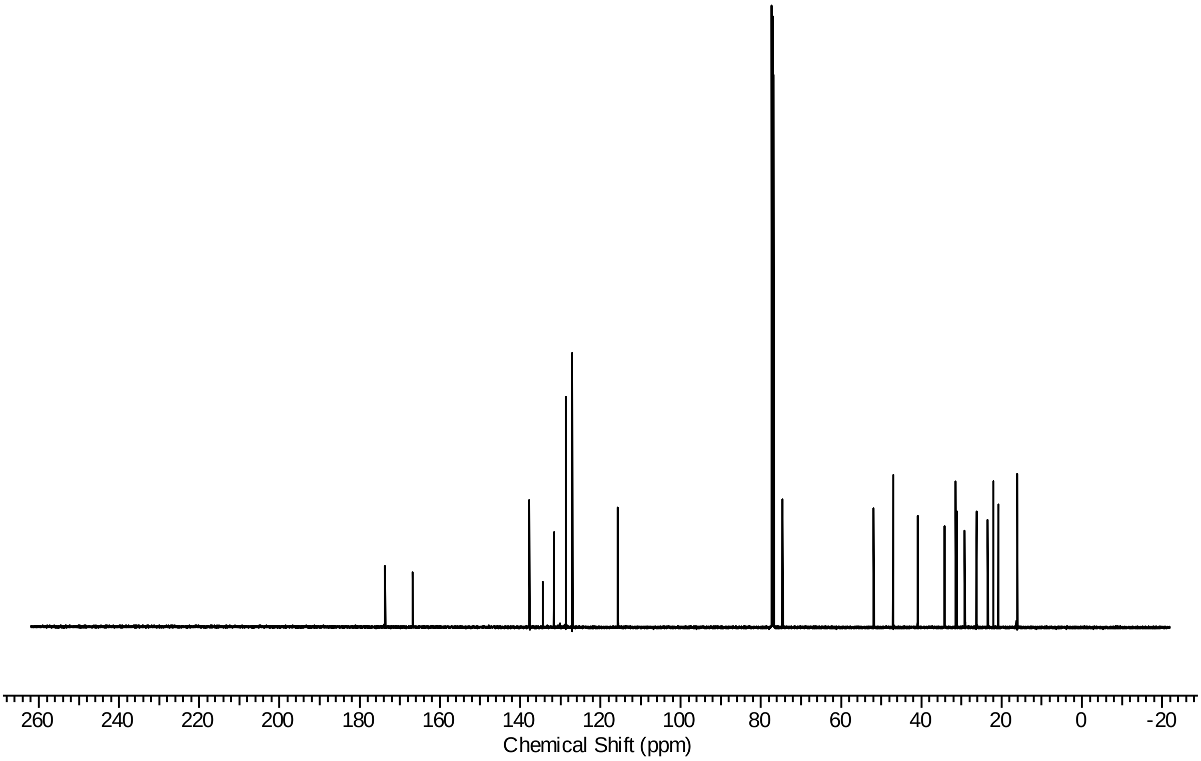

(*R*)-ethyl 4-benzamidohex-5-enoate (**3aee**)

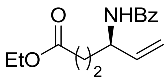

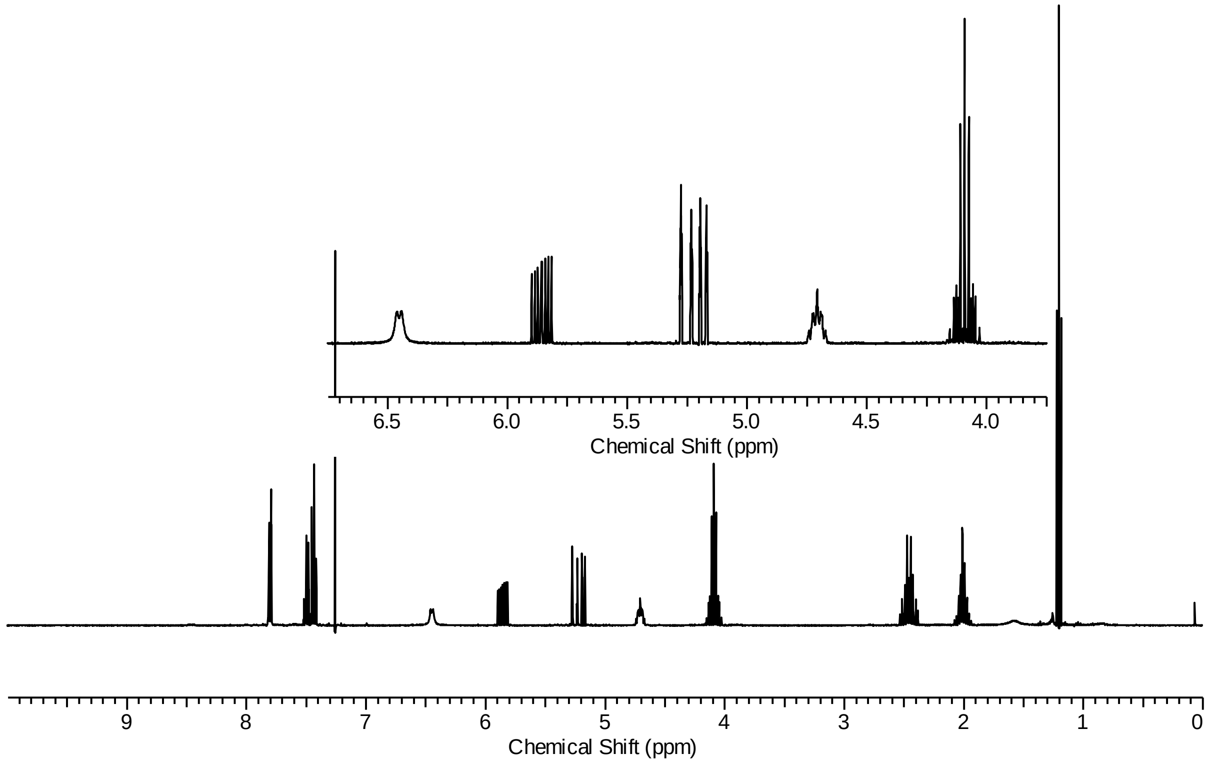

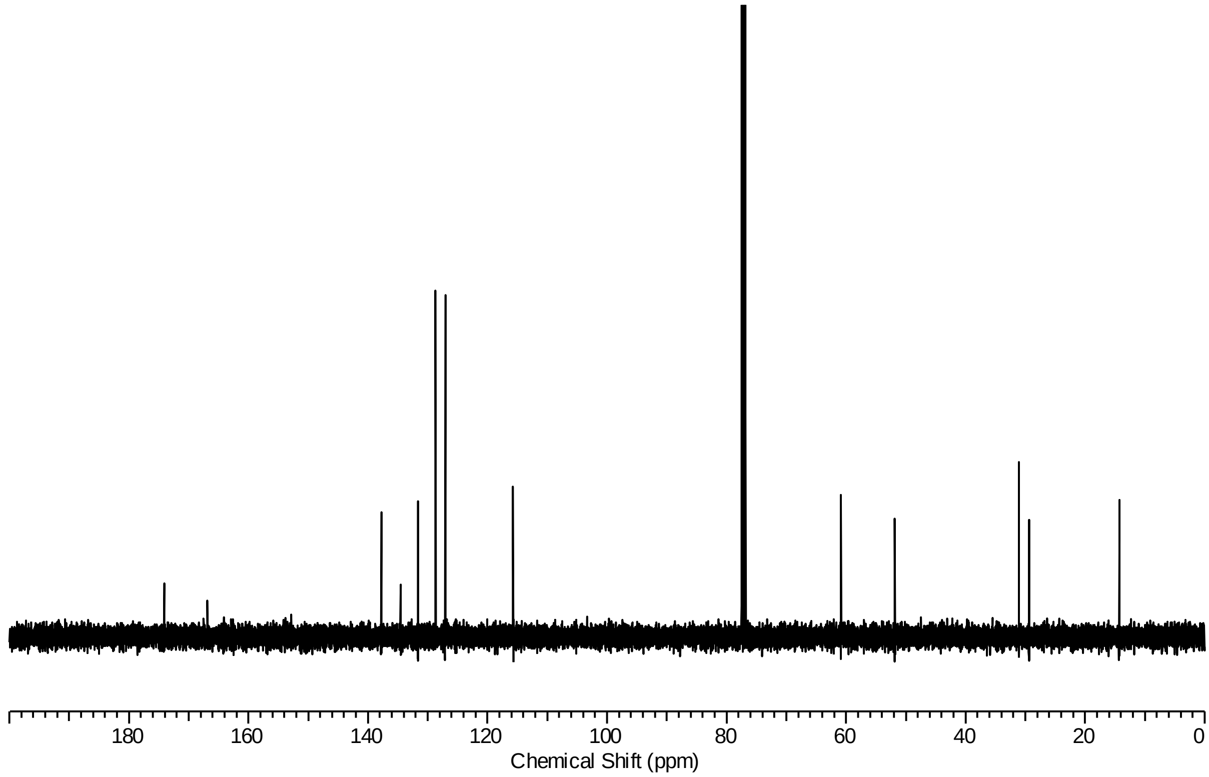

(*R*)-(1*R*,2*S*,5*R*)-2-isopropyl-5-methylcyclohexyl 5-benzamidohept-6-enoate **(3b)**

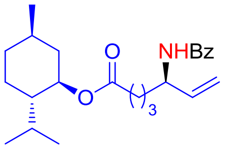

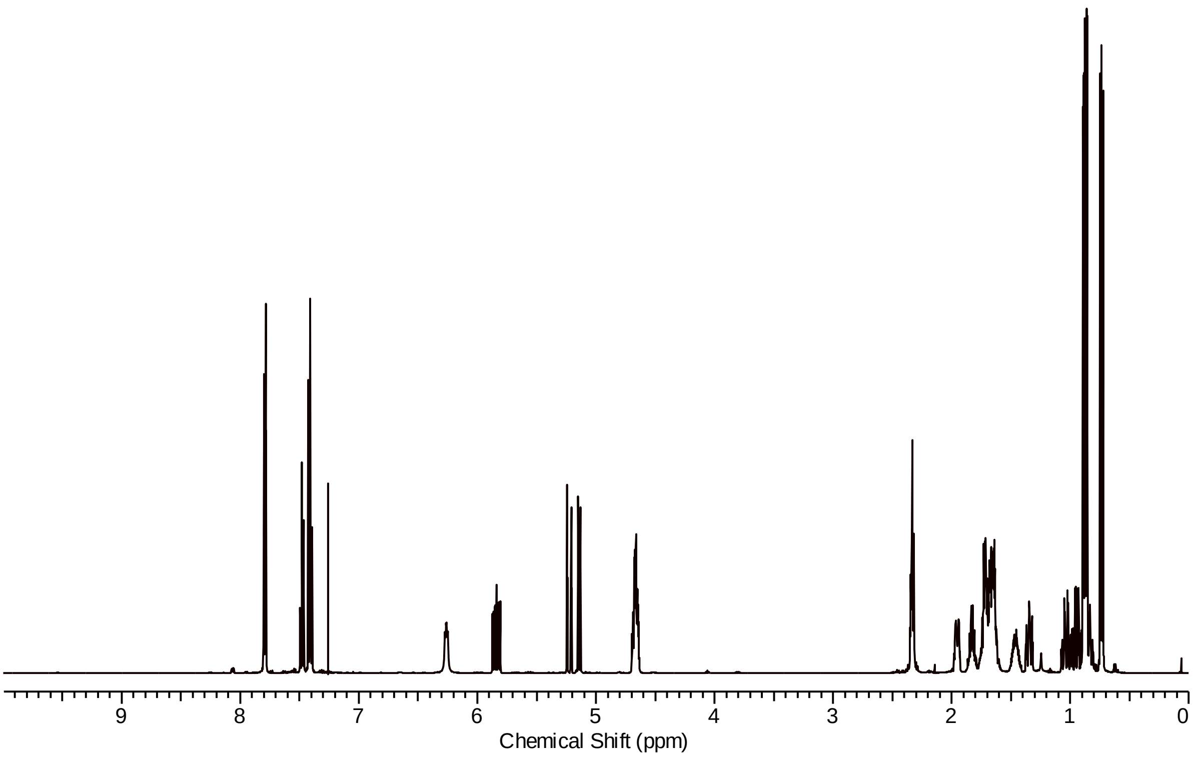

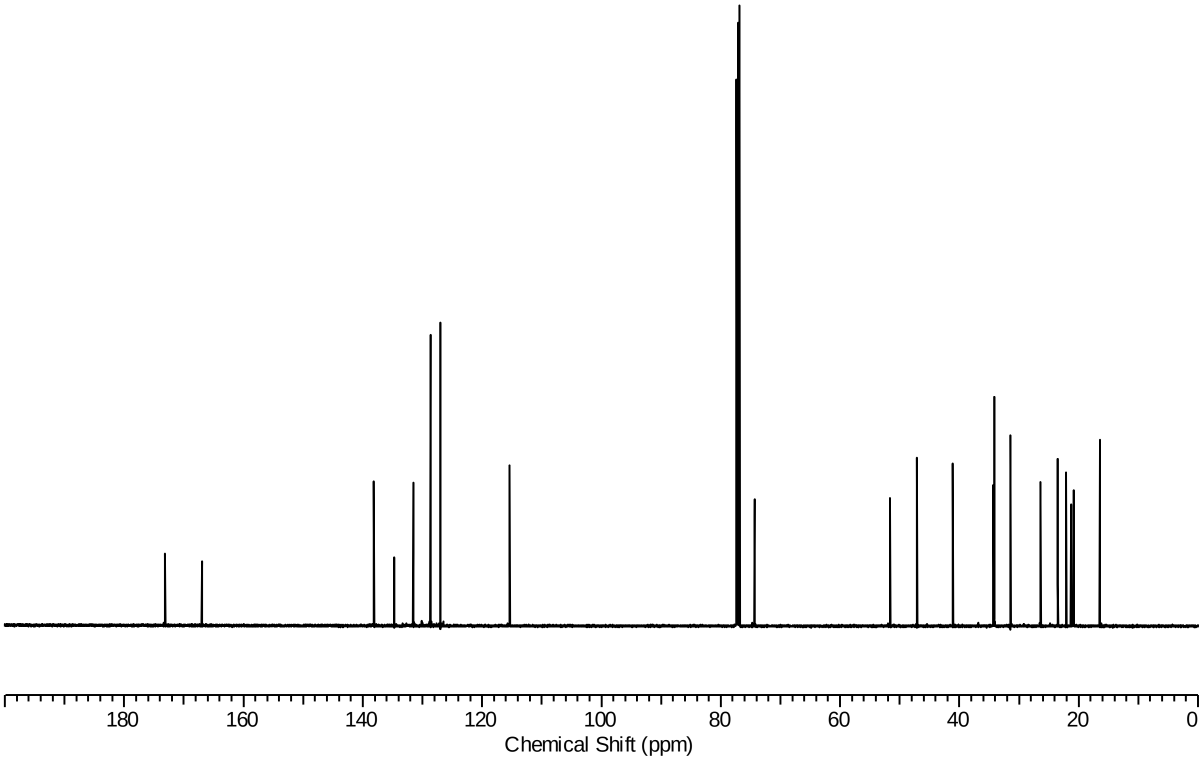

(*R*)-ethyl 5-benzamidohept-6-enoate **(3bee)**

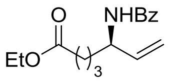

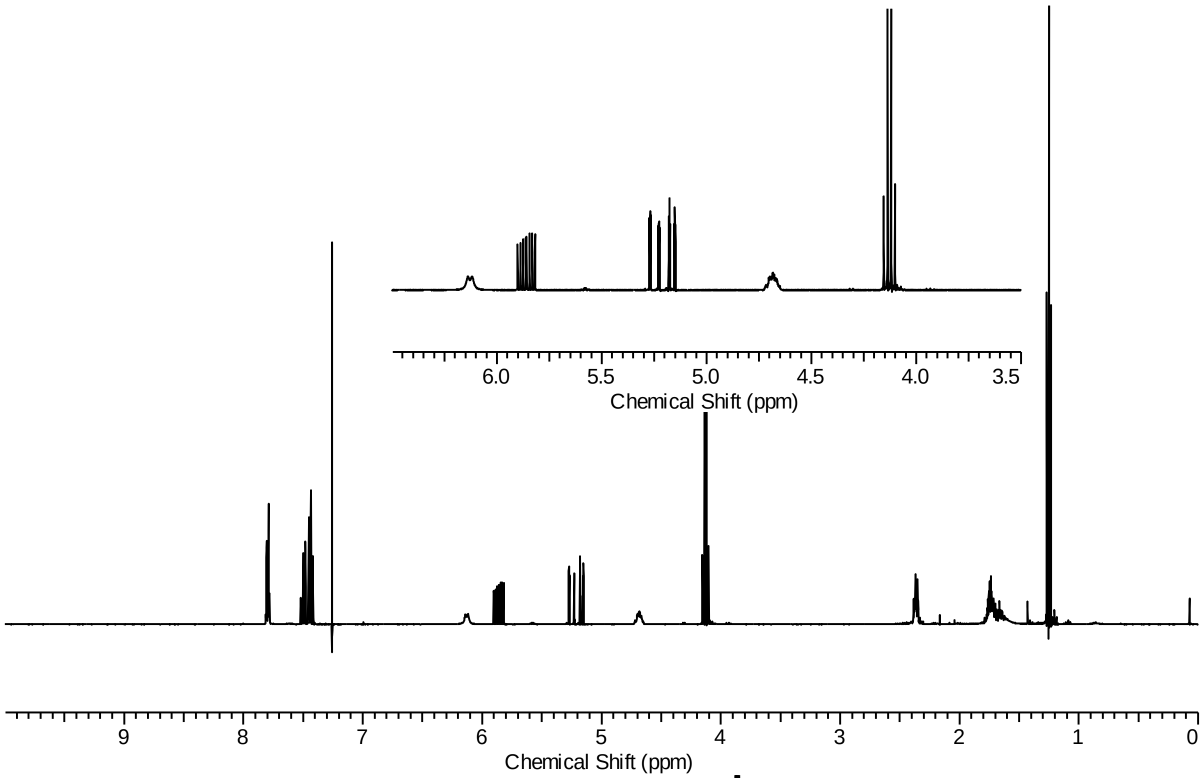

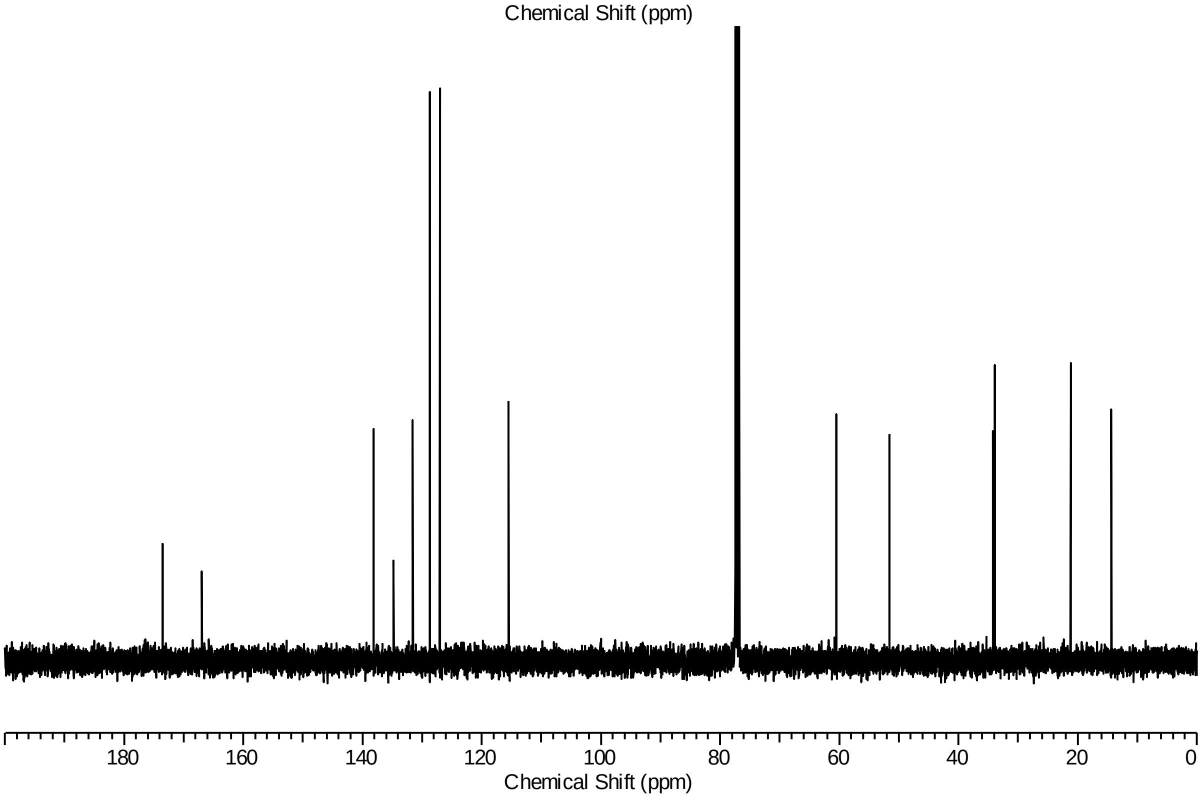

(*R*)-(1*S*,2*R*,4*S*)-1,7,7-trimethylbicyclo[2.2.1]heptan-2-yl 5-benzamidohept-6-enoate **(3c)**

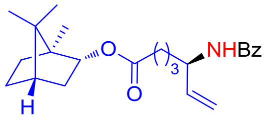

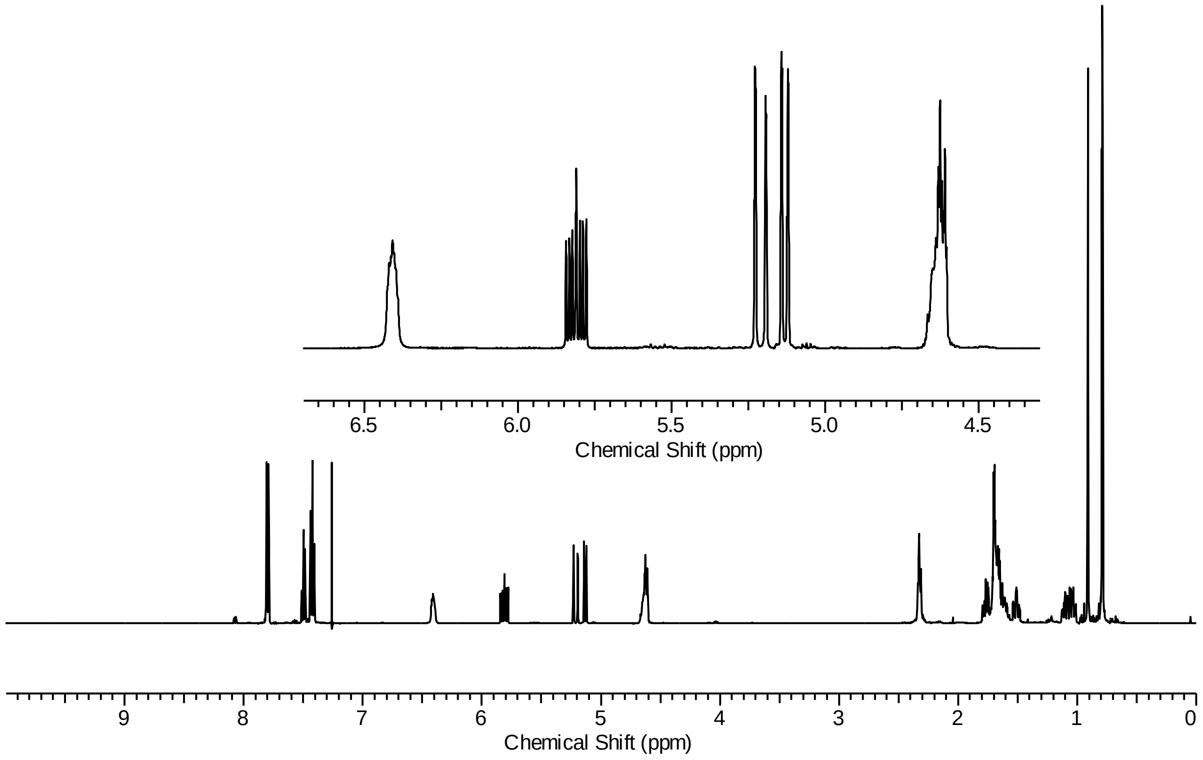

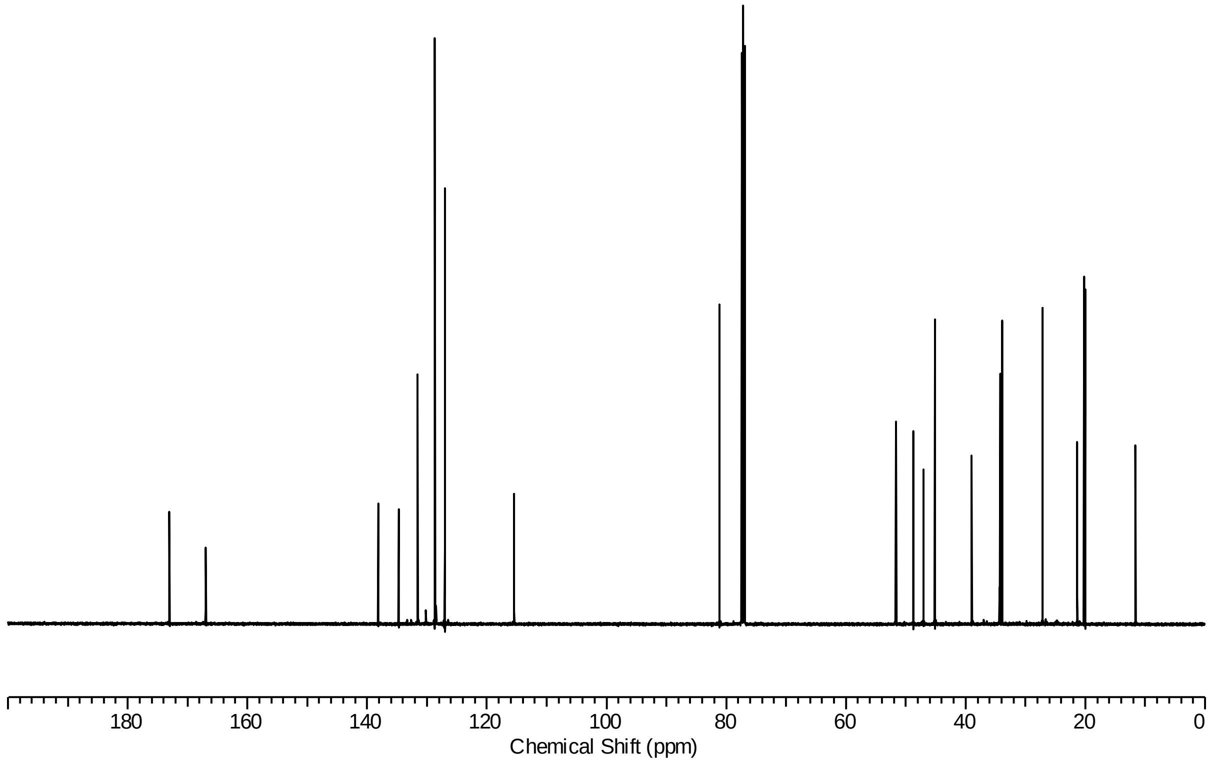

(*R*)-(3*R*,5*R*,8*S*,9*R*,10*R*,13*S*,14*R*,17*S*)-10,13-dimethyl-17-((*S*)-6-methylheptan-2-yl)hexadecahydro-1*H*-cyclopenta[a]phenanthren-3-yl 5-benzamidohept-6-enoate **(3d)**

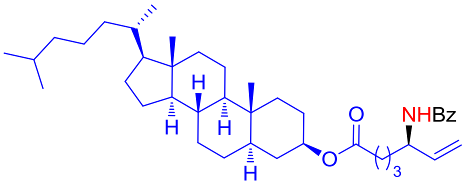

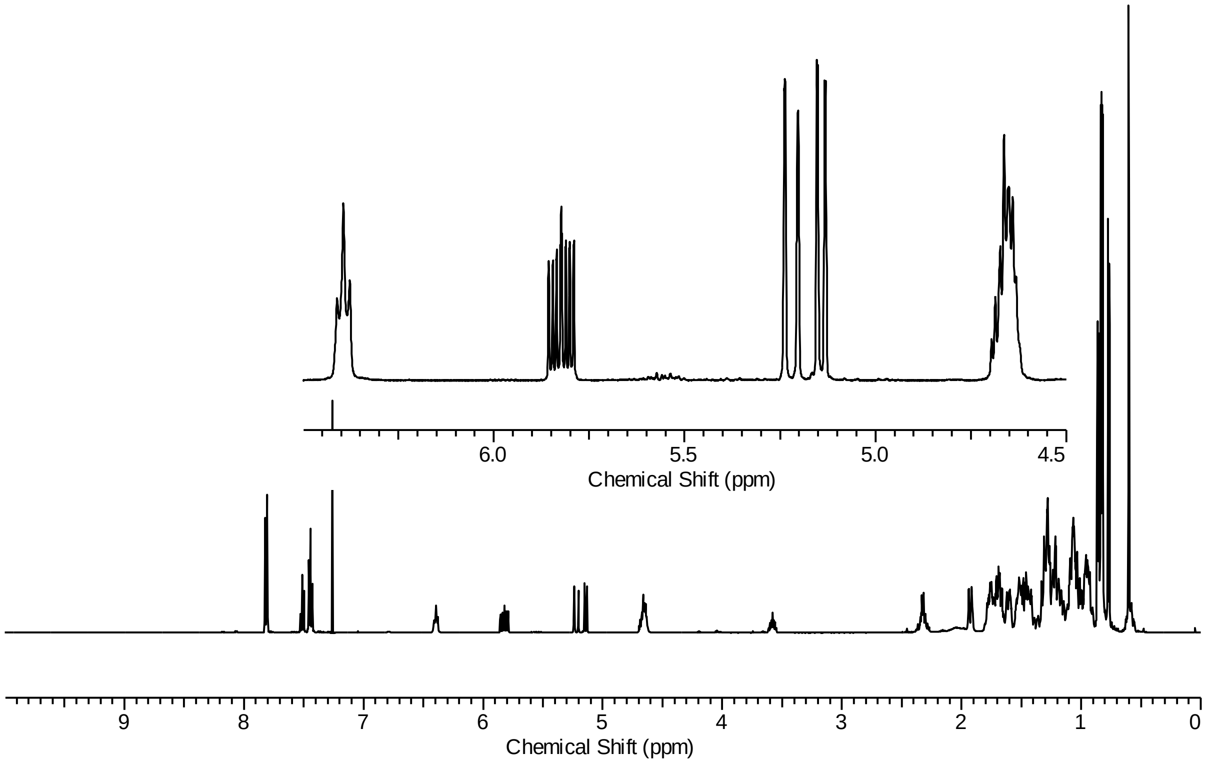

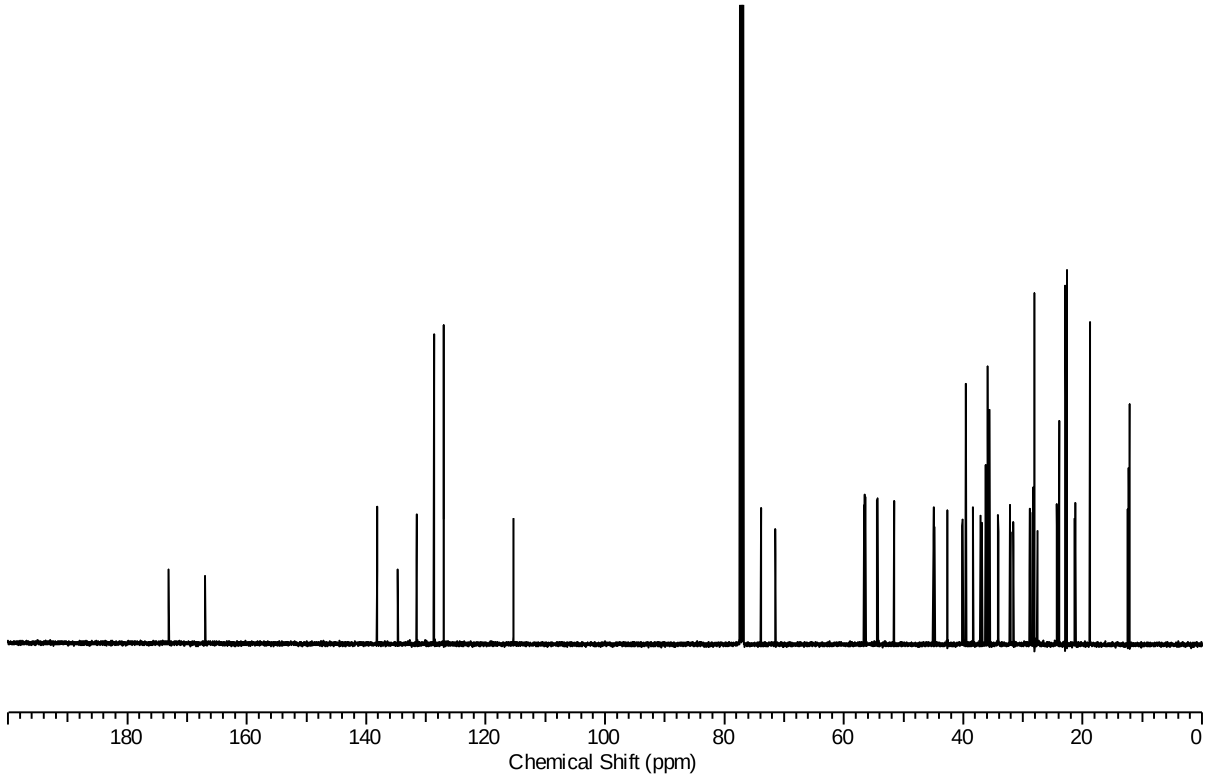

(*R*)-6-phenylhex-1-en-3-aminium chloride (**4**)

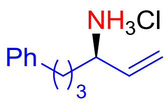

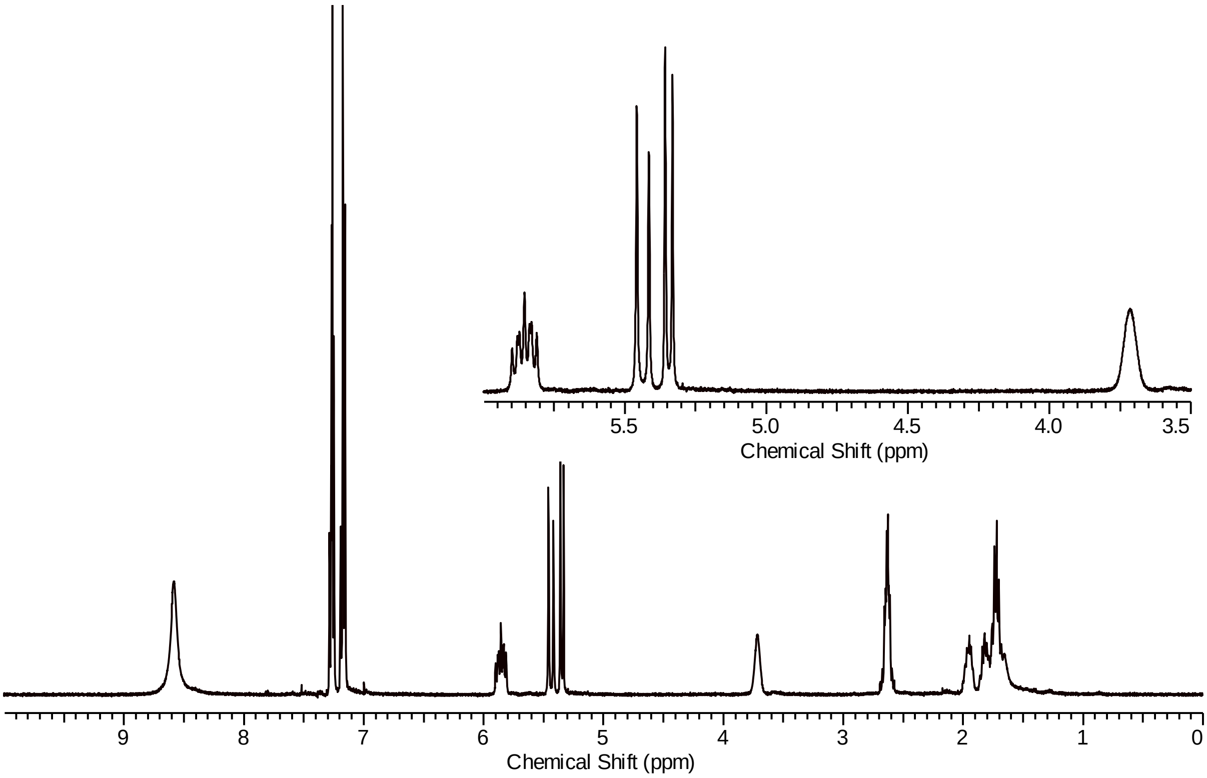

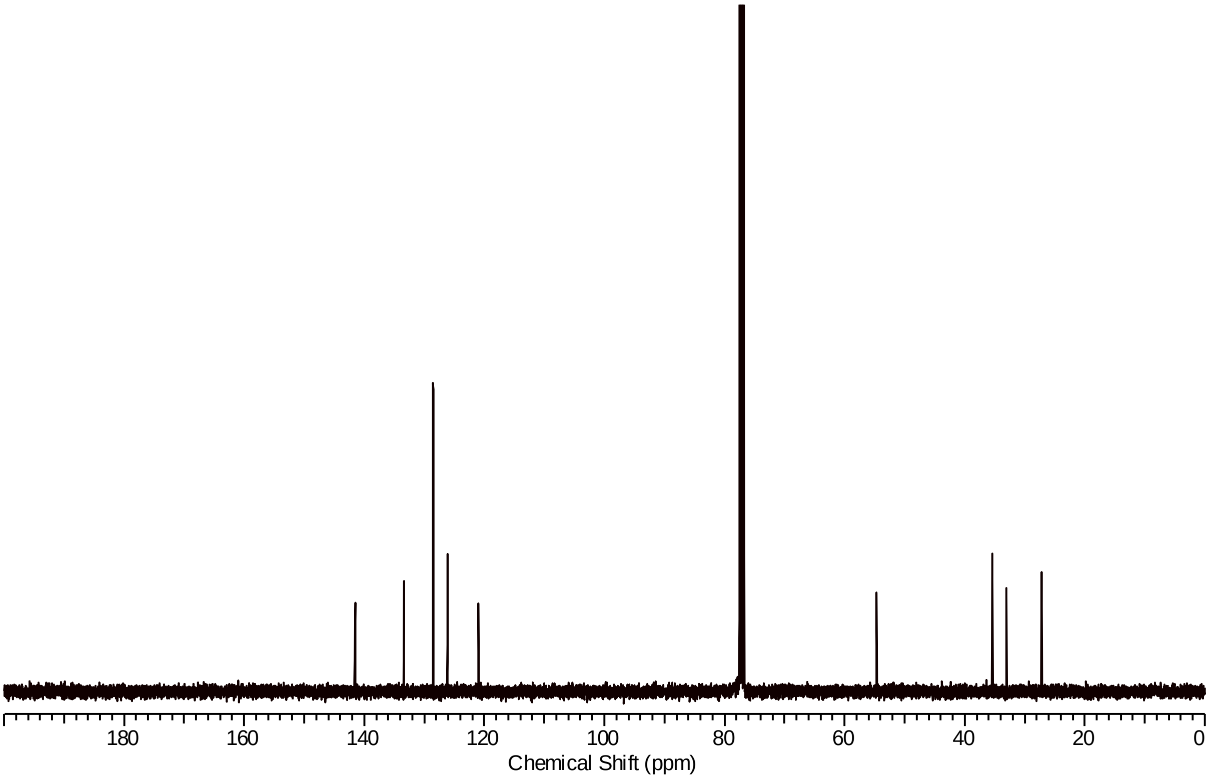

(*R*)-*tert*-butyl (1-hydroxy-5-phenylpentan-2-yl)carbamate (**5a**)

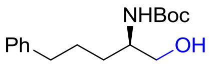

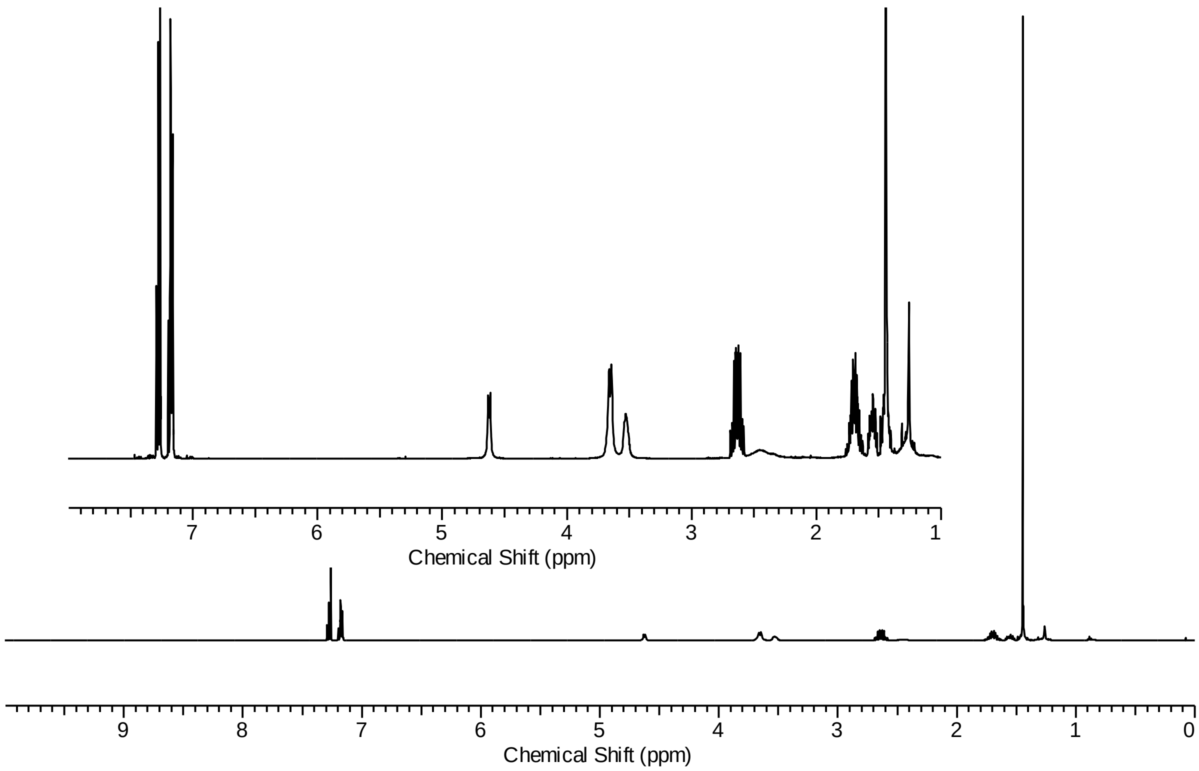

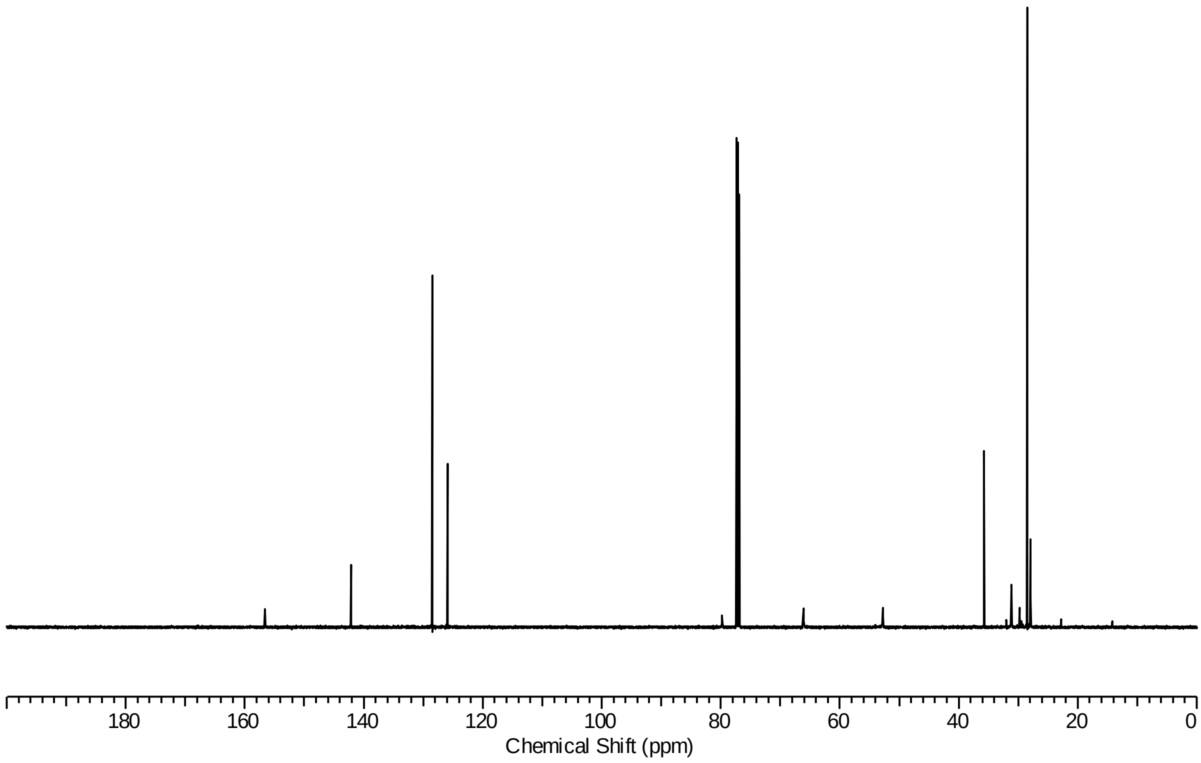

(*R*)-*tert*-butyl (1-oxo-5-phenylpentan-2-yl)carbamate (**5b**)

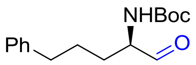

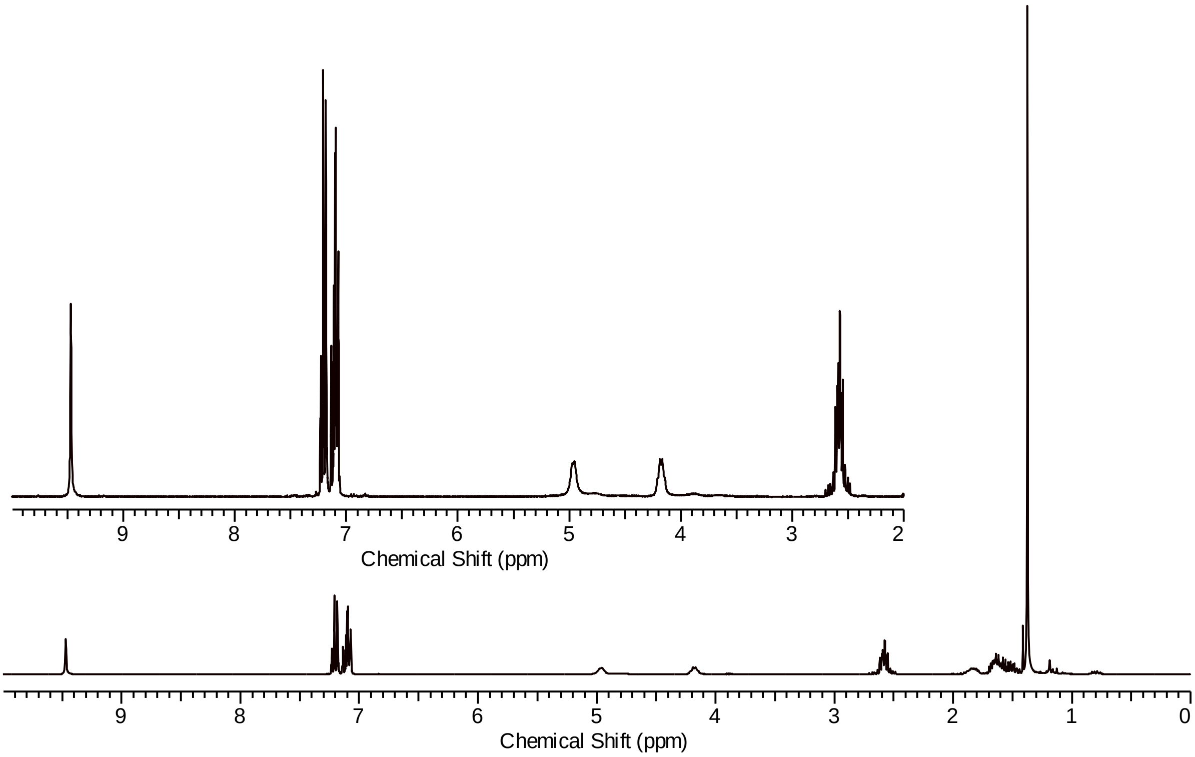

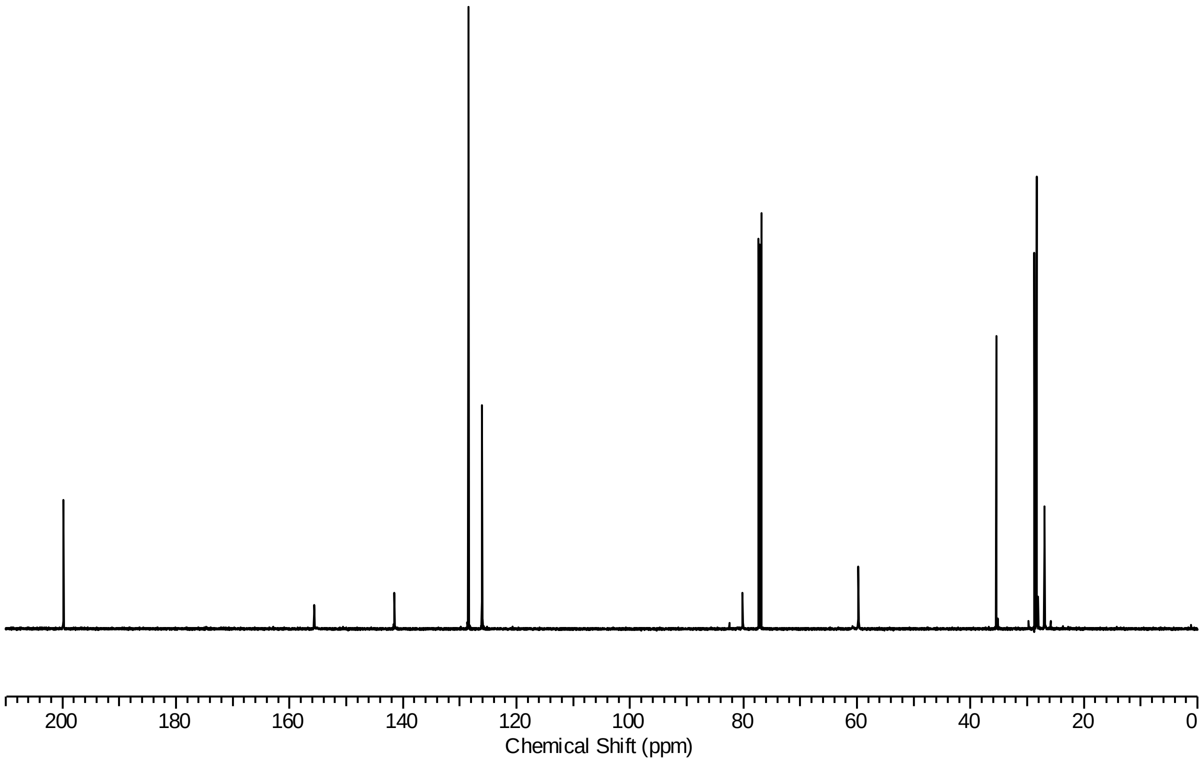

(*R*)-2-((tert-butoxycarbonyl)amino)-5-phenylpentanoic acid (**5c**)

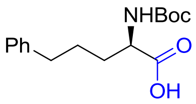

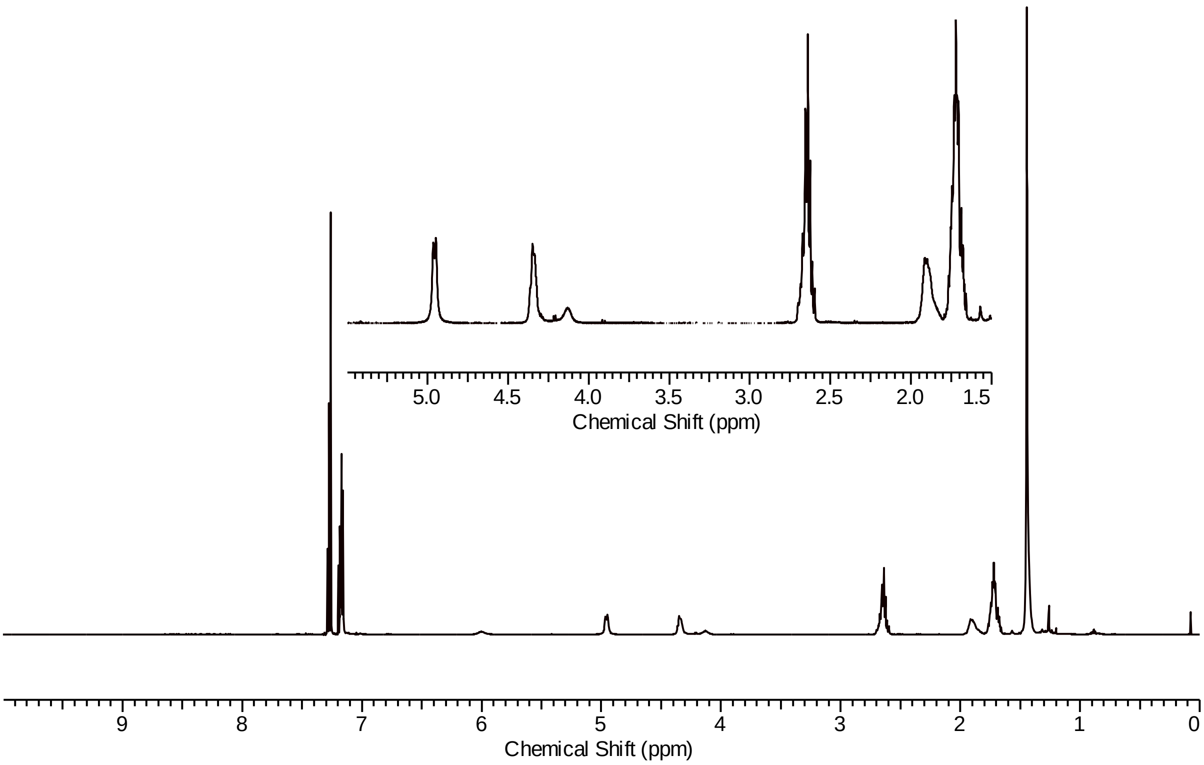

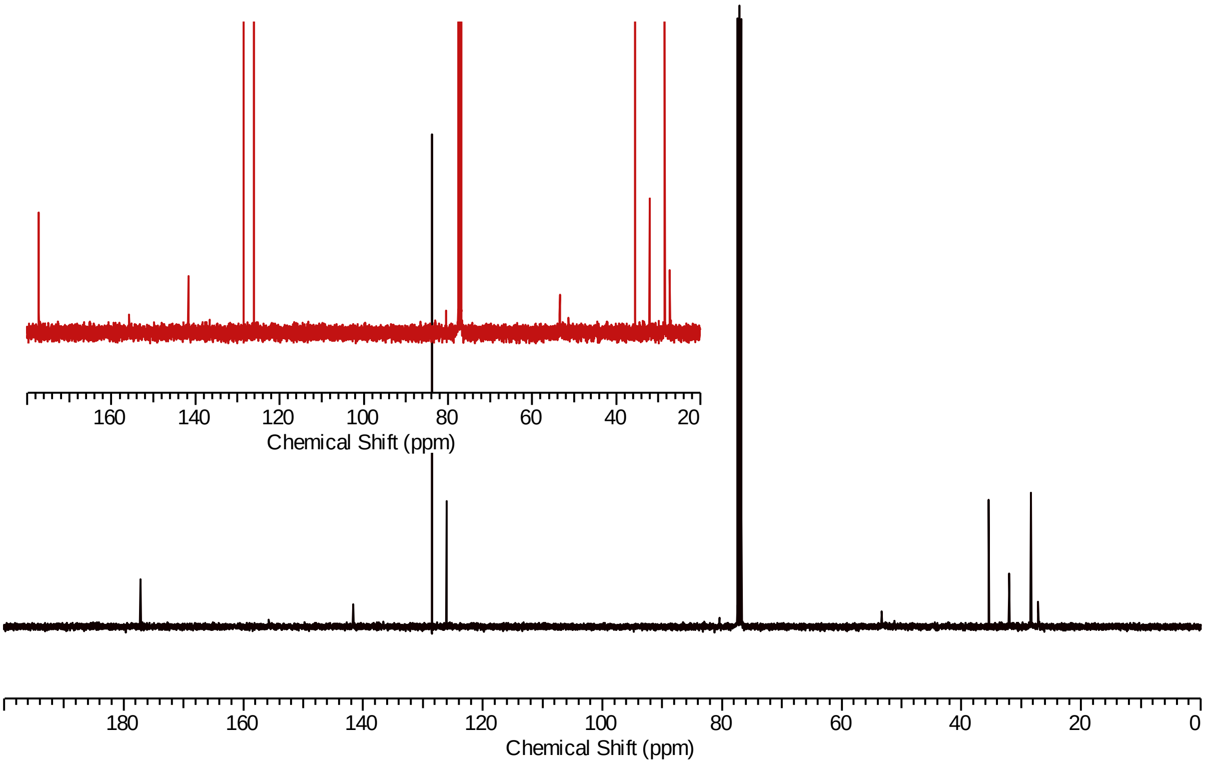

(*R*)-*tert*-butyl (1-hydroxy-6-phenylhexan-3-yl)carbamate (**5d**)

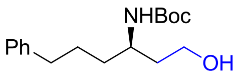

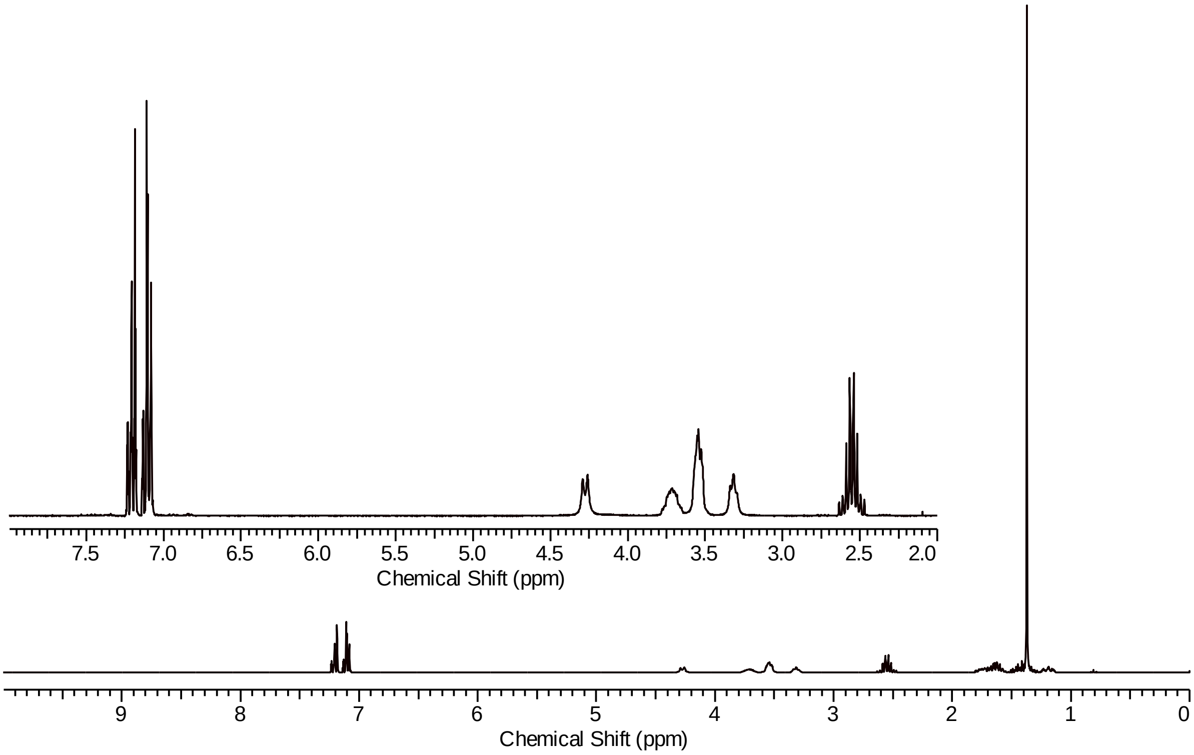

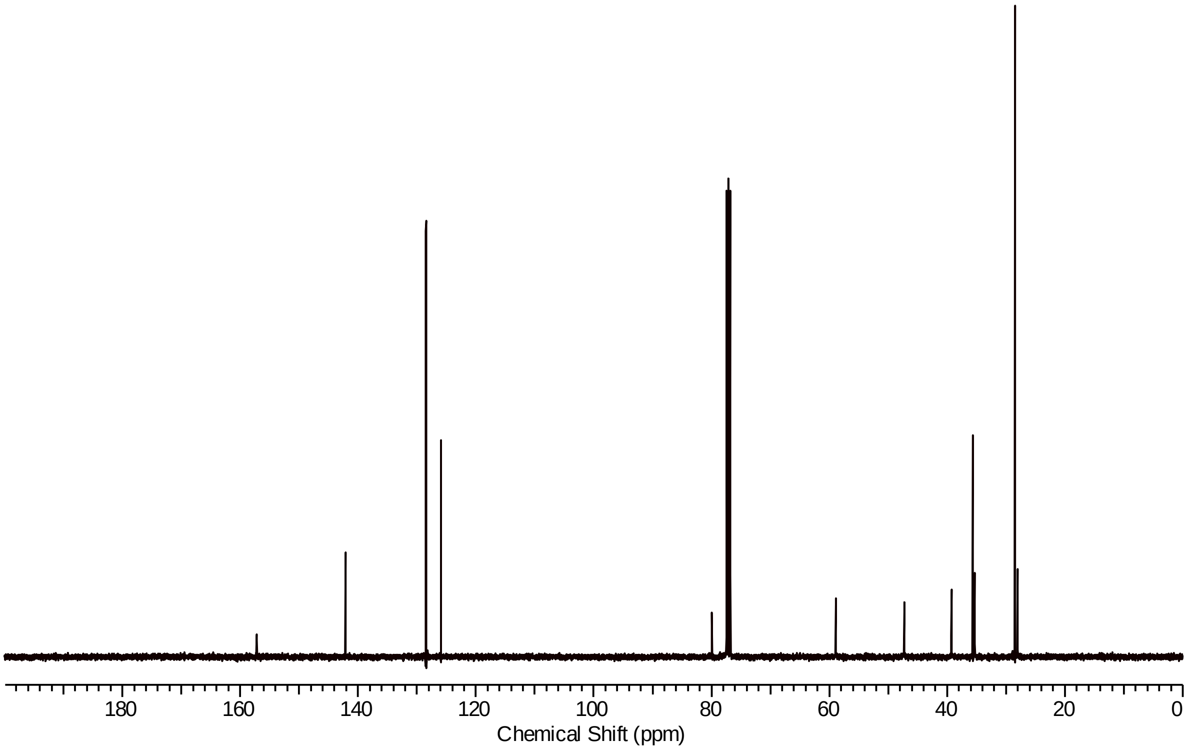

(*R*)-tert-butyl (1-oxo-6-phenylhexan-3-yl)carbamate (**5e**)

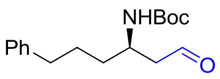

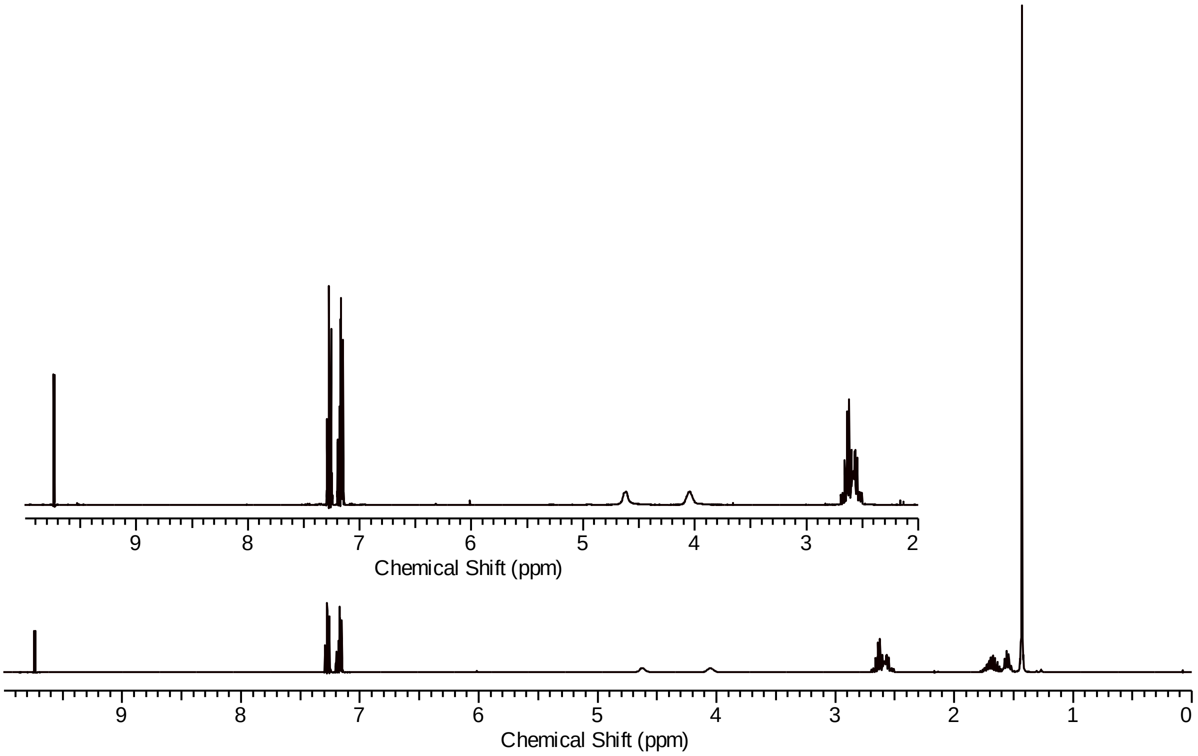

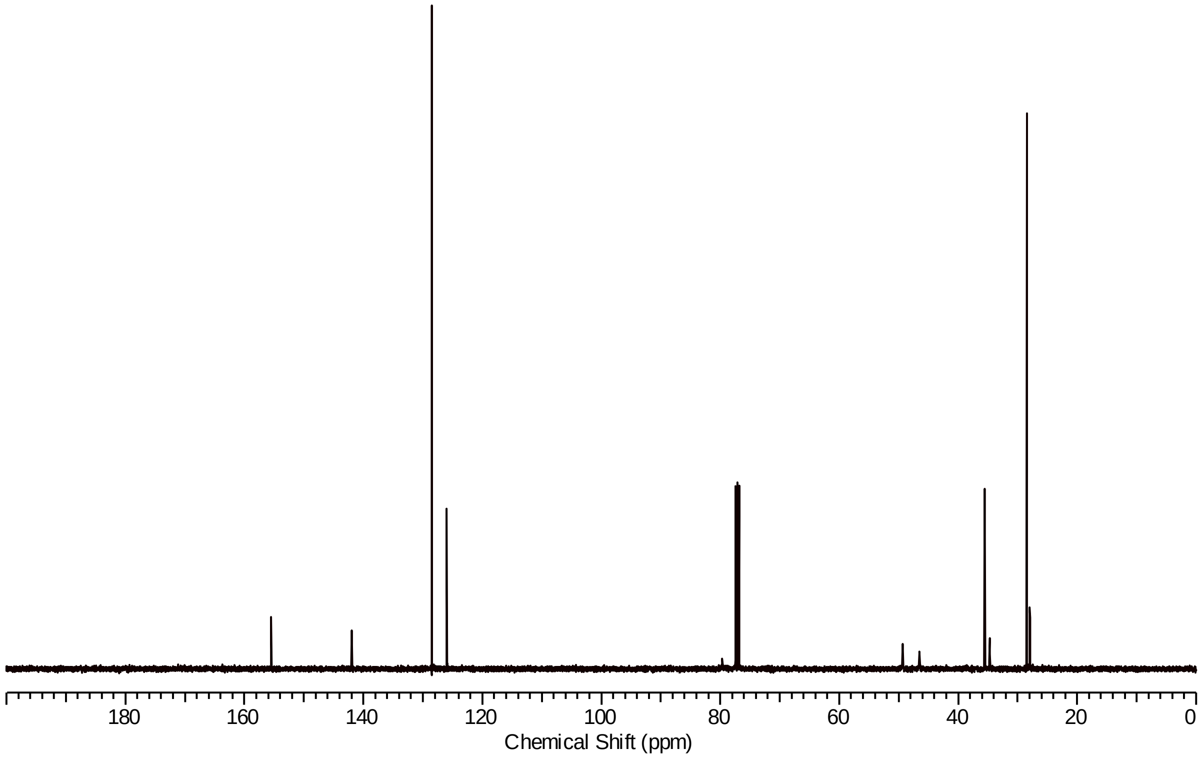

(*R*)-3-((*tert*-butoxycarbonyl)amino)-6-phenylhexanoic acid (**5f**)

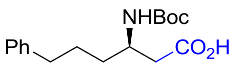

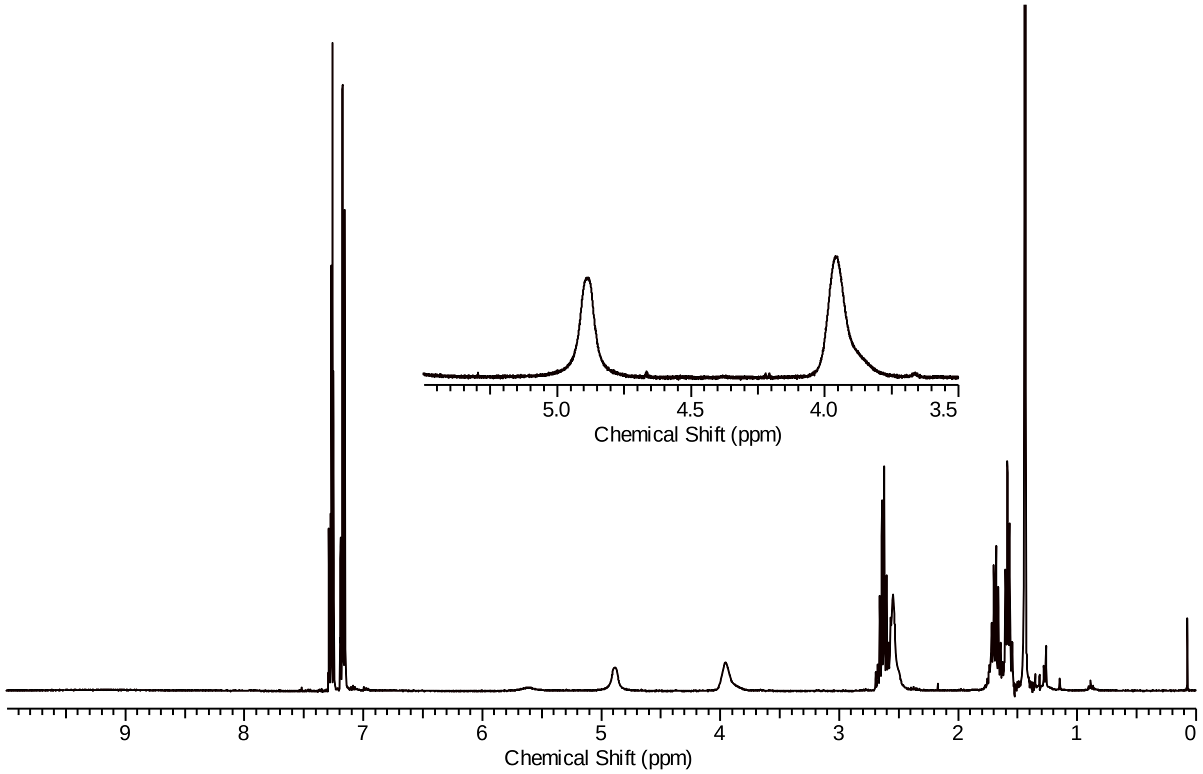

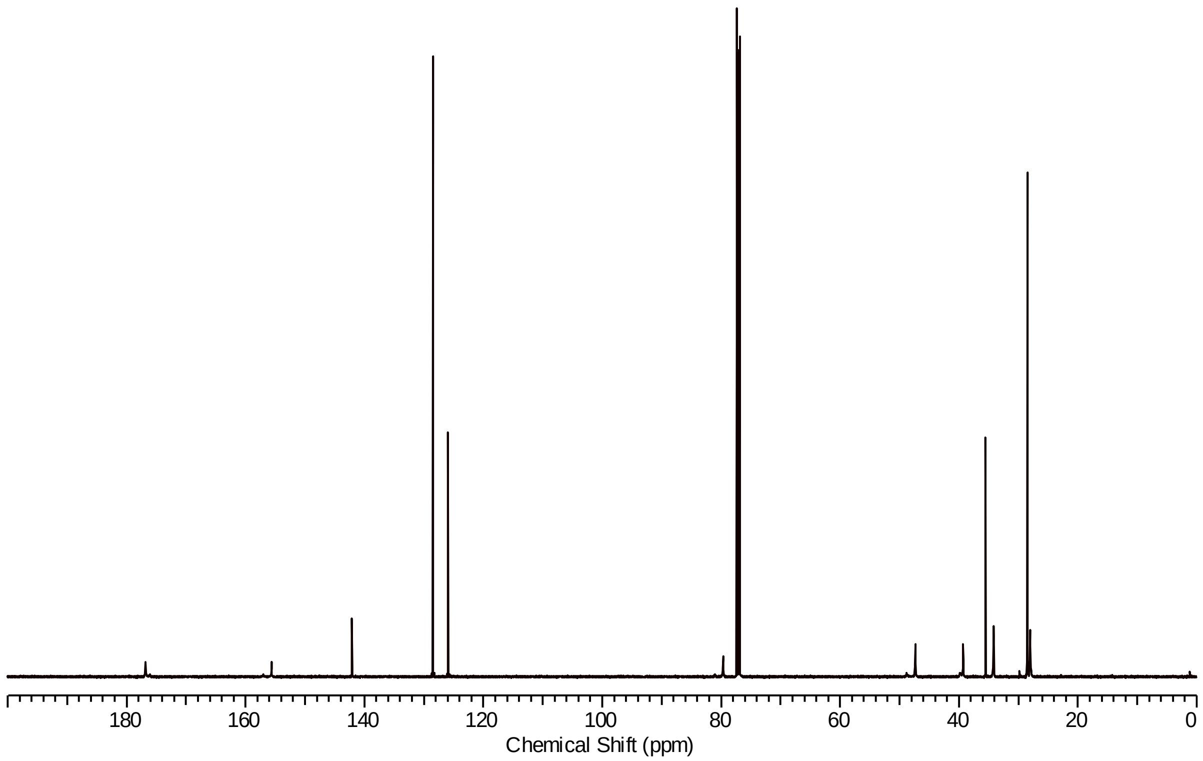

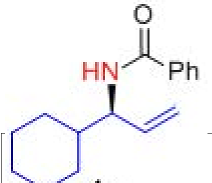

1a

Albert-Ludwigs-Universität Freiburg

Institut für Organische Chemie und Biochemie

kx-8016c ee {Data Description}

E:\EZDatenOC\KunXu\Method\OD-3, Hept_IPA, 95_5, 22°C, 200-400 nm, 1 ml.met

Vial: 182 Injection Volume (µl): 2
Run Time: 07.03.2014 14:02:25 Analysis Time: 02.11.2015 12:17:55

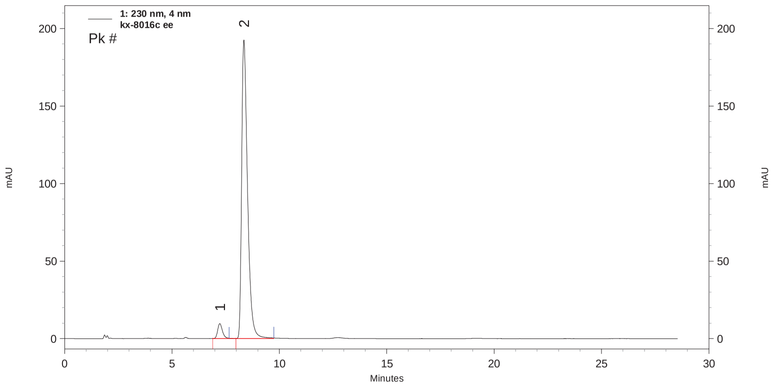

1: 230 nm, 4 nm Results

Pk #	Retention Time	Area Percent	Lambda Max
1	7,227	3,939	204
2	8,347	96,061	205

2: 250 nm, 4 nm Results

Pk #	Retention Time	Area Percent	Lambda Max
1	7,227	4,022	204
2	8,347	95,978	205

3: 210 nm, 4 nm Results

Pk #	Retention Time	Area Percent	Lambda Max
1	2,000	1,431	204
2	7,227	3,447	204
3	8,347	95,122	205

Spectrum Max Plot Results

Pk #	Retention Time	Area Percent	Lambda Max
1	2,000	1,972	204
2	7,227	3,207	204
3	8,347	93,701	205
4	12,653	1,119	201

DAD-225 nm Results

Pk #	Retention Time	Area Percent	Lambda Max
1	1,860	0,733	204
2	7,227	3,930	204
3	8,347	94,559	205
4	12,720	0,778	203

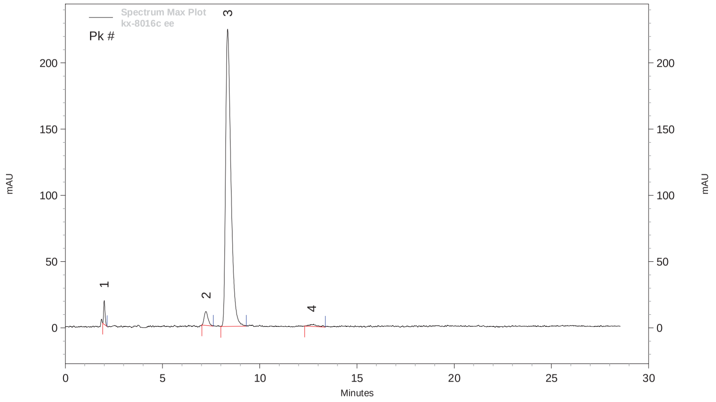

Albert-Ludwigs-Universität Freiburg

Institut für Organische Chemie und Biochemie

kx-8035 rac {Data Description}

E:\EZDatenOC\KunXu\Method\OD-3, Hept_IPA, 95_5, 22°C, 200-400 nm, 1 ml.met

Vial: 181 Injection Volume (µl): 2
Run Time: 05.03.2014 13:08:55 Analysis Time: 02.11.2015 12:15:49

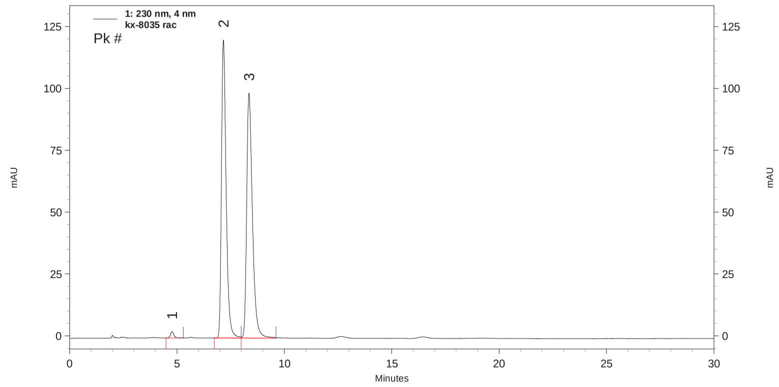

1: 230 nm, 4 nm Results

Pk #	Retention Time	Area Percent	Lambda Max
1	4,767	0,746	205
2	7,160	49,421	204
3	8,353	49,833	204

2: 250 nm, 4 nm Results

Pk #	Retention Time	Area Percent	Lambda Max
1	4,773	0,965	205
2	7,160	49,486	204
3	8,353	49,549	204

3: 210 nm, 4 nm Results

Pk #	Retention Time	Area Percent	Lambda Max
1	7,160	49,986	204
2	8,353	50,014	204

Spectrum Max Plot Results

Pk #	Retention Time	Area Percent	Lambda Max
1	2,007	1,983	204
2	4,773	1,631	205
3	7,160	47,169	204
4	8,353	47,537	204
5	24,960	1,679	203

DAD-225 nm Results

Pk #	Retention Time	Area Percent	Lambda Max
1	7,160	48,831	204
2	8,353	49,381	204
3	12,633	0,741	203
4	16,440	0,567	203
5	19,187	0,480	203

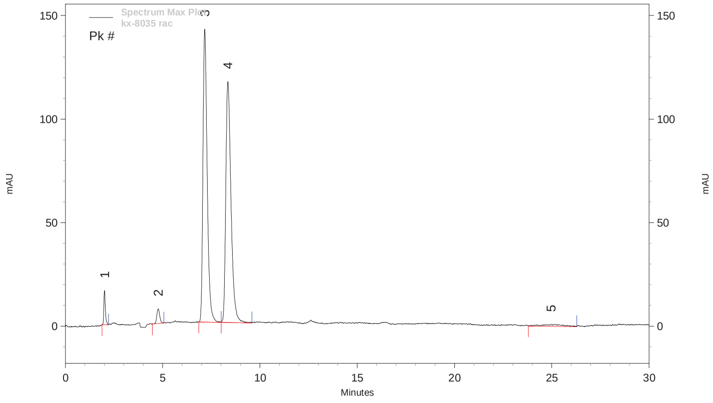

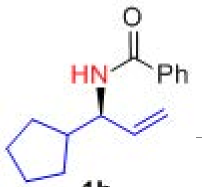

1b

Chromatogram :AD-H; Hep_IPA 98_2; 1,0ml; 230nm

Method : AD-H; Hep_IPA 98_2; 1,0ml; 230nm
User : TiBu
21.11.2014 14:20:28
TiBu
VK68_21.11.2014 14_19_22

Acquired : 21.11.2014 14:20:28
Processed : 21.11.2014 15:23:26
Printed : 21.11.2014 15:23:41
ORGPC119
Project1
System 1 (Normal Phase)

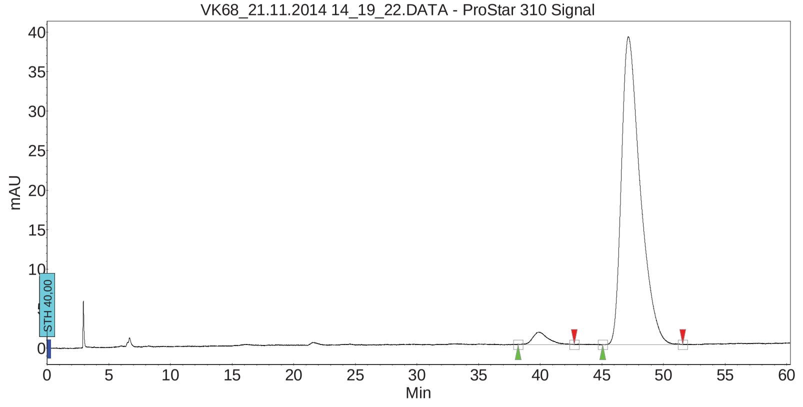

Peak results :

Index	Name	Time [Min]	Quantity [% Area]	Height [mAU]	Area [mAU.Min]	Area % [%]
1	UNKNOWN	39,90	3,17	1,6	2,3	3,172
2	UNKNOWN	47,15	96,83	39,0	68,8	96,828
Total			100,00	40,5	71,0	100,000

Page
1/$P
AGECOUNT

Chromatogram :AD-H; Hep_IPA 98_2; 1,0ml; 220nm

Method : AD-H; Hep_IPA 98_2; 1,0ml; 220nm
User : TiBu
20.11.2014 15:20:42
TiBu
VK05C-rac

Acquired : 20.11.2014 15:20:42
Processed : 20.11.2014 16:23:09
Printed : 20.11.2014 16:23:30
ORGPC119
Project1
System 1 (Normal Phase)

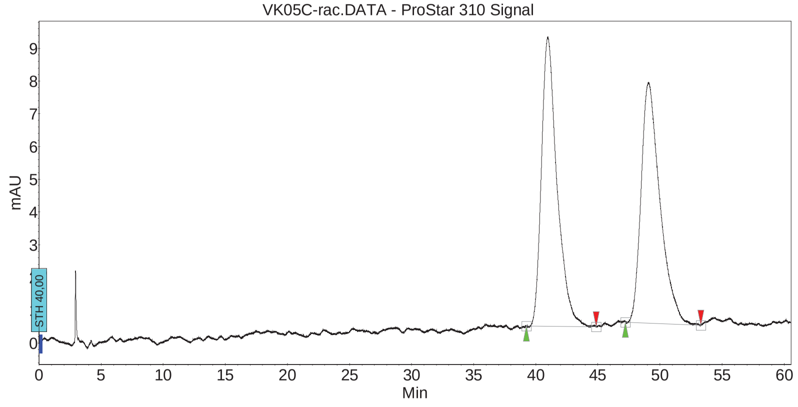

Peak results :

Index	Name	Time [Min]	Quantity [% Area]	Height [mAU]	Area [mAU.Min]	Area % [%]
1	UNKNOWN	40,97	49,82	8,8	12,9	49,825
2	UNKNOWN	49,09	50,18	7,3	12,9	50,175
Total			100,00	16,2	25,8	100,000

Page
1/$P
AGECOUNT

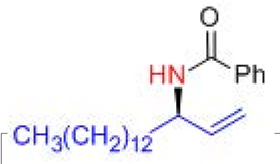

1c

Albert-Ludwigs-Universität Freiburg

Institut für Organische Chemie und Biochemie

vk-58 {Data Description}

E:\EZDatenOC\Doktorand 1\Method\OD3, Hept_IPA, 95_5, 22°C, 200-300 nm, 1ml.met

Vial: 193 Injection Volume (µl): 2
Run Time: 02.03.2015 11:50:27 Analysis Time: 02.11.2015 13:38:16

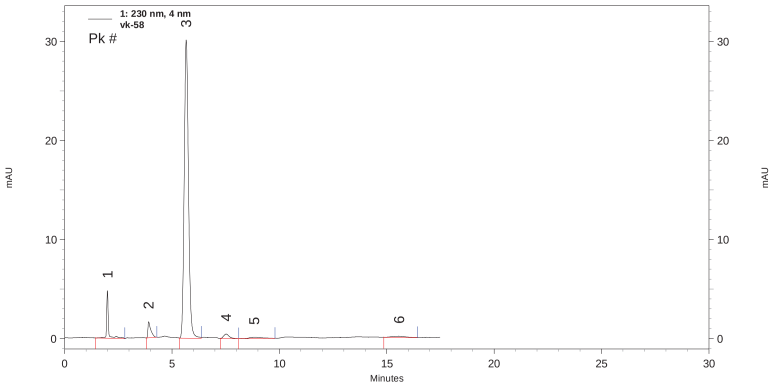

1: 230 nm, 4 nm Results

Pk #	Retention Time	Area Percent	Lambda Max
1	1,993	6,001	204
2	3,913	3,194	216
3	5,660	86,788	204
4	7,520	1,741	203
5	8,813	1,181	204
6	15,573	1,094	203

Spectrum Max Plot Results

Pk #	Retention Time	Area Percent	Lambda Max
1	1,993	19,039	204
2	2,400	0,265	204
3	2,667	0,469	203
4	3,920	2,281	216
5	5,660	59,574	204
6	7,000	1,230	203
7	7,520	1,660	203
8	8,887	3,377	204
9	9,513	0,270	203

10	11,160	4,115	203
11	11,533	0,515	203
12	13,493	3,040	203
13	15,593	4,164	203

DAD-225 nm Results

Pk #	Retention Time	Area Percent	Lambda Max
1	1,993	5,420	204
2	3,913	3,568	216
3	4,667	0,624	204
4	5,660	85,564	204
5	7,513	1,657	203
6	8,813	1,244	204
7	13,627	0,762	203
8	15,587	1,161	203

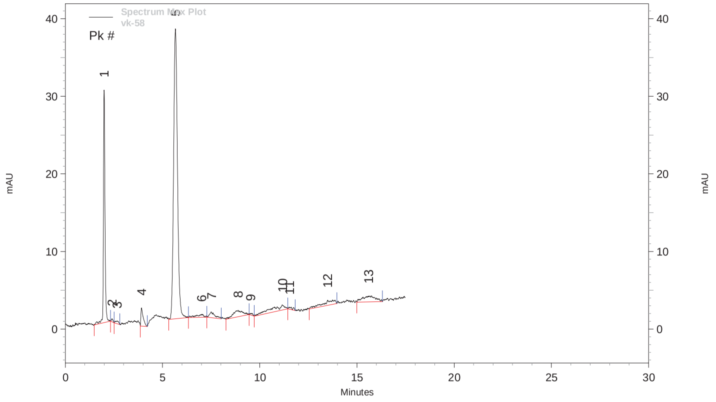

Albert-Ludwigs-Universität Freiburg

Institut für Organische Chemie und Biochemie

sh-8009-D rac {Data Description}

E:\EZDatenOC\Doktorand 1\Method\OD3, Hept_IPA, 95_5, 22°C, 200-300 nm, 1ml.met

Vial: 195 Injection Volume (µl): 2
Run Time: 15.10.2014 14:52:08 Analysis Time: 02.11.2015 13:36:10

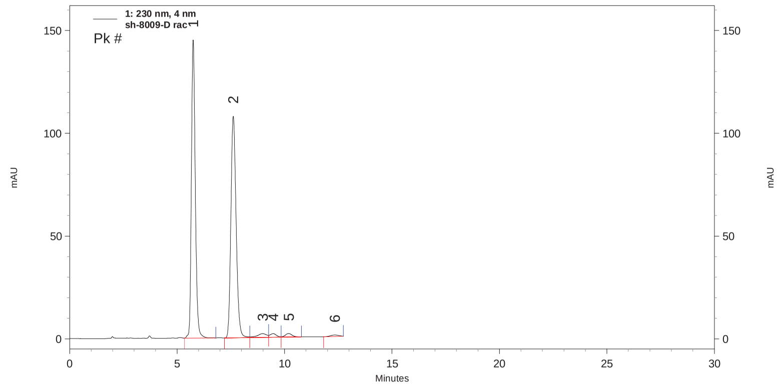

DAD-225 nm Results

Pk #	Retention Time	Area Percent	Lambda Max
1	1,993	0,250	205
2	5,747	48,049	204
3	7,613	47,957	204
4	9,467	2,178	204
5	10,187	1,026	204
6	12,340	0,540	204

Spectrum Max Plot Results

Pk #	Retention Time	Area Percent	Lambda Max
1	5,747	50,109	204
2	7,613	49,891	204

1: 230 nm, 4 nm Results

Pk #	Retention Time	Area Percent	Lambda Max
1	5,747	48,104	204
2	7,613	47,945	204
3	8,987	1,455	204
4	9,467	0,969	204
5	10,187	1,011	204
6	12,340	0,515	204

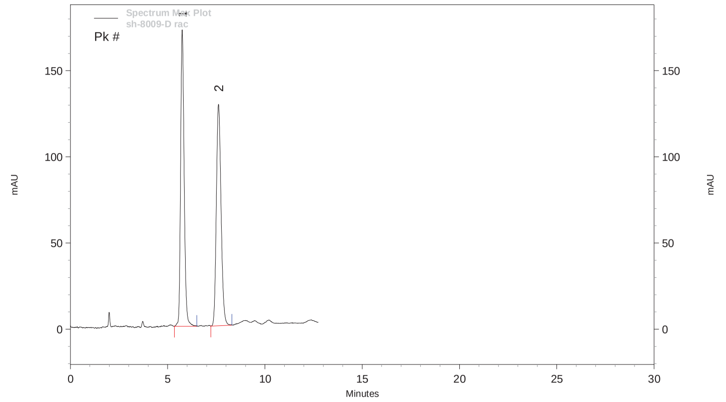

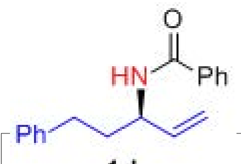

1d

Chromatogram :L-C2, Hep_IPA 95_5; 0,5ml; 210nm

Method : L-C2 Hep_IPA 95_5; 0,5ml; 210nm
User : Administrator
13.04.2015 17:38:43
Administrator
vk-9048 ee1

Acquired : 13.04.2015 17:38:43
Processed : 14.04.2015 09:20:25
Printed : 14.04.2015 09:26:02
ORGPC119
Project1
System 1 (Normal Phase)

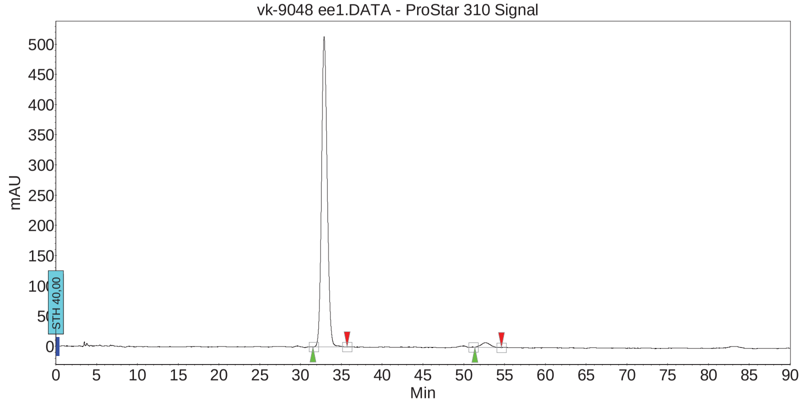

Peak results :

Index	Name	Time [Min]	Quantity [% Area]	Height [mAU]	Area [mAU.Min]	Area % [%]
1	UNKNOWN	32,89	97,65	513,6	411,9	97,655
2	UNKNOWN	52,67	2,35	7,4	9,9	2,345
Total			100,00	521,1	421,7	100,000

Page
1/$P
AGECOUNT

Chromatogram :L-C2, Hep_IPA 95_5; 0,5ml; 210nm

Method : L-C2 Hep_IPA 95_5; 0,5ml; 210nm
User : Administrator
13.04.2015 16:07:34
Administrator
VK-9040 rac1

Acquired : 13.04.2015 16:07:34
Processed : 14.04.2015 09:19:13
Printed : 14.04.2015 09:19:23
ORGPC119
Project1
System 1 (Normal Phase)

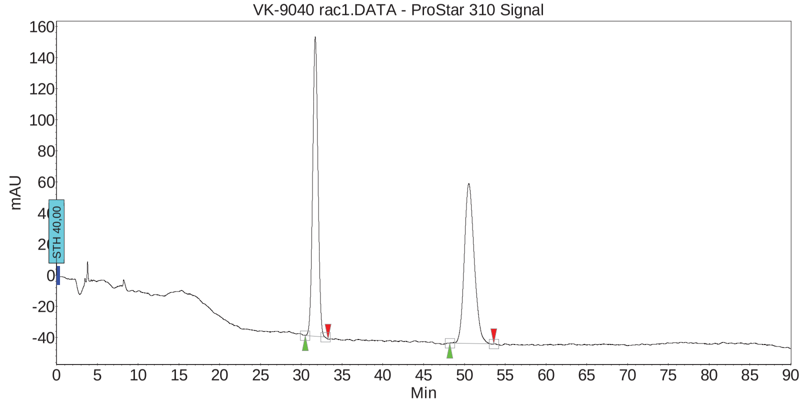

Peak results :

Index	Name	Time [Min]	Quantity [% Area]	Height [mAU]	Area [mAU.Min]	Area % [%]
1	UNKNOWN	31,72	50,00	192,6	144,9	50,001
2	UNKNOWN	50,54	50,00	102,9	144,9	49,999
Total			100,00	295,5	289,9	100,000

Page
1/$P
AGECOUNT

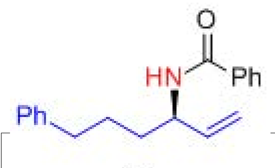

1e

Chromatogram :L-C2, Hep_IPA 98_2; 1ml; 210nm

Method : L-C2 Hep_IPA 98_2 1ml 210nm
User : Administrator
13.04.2015 22:12:17
Administrator
vk-9045 ee1

Acquired : 13.04.2015 22:12:17
Processed : 14.04.2015 09:31:04
Printed : 14.04.2015 09:31:12
ORGPC119
Project1
System 1 (Normal Phase)

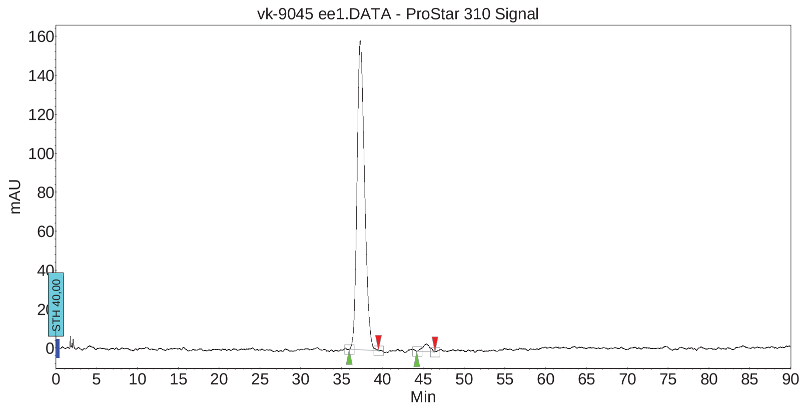

Peak results :

Index	Name	Time [Min]	Quantity [% Area]	Height [mAU]	Area [mAU.Min]	Area % [%]
1	UNKNOWN	37,28	97,62	158,7	170,3	97,616
2	UNKNOWN	45,39	2,38	4,0	4,2	2,384
Total			100,00	162,7	174,5	100,000

Page
1/$P
AGECOUNT

Chromatogram :L-C2, Hep_IPA 98_2; 1ml; 210nm

Method : L-C2 Hep_IPA 98_2 1ml 210nm
User : Administrator
13.04.2015 20:41:08
Administrator
vk-8054-c rac1

Acquired : 13.04.2015 20:41:08
Processed : 14.04.2015 09:28:08
Printed : 14.04.2015 09:28:18
ORGPC119
Project1
System 1 (Normal Phase)

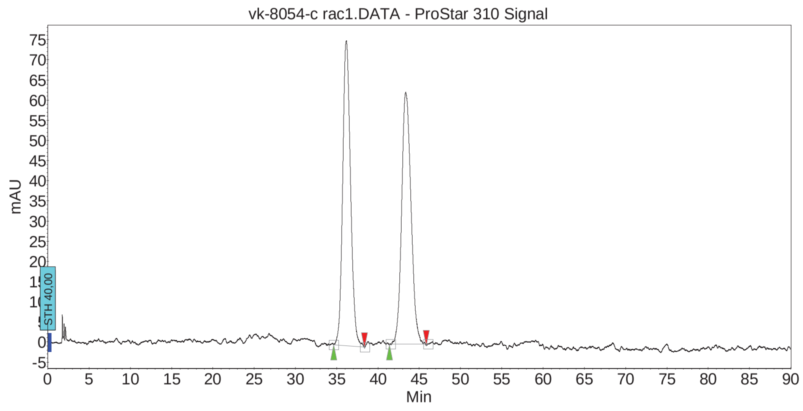

Peak results :

Index	Name	Time [Min]	Quantity [% Area]	Height [mAU]	Area [mAU.Min]	Area % [%]
1	UNKNOWN	36,15	50,25	75,6	83,3	50,254
2	UNKNOWN	43,36	49,75	62,5	82,5	49,746
Total			100,00	138,1	165,8	100,000

Page
1/$P
AGECOUNT

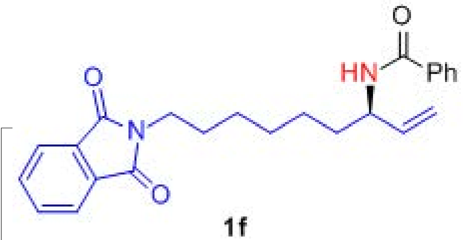

1f

sh-8075-ee Page 1 of 2

Albert-Ludwigs-Universität Freiburg

Institut für Organische Chemie und Biochemie

sh-8075-ee {Data Description}

E:\EZDatenOC\Doktorand 1\Method\OD-3, Hept_EtOH 85_15, 22°C, 1 ml.met

Vial: 196 Injection Volume (µl): 2
Run Time: 31.08.2015 12:19:53 Analysis Time: 02.11.2015 13:41:17

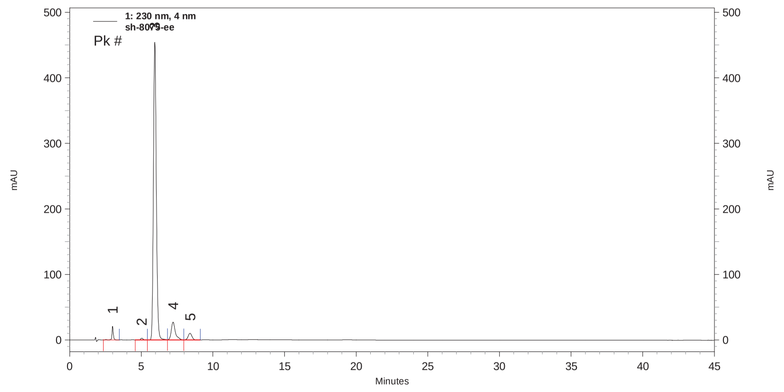

1: 230 nm, 4 nm Results

Pk #	Retention Time	Area Percent	Lambda Max
1	2,993	1,963	205
2	5,033	0,469	219
3	5,940	87,445	219
4	7,213	7,478	219
5	8,400	2,645	219

Spectrum Max Plot Results

Pk #	Retention Time	Area Percent	Lambda Max
1	1,813	0,334	202
2	2,000	0,384	204
3	2,993	3,317	205
4	4,307	0,079	220
5	5,033	0,519	219
6	5,940	86,032	219
7	7,213	7,038	219
8	8,400	2,297	219

sh-8075-ee Page 2 of 2

DAD-221 nm Results

Pk #	Retention Time	Area Percent	Lambda Max
1	2,993	1,527	205
2	5,033	0,471	219
3	5,940	85,072	219
4	7,213	7,358	219
5	8,400	2,638	219
6	11,367	0,757	218
7	12,767	0,868	218
8	13,700	0,483	219
9	19,353	0,479	219
10	20,693	0,346	220

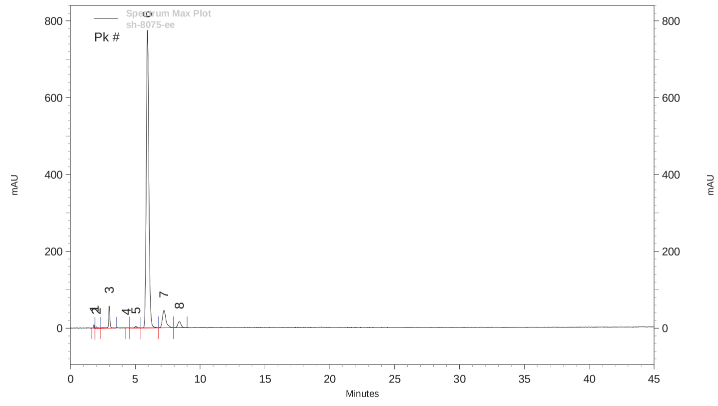

Albert-Ludwigs-Universität Freiburg

Institut für Organische Chemie und Biochemie

sh-8074-rac {Data Description}

E:\EZDatenOC\Doktorand 1\Method\OD-3, Hept_EtOH 85_15, 22°C, 1 ml.met

Vial: 195 Injection Volume (µl): 2
Run Time: 31.08.2015 11:39:31 Analysis Time: 02.11.2015 13:40:30

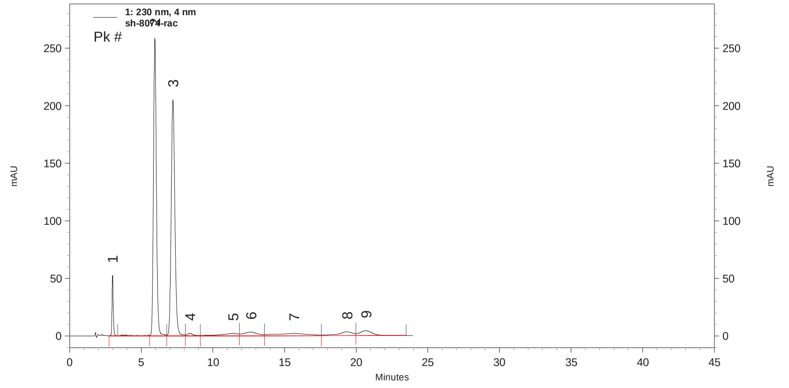

1: 230 nm, 4 nm Results

Pk #	Retention Time	Area Percent	Lambda Max
1	2,993	3,615	205
2	5,947	40,940	219
3	7,207	41,049	219
4	8,393	0,744	219
5	11,407	1,990	219
6	12,660	2,475	218
7	15,673	3,463	201
8	19,367	2,689	204
9	20,680	3,036	218

Spectrum Max Plot Results

Pk #	Retention Time	Area Percent	Lambda Max
1	1,813	0,781	204
2	2,993	6,514	205
3	5,947	40,950	219
4	7,207	41,011	219
5	11,413	1,501	219
6	12,667	2,049	218
7	15,673	2,008	201
8	19,367	2,464	204
9	20,667	2,723	218

DAD-221 nm Results

Pk #	Retention Time	Area Percent	Lambda Max
1	2,993	3,389	205
2	5,947	39,950	219
3	7,207	40,045	219
4	8,400	0,978	219
5	11,413	2,651	219
6	12,660	2,886	218
7	15,693	3,853	219
8	19,360	2,983	204
9	20,673	3,266	218

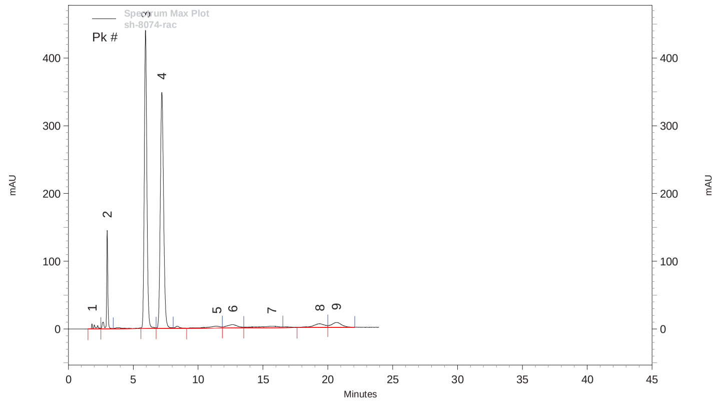

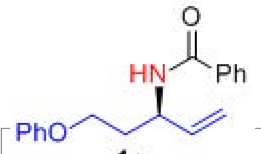

1g

Chromatogram :AD-H; Hep_IPA 90_10; 1,0ml; 230nm

Method : AD-H; Hep_IPA 90_10; 1,0ml; 230nm
User : TiBu
20.11.2014 08:48:52
TiBu
VK56

Acquired : 20.11.2014 08:48:52
Processed : 20.11.2014 09:23:42
Printed : 20.11.2014 09:23:54
ORGPC119
Project1
System 1 (Normal Phase)

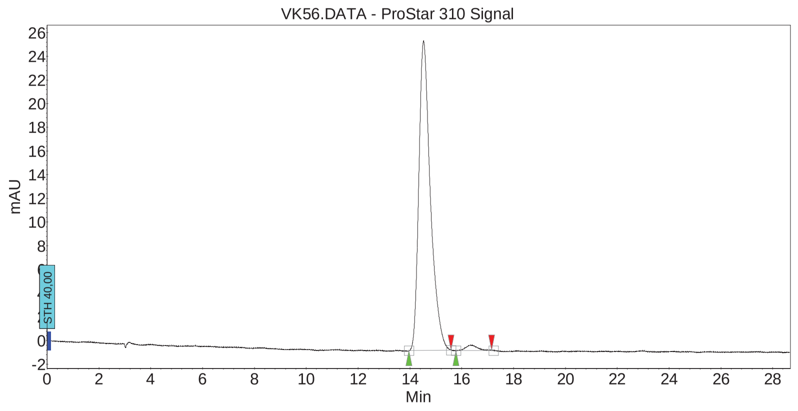

Peak results :

Index	Name	Time [Min]	Quantity [% Area]	Height [mAU]	Area [mAU.Min]	Area % [%]
1	UNKNOWN	14,53	98,25	26,1	13,5	98,250
2	UNKNOWN	16,34	1,75	0,5	0,2	1,750
Total			100,00	26,6	13,7	100,000

Page
1/$P
AGECOUNT

Chromatogram :AD-H; Hep_IPA 90_10; 1,0ml; 230nm

Method : AD-H; Hep_IPA 90_10; 1,0ml; 230nm
User : TiBu
20.11.2014 08:21:24
TiBu
VK09C-rac

Acquired : 20.11.2014 08:21:24
Processed : 20.11.2014 09:26:27
Printed : 20.11.2014 09:26:40
ORGPC119
Project1
System 1 (Normal Phase)

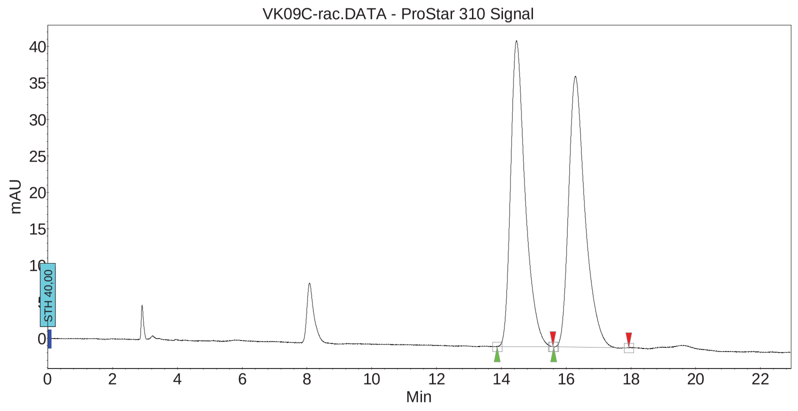

Peak results :

Index	Name	Time [Min]	Quantity [% Area]	Height [mAU]	Area [mAU.Min]	Area % [%]
1	UNKNOWN	14,47	50,09	41,9	21,8	50,089
2	UNKNOWN	16,29	49,91	37,1	21,7	49,911
Total			100,00	79,0	43,5	100,000

Page
1/$P
AGECOUNT

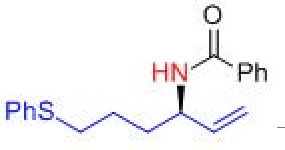

1h

Albert-Ludwigs-Universität Freiburg

Institut für Organische Chemie und Biochemie

vk-70 ee {Data Description}

E:\EZDatenOC\KunXu\Method\AD-3, Hept_EtOH, 85_15, 22°C, 1 ml.met

Vial: 192 Injection Volume (µl): 2
Run Time: 27.11.2014 13:26:44 Analysis Time: 02.11.2015 13:43:44

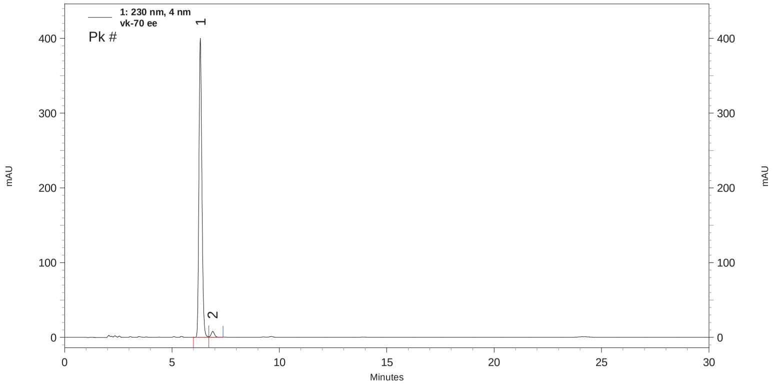

1: 230 nm, 4 nm Results

Pk #	Retention Time	Area Percent	Lambda Max
1	6,320	97,735	205
2	6,900	2,265	203

Spectrum Max Plot Results

Pk #	Retention Time	Area Percent	Lambda Max
1	2,053	1,899	204
2	6,320	95,773	205
3	6,900	2,328	203

DAD-255 nm Results

Pk #	Retention Time	Area Percent	Lambda Max
1	6,320	97,863	205
2	6,900	2,137	203

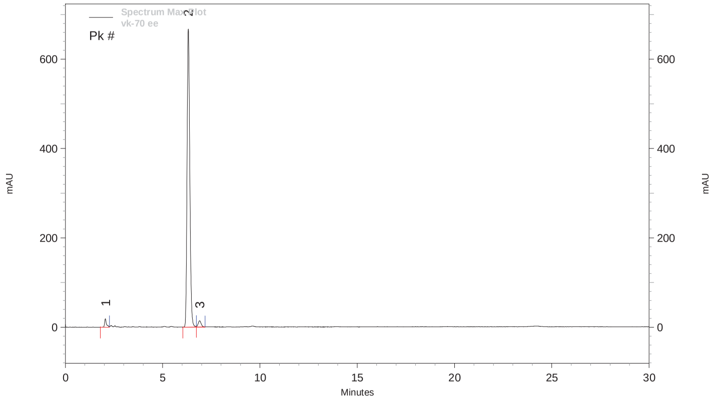

Albert-Ludwigs-Universität Freiburg

Institut für Organische Chemie und Biochemie

vk-10A rac {Data Description}

E:\EZDatenOC\KunXu\Method\AD-3, Hept_EtOH, 85_15, 22°C, 1 ml.met

Vial: 191 Injection Volume (µl): 2
Run Time: 27.11.2014 13:10:57 Analysis Time: 02.11.2015 13:42:46

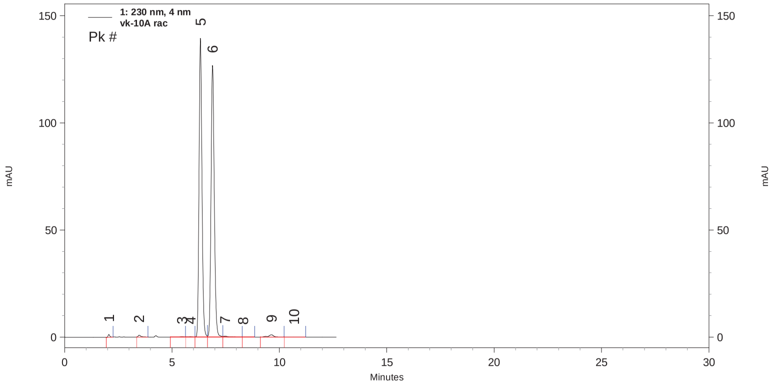

1: 230 nm, 4 nm Results

Pk #	Retention Time	Area Percent	Lambda Max
1	2,060	0,280	205
2	3,467	0,308	228
3	5,453	0,112	208
4	5,853	0,095	208
5	6,327	48,854	204
6	6,887	49,174	204
7	7,480	0,313	207
8	8,320	0,039	208
9	9,627	0,761	206
10	10,680	0,063	208

Spectrum Max Plot Results

Pk #	Retention Time	Area Percent	Lambda Max
1	2,060	1,060	205
2	3,467	0,180	228
3	4,247	0,096	228
4	6,320	48,774	204
5	6,887	48,980	204
6	9,620	0,694	206

7	10,607	0,120	208
8	11,880	0,095	208

DAD-255 nm Results

Pk #	Retention Time	Area Percent	Lambda Max
1	4,247	0,100	228
2	6,327	49,280	204
3	6,887	49,525	204
4	7,487	0,210	207
5	8,600	0,022	209
6	8,920	0,009	209
7	9,627	0,734	206
8	10,540	0,055	209
9	11,860	0,064	209

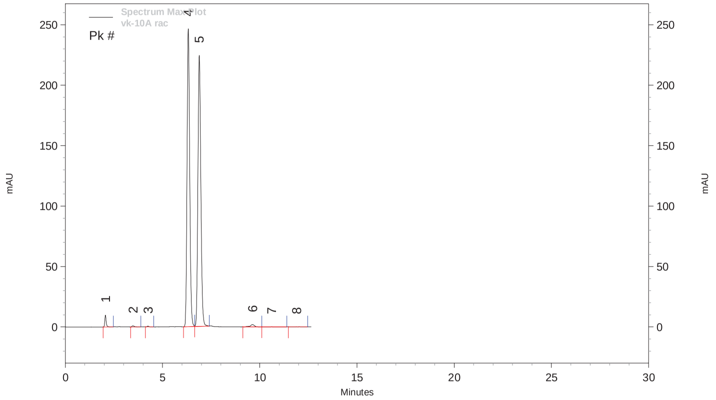

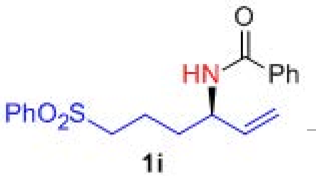

1i

Albert-Ludwigs-Universität Freiburg

Institut für Organische Chemie und Biochemie

vk-9073 ee {Data Description}

E:\EZDatenOC\Wang\Method\AD-3, Hept_IPA, 85_15, 22°C, 1ml.met

Vial: 192 Injection Volume (µl): 2
Run Time: 13.04.2015 12:45:15 Analysis Time: 02.11.2015 13:45:56

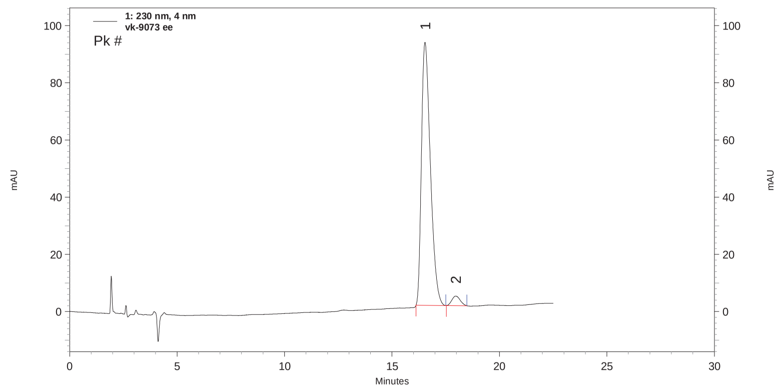

1: 230 nm, 4 nm Results

Pk #	Retention Time	Area Percent	Lambda Max
1	16,533	96,699	205
2	17,967	3,301	204

2: 210 nm, 4 nm Results

Pk #	Retention Time	Area Percent	Lambda Max
1	0,527	0,000	279
2	0,653	0,003	279
3	1,940	2,265	205
4	16,533	94,250	205
5	17,967	3,482	204

Spectrum Max Plot Results

Pk #	Retention Time	Area Percent	Lambda Max
1	16,533	96,340	205
2	17,953	3,660	204

DAD-219 nm Results

Pk #	Retention Time	Area Percent	Lambda Max
1	16,533	96,673	205
2	17,960	3,327	204

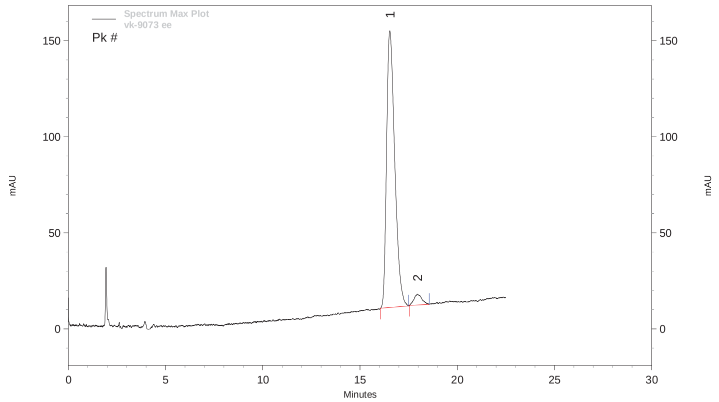

Albert-Ludwigs-Universität Freiburg

Institut für Organische Chemie und Biochemie

sh-8010-b {Data Description}

E:\EZDatenOC\Wang\Method\AD-3, Hept_IPA, 85_15, 22°C, 1ml.met

Vial: 192 Injection Volume (µl): 2
Run Time: 31.10.2014 11:38:17 Analysis Time: 02.11.2015 13:45:12

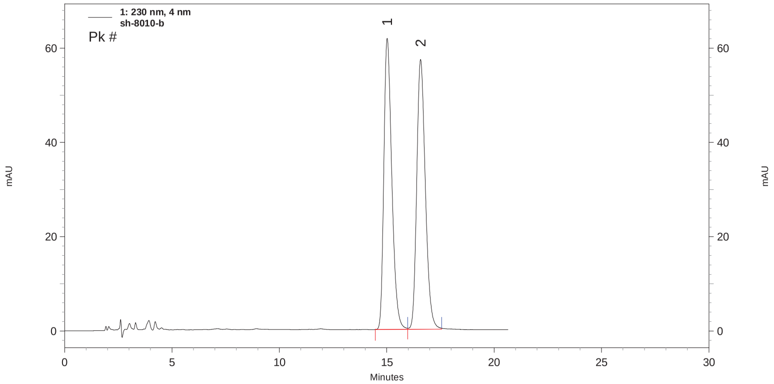

1: 230 nm, 4 nm Results

Pk #	Retention Time	Area Percent	Lambda Max
1	15,020	49,943	205
2	16,573	50,057	205

2: 210 nm, 4 nm Results

Pk #	Retention Time	Area Percent	Lambda Max
1	1,927	0,190	204
2	2,060	0,484	206
3	3,307	0,217	205
4	15,020	49,483	205
5	16,573	49,626	205

Spectrum Max Plot Results

Pk #	Retention Time	Area Percent	Lambda Max
1	2,060	1,097	206
2	15,020	49,344	205
3	16,573	49,560	205

DAD-219 nm Results

Pk #	Retention Time	Area Percent	Lambda Max
1	15,020	49,909	205
2	16,573	50,091	205

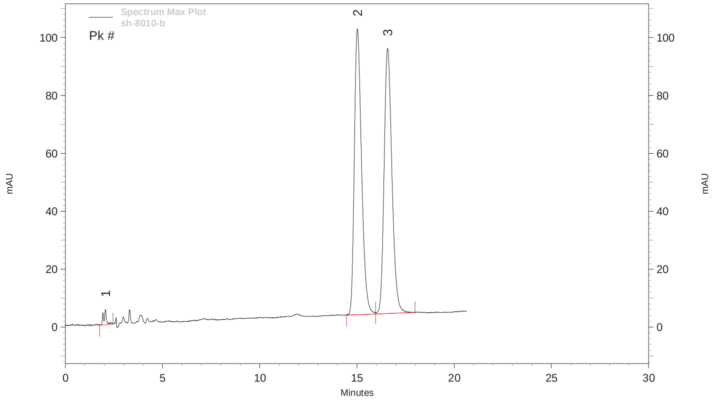

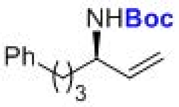

2a

Chromatogram :L-A2; Hep_EtOH, 98_2; 1,0ml; 210nm

Method : L-A2; Hep_EtOH, 98_2; 1,0ml; 210nm
User : SW
04.11.2015 10:55:21
SW
vk9063-ee

Acquired : 04.11.2015 10:55:21
Processed : 04.11.2015 11:15:12
Printed : 04.11.2015 11:15:22
ORGPC119
Project1
System 1 (Normal Phase)

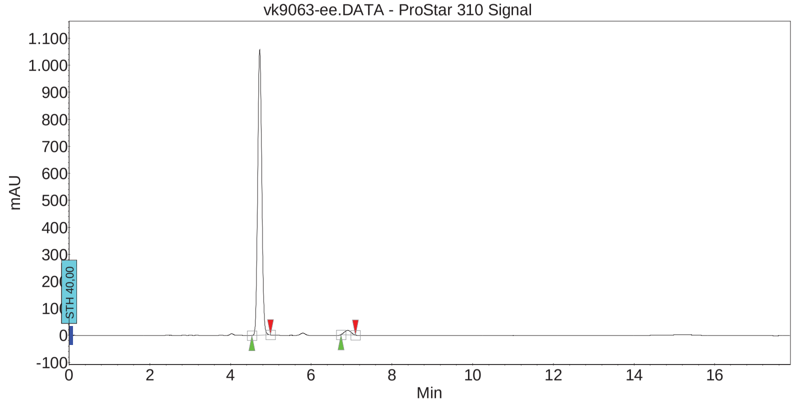

Peak results :

Index	Name	Time [Min]	Quantity [% Area]	Height [mAU]	Area [mAU.Min]	Area % [%]
2	UNKNOWN	4,72	97,51	1056,5	123,8	97,506
1	UNKNOWN	6,91	2,49	16,7	3,2	2,494
Total			100,00	1073,1	127,0	100,000

Page
1/$P
AGECOUNT

Chromatogram :L-A2; Hep_EtOH, 98_2; 1,0ml; 210nm

Method : L-A2; Hep_EtOH, 98_2; 1,0ml; 210nm
User : SW
04.11.2015 10:39:15
SW
sh8033-rac

Acquired : 04.11.2015 10:39:15
Processed : 04.11.2015 10:57:50
Printed : 04.11.2015 10:58:03
ORGPC119
Project1
System 1 (Normal Phase)

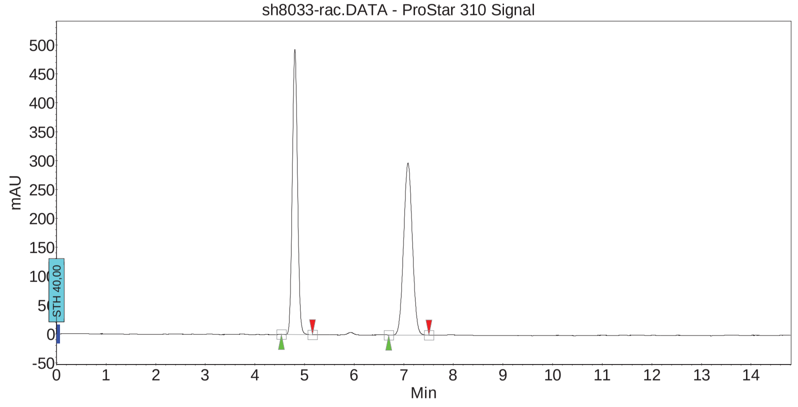

Peak results :

Index	Name	Time [Min]	Quantity [% Area]	Height [mAU]	Area [mAU.Min]	Area % [%]
1	UNKNOWN	4,80	49,61	492,9	59,8	49,608
2	UNKNOWN	7,08	50,39	297,7	60,7	50,392
Total			100,00	790,6	120,5	100,000

Page
1/$P
AGECOUNT

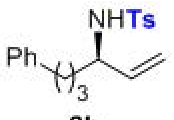

2b

Albert-Ludwigs-Universität Freiburg

Institut für Organische Chemie und Biochemie

sh-8040 ee {Data Description}

E:\EZDatenOC\Wang\Method\AD-3, Hept_EtOH, 90_10, 22°C, 200-300 nm, 1 ml a.met

Vial: 191 Injection Volume (µl): 2
Run Time: 20.08.2015 13:20:44 Analysis Time: 02.11.2015 14:03:18

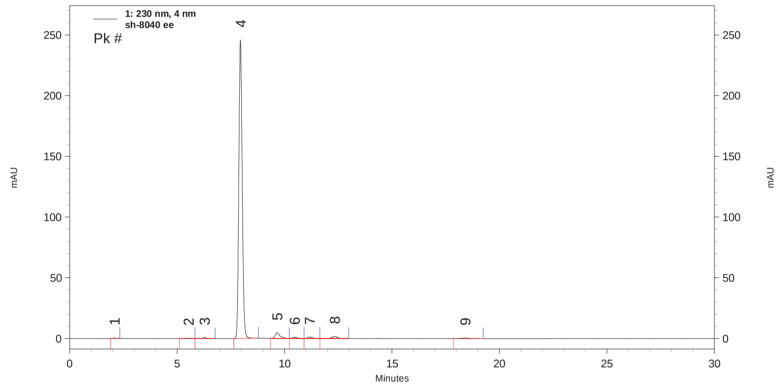

1: 230 nm, 4 nm Results

Pk #	Retention Time	Area Percent	Lambda Max
1	2,080	0,203	204
2	5,533	0,189	204
3	6,287	0,353	204
4	7,947	94,142	204
5	9,653	2,529	203
6	10,473	0,469	202
7	11,180	0,609	203
8	12,327	1,021	202
9	18,400	0,485	203

2: 250 nm, 4 nm Results

Pk #	Retention Time	Area Percent	Lambda Max
1	7,947	94,947	204
2	9,653	2,886	203
3	11,180	1,170	203
4	12,347	0,996	202

Spectrum Max Plot Results

Pk #	Retention Time	Area Percent	Lambda Max
1	2,073	0,544	204
2	7,947	86,509	204
3	9,653	3,281	203
4	10,460	0,687	202
5	11,193	1,351	203
6	12,340	2,384	202
7	13,080	0,977	202
8	14,100	0,535	202
9	14,553	1,032	202
10	15,887	0,572	202
11	16,380	0,695	201
12	18,407	1,433	203

DAD-210 nm Results

Pk #	Retention Time	Area Percent	Lambda Max
1	7,947	92,877	204
2	9,653	3,311	203
3	11,180	1,052	203
4	12,327	1,844	202
5	18,393	0,916	203

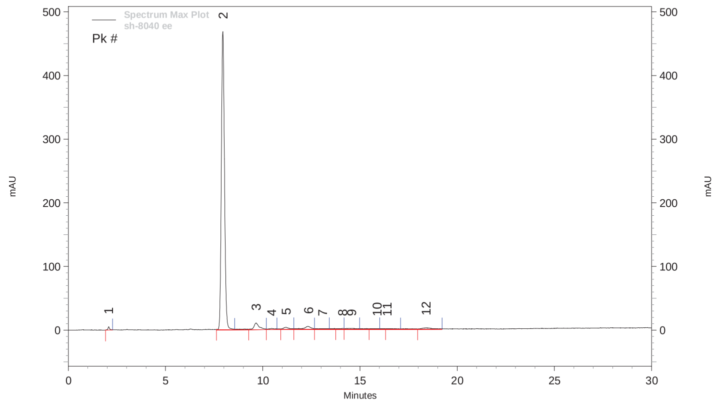

Albert-Ludwigs-Universität Freiburg

Institut für Organische Chemie und Biochemie

sh-8038-rac {Data Description}

E:\EZDatenOC\Wang\Method\AD-3, Hept_EtOH, 90_10, 22°C, 200-300 nm, 1 ml a.met

Vial: 190 Injection Volume (µl): 1
Run Time: 20.08.2015 11:41:37 Analysis Time: 02.11.2015 14:01:27

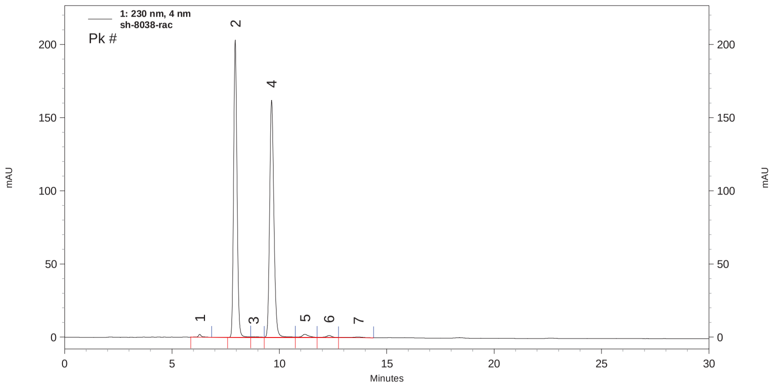

1: 230 nm, 4 nm Results

Pk #	Retention Time	Area Percent	Lambda Max
1	6,293	0,519	232
2	7,947	48,336	204
3	8,793	0,264	231
4	9,640	48,617	203
5	11,187	1,180	229
6	12,320	0,688	230
7	13,673	0,396	232

2: 250 nm, 4 nm Results

Pk #	Retention Time	Area Percent	Lambda Max
1	2,173	0,195	232
2	3,693	0,196	235
3	4,080	0,284	234
4	5,000	0,090	232
5	6,293	0,866	232
6	7,947	48,411	204
7	9,640	48,120	203
8	11,333	1,838	230

Spectrum Max Plot Results

Pk #	Retention Time	Area Percent	Lambda Max
1	2,073	0,076	201
2	6,287	0,217	232
3	7,947	47,786	204
4	9,060	0,332	232
5	9,640	48,261	203
6	11,187	1,694	229
7	12,333	0,897	230
8	13,627	0,597	232
9	29,607	0,141	279

DAD-210 nm Results

Pk #	Retention Time	Area Percent	Lambda Max
1	6,293	0,550	232
2	7,947	46,884	204
3	9,640	47,213	203
4	11,187	1,529	229
5	12,320	0,958	230
6	13,667	0,857	232
7	15,140	0,433	279
8	15,873	0,410	279
9	18,387	0,529	232
10	19,393	0,185	279
11	20,447	0,145	279
12	22,653	0,189	279
13	26,253	0,120	279

sh-8038-rac

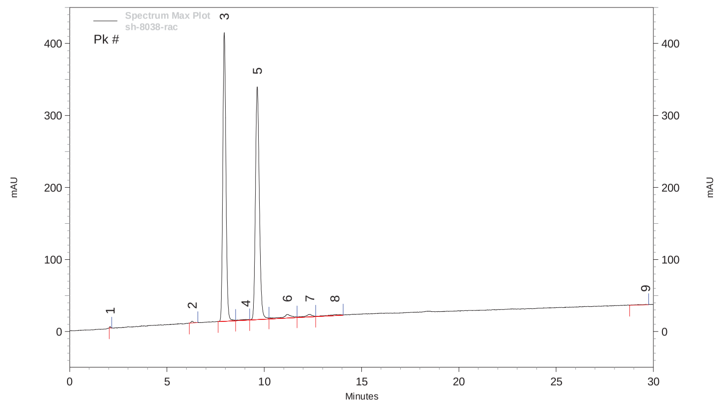
Spectrum Max Plot
sh-8038-rac
Pk #
mAU
Minutes
1
2
3
4
5
6
7
8
9
0
100
200
300
400
0
5
10
15
20
25
30

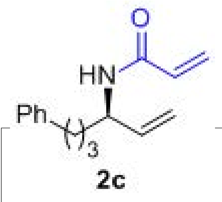

2c

Albert-Ludwigs-Universität Freiburg

Institut für Organische Chemie und Biochemie

sh-8034 ee {Data Description}

E:\EZDatenOC\KunXu\Method\L-A2, Hept_EtOH, 95_5, 22°C, 1 ml.met

Vial: 182 Injection Volume (µl): 2
Run Time: 14.07.2015 17:18:08 Analysis Time: 02.11.2015 14:12:24

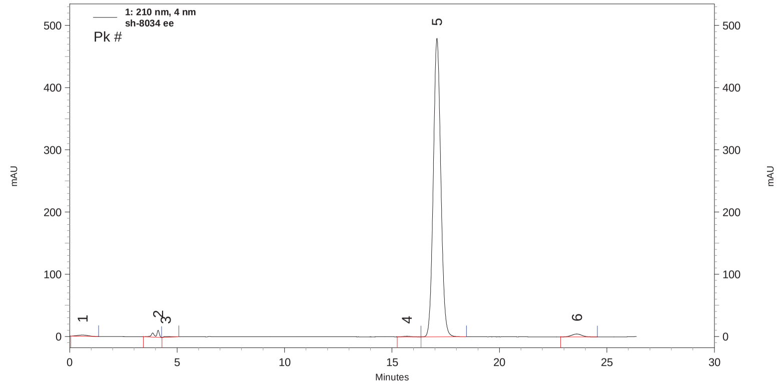

1: 210 nm, 4 nm Results

Pk #	Retention Time	Area Percent	Lambda Max
1	0,593	0,630	207
2	4,113	1,255	293
3	4,460	0,252	292
4	15,687	0,197	211
5	17,093	96,366	206
6	23,600	1,300	206

Spectrum Max Plot Results

Pk #	Retention Time	Area Percent	Lambda Max
1	0,580	0,738	207
2	3,867	0,546	202
3	4,133	1,755	293
4	6,440	0,213	293
5	17,093	95,723	206
6	23,613	1,026	206

DAD-229 nm Results

Pk #	Retention Time	Area Percent	Lambda Max
1	17,093	98,666	206
2	23,600	1,334	206

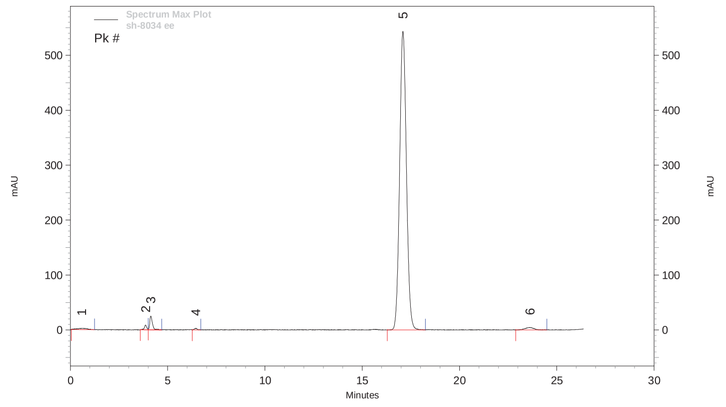

Albert-Ludwigs-Universität Freiburg

Institut für Organische Chemie und Biochemie

sh-8030-rac {Data Description}

E:\EZDatenOC\KunXu\Method\L-A2, Hept_EtOH, 95_5, 22°C, 1 ml.met

Vial: 181 Injection Volume (µl): 2
Run Time: 14.07.2015 16:47:54 Analysis Time: 02.11.2015 14:11:32

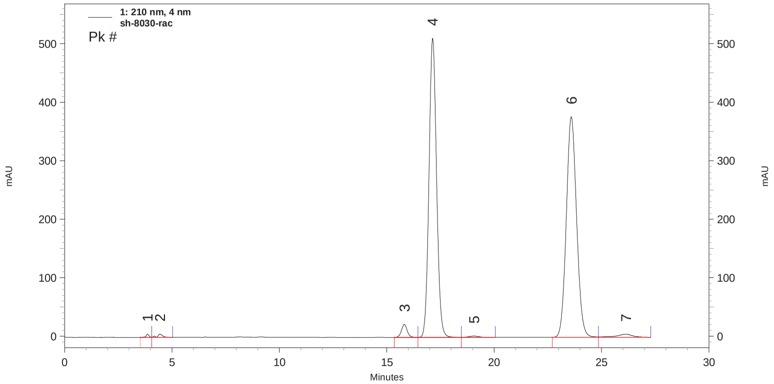

1: 210 nm, 4 nm Results

Pk #	Retention Time	Area Percent	Lambda Max
1	3,860	0,209	204
2	4,440	0,360	208
3	15,820	1,607	206
4	17,133	48,226	206
5	19,060	0,283	215
6	23,593	48,286	206
7	26,133	1,029	260

Spectrum Max Plot Results

Pk #	Retention Time	Area Percent	Lambda Max
1	3,860	0,110	204
2	4,193	0,054	227
3	4,440	0,134	208
4	5,127	0,008	255
5	6,480	0,060	293
6	8,107	0,002	292
7	15,820	1,243	206
8	17,140	48,441	206
9	19,073	0,016	215

10	23,593	48,567	206
11	25,793	1,365	260

DAD-229 nm Results

Pk #	Retention Time	Area Percent	Lambda Max
1	15,827	0,940	206
2	17,140	48,923	206
3	23,593	48,801	206
4	25,867	1,337	260

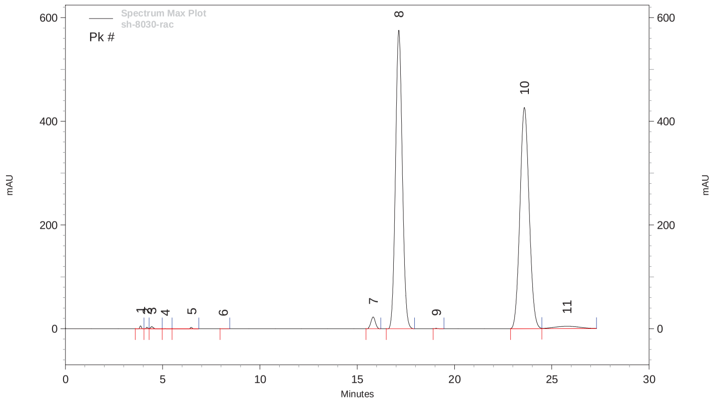

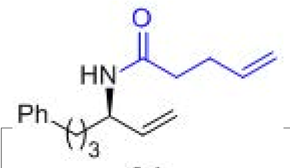

2d

vk-9061 ee Page 1 of 2

Albert-Ludwigs-Universität Freiburg

Institut für Organische Chemie und Biochemie

vk-9061 ee {Data Description}

E:\EZDatenOC\KunXu\Method\AD-3, Hept_EtOH, 97_3, 22°C, 1 ml.met

Vial: 182 Injection Volume (µl): 2
Run Time: 14.07.2015 12:57:57 Analysis Time: 02.11.2015 14:35:12

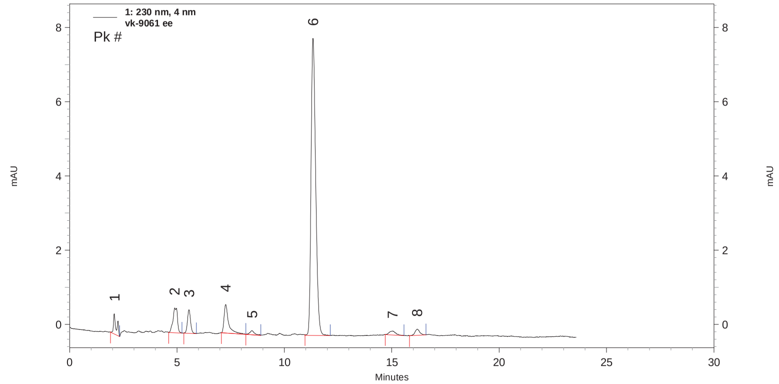

1: 230 nm, 4 nm Results

Pk #	Retention Time	Area Percent	Lambda Max
1	2,080	3,213	203
2	4,893	6,148	204
3	5,560	4,082	211
4	7,267	7,043	223
5	8,487	1,064	275
6	11,333	75,376	206
7	15,027	1,461	205
8	16,187	1,613	232

Spectrum Max Plot Results

Pk #	Retention Time	Area Percent	Lambda Max
1	4,973	0,458	261
2	9,247	0,290	204
3	11,333	97,447	206
4	15,007	1,805	205

vk-9061 ee Page 2 of 2

DAD-210 nm Results

Pk #	Retention Time	Area Percent	Lambda Max
1	9,247	0,369	204
2	11,333	97,607	206
3	15,007	2,024	205

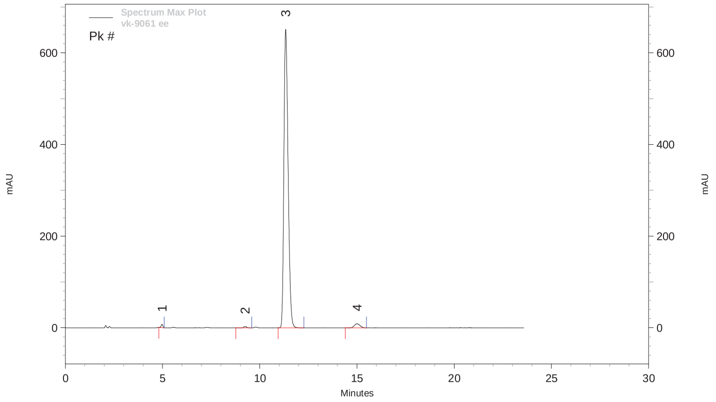

Albert-Ludwigs-Universität Freiburg

Institut für Organische Chemie und Biochemie

SH-8031-rac {Data Description}

E:\EZDatenOC\KunXu\Method\AD-3, Hept_EtOH, 97_3, 22°C, 1 ml.met

Vial: 181 Injection Volume (µl): 2
Run Time: 14.07.2015 12:28:23 Analysis Time: 02.11.2015 14:34:17

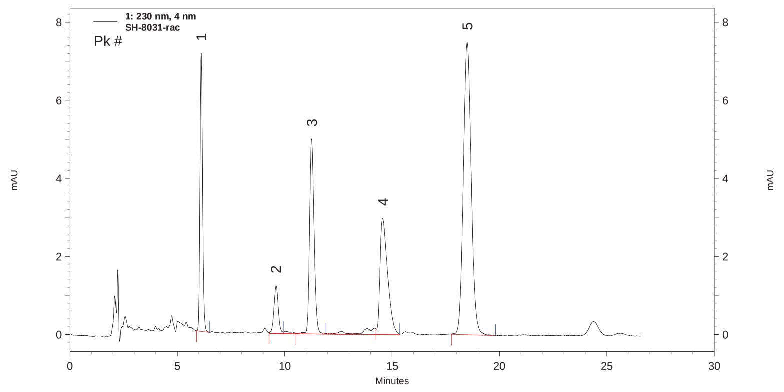

1: 230 nm, 4 nm Results

Pk #	Retention Time	Area Percent	Lambda Max
1	6,113	14,521	205
2	9,600	4,169	204
3	11,253	17,677	206
4	14,560	17,670	205
5	18,500	45,963	259

Spectrum Max Plot Results

Pk #	Retention Time	Area Percent	Lambda Max
1	6,113	2,000	205
2	9,600	0,819	204
3	11,253	45,706	206
4	14,560	46,638	205
5	18,500	4,838	259

DAD-210 nm Results

Pk #	Retention Time	Area Percent	Lambda Max

1	6,113	2,133	205
2	9,607	0,568	204
3	11,253	47,422	206
4	14,560	48,005	205
5	18,493	1,872	259

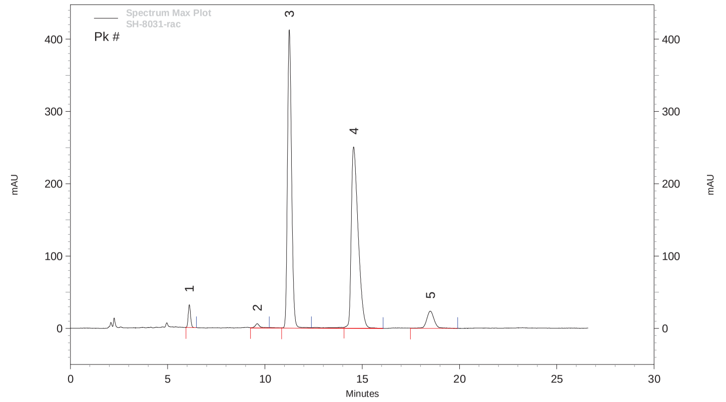

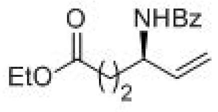

3aee

sh-8047-ee Page 1 of 2

Albert-Ludwigs-Universität Freiburg

Institut für Organische Chemie und Biochemie

sh-8047-ee {Data Description}

E:\EZDatenOC\Doktorand 1\Method\OD3, Hept_IPA, 90_10, 22°C, 1ml.met

Vial: 197 Injection Volume (µl): 2
Run Time: 31.08.2015 15:16:19 Analysis Time: 02.11.2015 15:00:36

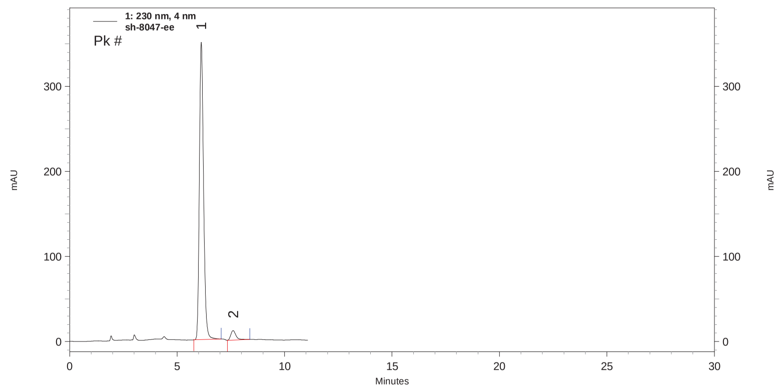

1: 230 nm, 4 nm Results

Pk #	Retention Time	Area Percent	Lambda Max
1	6,127	96,163	226
2	7,600	3,837	205

Spectrum Max Plot Results

Pk #	Retention Time	Area Percent	Lambda Max
1	6,127	97,057	226
2	7,613	2,943	204

DAD-226 nm Results

Pk #	Retention Time	Area Percent	Lambda Max
1	6,127	96,175	226
2	7,607	3,825	205

sh-8047-ee Page 2 of 2

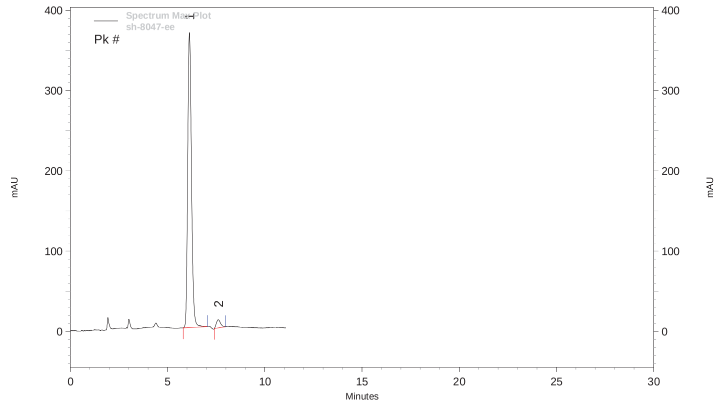

Albert-Ludwigs-Universität Freiburg
Institut für Organische Chemie und Biochemie

sh-8055-rac {Data Description}

E:\EZDatenOC\Doktorand 1\Method\OD3, Hept_IPA, 90_10, 22°C, 1ml.met

Vial: 193 Injection Volume (µl): 2
Run Time: 31.08.2015 14:50:26 Analysis Time: 02.11.2015 14:52:53

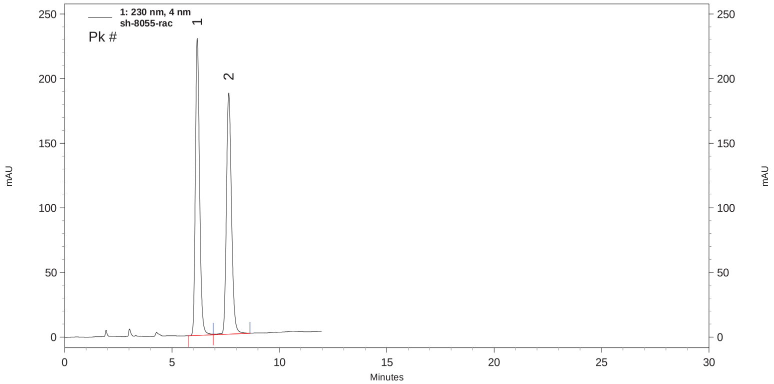

1: 230 nm, 4 nm Results

Pk #	Retention Time	Area Percent	Lambda Max
1	6,173	50,530	226
2	7,640	49,470	226

Spectrum Max Plot Results

Pk #	Retention Time	Area Percent	Lambda Max
1	1,933	1,626	206
2	3,020	1,618	211
3	4,407	0,596	205
4	6,173	47,930	226
5	7,640	46,809	226
6	10,607	1,421	206

DAD-226 nm Results

Pk #	Retention Time	Area Percent	Lambda Max
1	6,173	50,555	226
2	7,640	49,445	226

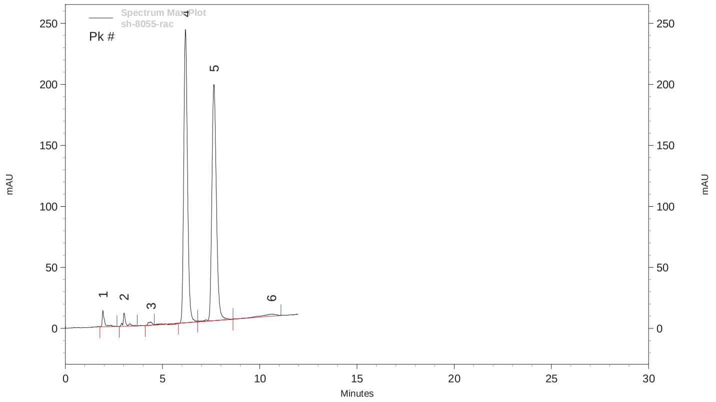

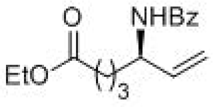

3bee

Chromatogram :L-C1; Hep_IPA 95_5; 1,0ml; 230nm

Method : L-C1 Hep_IPA 95_5 1,0ml 230nm
User : SW
10.09.2015 11:26:52
SW
sh8046-ee

Acquired : 10.09.2015 11:26:52
Processed : 10.09.2015 13:11:53
Printed : 10.09.2015 13:12:16
ORGPC119
Project1
System 1 (Normal Phase)

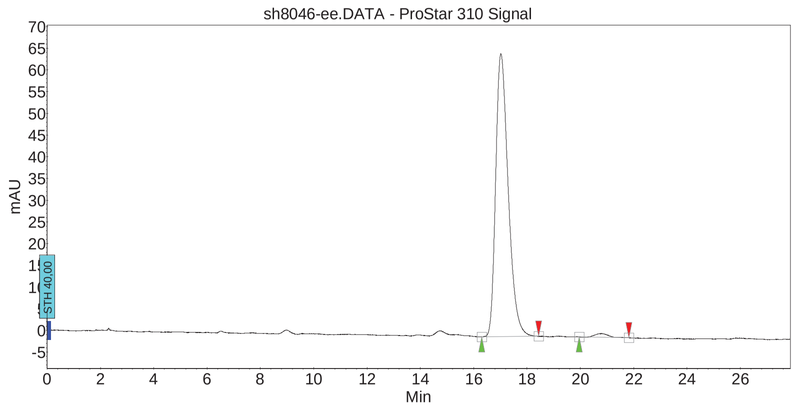

Peak results :

Index	Name	Time [Min]	Quantity [% Area]	Height [mAU]	Area [mAU.Min]	Area % [%]
1	UNKNOWN	17,02	98,72	65,1	35,4	98,718
2	UNKNOWN	20,81	1,28	0,8	0,5	1,282
Total			100,00	66,0	35,8	100,000

Page
1/$P
AGECOUNT

Chromatogram :L-C1; Hep_IPA 95_5; 1,0ml; 230nm

Method : L-C1 Hep_IPA 95_5 1,0ml 230nm
User : SW
10.09.2015 10:59:11
SW
sh8058-rac

Acquired : 10.09.2015 10:59:11
Processed : 10.09.2015 13:09:30
Printed : 10.09.2015 13:09:54
ORGPC119
Project1
System 1 (Normal Phase)

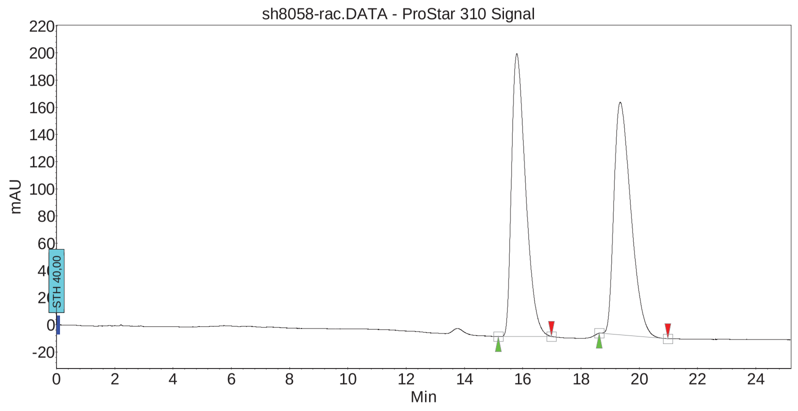

Peak results :

Index	Name	Time [Min]	Quantity [% Area]	Height [mAU]	Area [mAU.Min]	Area % [%]
1	UNKNOWN	15,80	50,13	208,1	116,2	50,134
2	UNKNOWN	19,35	49,87	171,3	115,6	49,866
Total			100,00	379,4	231,8	100,000

Page
1/$P
AGECOUNT

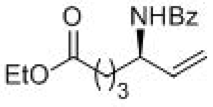

3cee

Chromatogram :L-C1; Hep_IPA 95_5; 1,0ml; 230nm

Method : L-C1 Hep_IPA 95_5 1,0ml 230nm
User : SW
10.09.2015 12:24:26
SW
sh8049-ee

Acquired : 10.09.2015 12:24:26
Processed : 10.09.2015 13:14:31
Printed : 10.09.2015 13:15:07
ORGPC119
Project1
System 1 (Normal Phase)

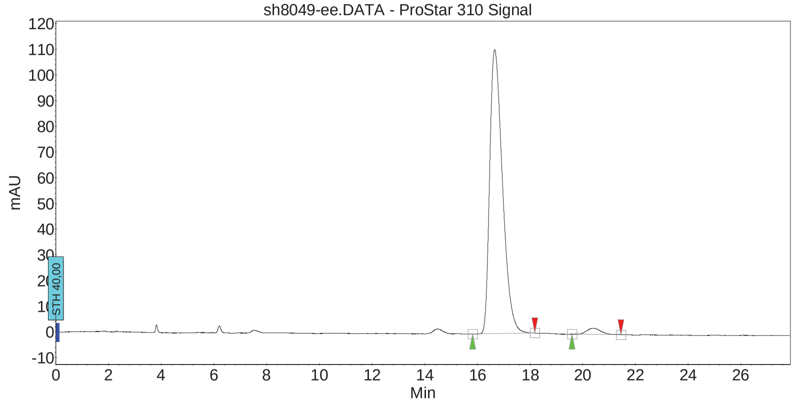

Peak results :

Index	Name	Time [Min]	Quantity [% Area]	Height [mAU]	Area [mAU.Min]	Area % [%]
1	UNKNOWN	16,66	97,64	110,5	62,0	97,645
2	UNKNOWN	20,39	2,36	2,4	1,5	2,355
Total			100,00	112,9	63,5	100,000

Page
1/$P
AGECOUNT

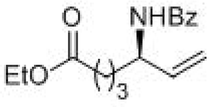

3dee

Chromatogram :L-C1; Hep_IPA 95_5; 1,0ml; 230nm

Method : L-C1 Hep_IPA 95_5 1,0ml 230nm
User : SW
10.09.2015 11:55:57
SW
sh8048-ee

Acquired : 10.09.2015 11:55:57
Processed : 10.09.2015 13:13:25
Printed : 10.09.2015 13:13:32
ORGPC119
Project1
System 1 (Normal Phase)

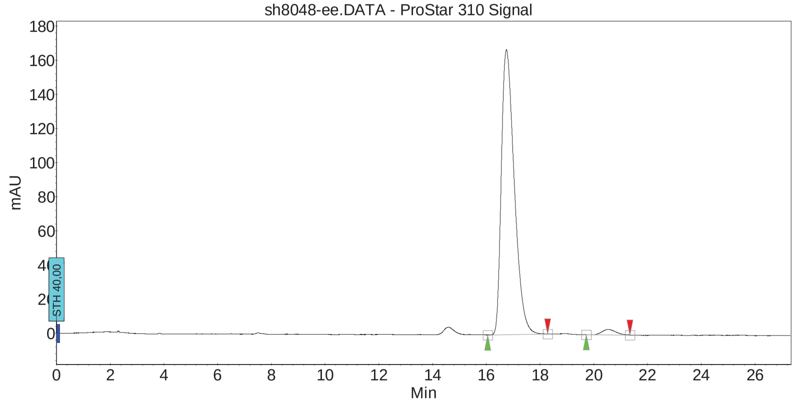

Peak results :

Index	Name	Time [Min]	Quantity [% Area]	Height [mAU]	Area [mAU.Min]	Area % [%]
1	UNKNOWN	16,75	98,01	166,9	93,2	98,009
2	UNKNOWN	20,56	1,99	3,2	1,9	1,991
Total			100,00	170,1	95,1	100,000

Page
1/$P
AGECOUNT

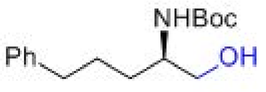

5a

Chromatogram :LC_1 Hep_IPA 95_5; 1,0ml; 210nm

Method : LC_1 Hep_IPA 95_5 1,0ml 210nm
User : SW
24.09.2015 11:16:16
SW
sh8044-ee

Acquired : 24.09.2015 11:16:16
Processed : 24.09.2015 11:33:20
Printed : 24.09.2015 11:33:31
ORGPC119
Project1
System 1 (Normal Phase)

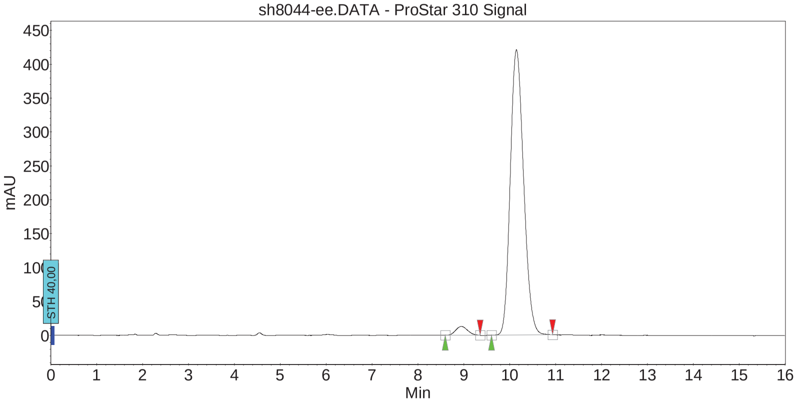

Peak results :

Index	Name	Time [Min]	Quantity [% Area]	Height [mAU]	Area [mAU.Min]	Area % [%]
2	UNKNOWN	8,95	2,71	13,1	3,9	2,710
1	UNKNOWN	10,15	97,29	420,8	141,8	97,290
Total			100,00	433,9	145,8	100,000

Page
1/$P
AGECOUNT

Chromatogram :LC_1 Hep_IPA 95_5; 1,0ml; 210nm

Method : LC_1 Hep_IPA 95_5 1,0ml 210nm
User : SW
24.09.2015 11:02:43
SW
sh8020B-rac

Acquired : 24.09.2015 11:02:43
Processed : 24.09.2015 11:16:26
Printed : 24.09.2015 11:16:39
ORGPC119
Project1
System 1 (Normal Phase)

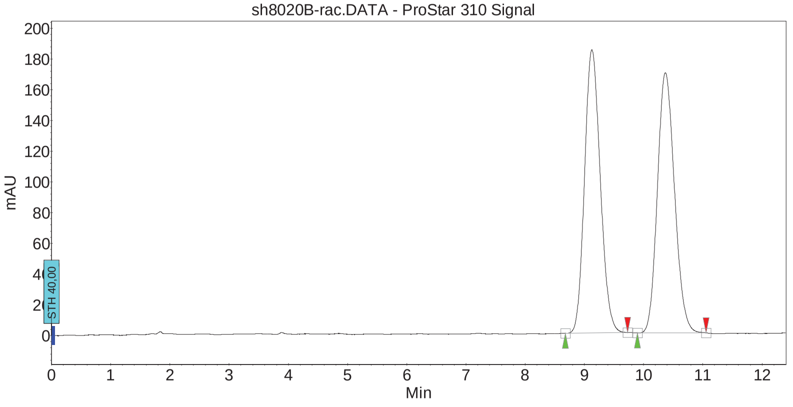

Peak results :

Index	Name	Time [Min]	Quantity [% Area]	Height [mAU]	Area [mAU.Min]	Area % [%]
1	UNKNOWN	9,13	49,97	184,3	56,7	49,967
2	UNKNOWN	10,37	50,03	169,3	56,8	50,033
Total			100,00	353,7	113,5	100,000

Page
1/$P
AGECOUNT

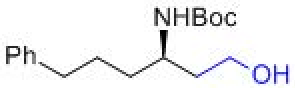

5d

Albert-Ludwigs-Universität Freiburg

Institut für Organische Chemie und Biochemie

sh-8042-D-ee {Data Description}

E:\EZDatenOC\Doktorand 1\Method\AD-3, Hept_EtOH, 90_10, 22°C, 1ml.met

Vial: 195 Injection Volume (µl): 2
Run Time: 18.09.2015 09:52:19 Analysis Time: 02.11.2015 15:02:33

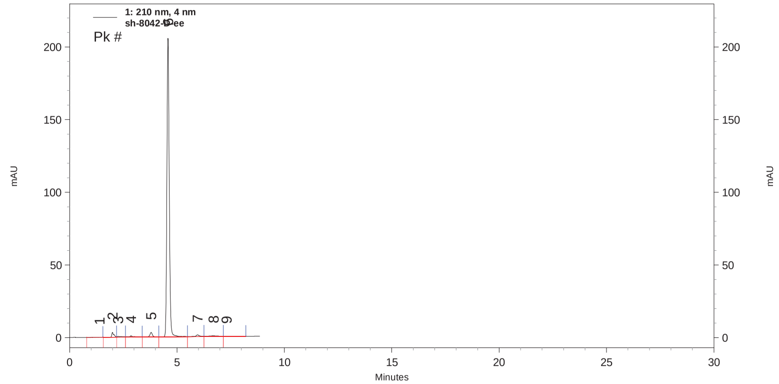

1: 210 nm, 4 nm Results

Pk #	Retention Time	Area Percent	Lambda Max
1	1,400	0,082	202
2	1,993	1,427	202
3	2,260	0,556	202
4	2,860	0,930	202
5	3,793	1,767	202
6	4,580	92,614	208
7	5,953	1,056	202
8	6,700	1,064	202
9	7,293	0,504	202

Spectrum Max Plot Results

Pk #	Retention Time	Area Percent	Lambda Max
1	2,000	2,150	202
2	3,793	1,137	202
3	4,580	96,187	208
4	5,960	0,525	202

DAD-214 nm Results

Pk #	Retention Time	Area Percent	Lambda Max
1	1,993	1,129	202
2	3,793	1,443	202
3	4,580	97,428	208

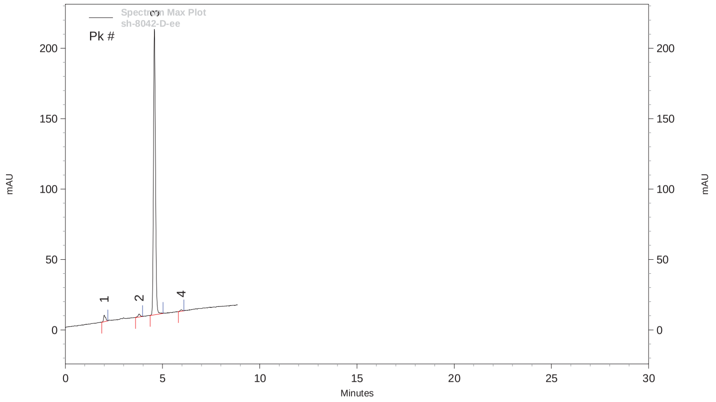

Albert-Ludwigs-Universität Freiburg
Institut für Organische Chemie und Biochemie

sh-8042-B-rac {Data Description}

E:\EZDatenOC\Doktorand 1\Method\AD-3, Hept_EtOH, 90_10, 22°C, 1ml.met

Vial: 192 Injection Volume (µl): 2
Run Time: 18.09.2015 09:40:51 Analysis Time: 02.11.2015 15:01:52

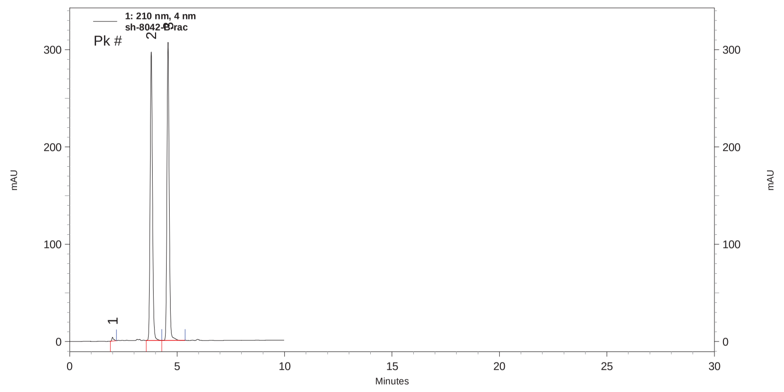

1: 210 nm, 4 nm Results

Pk #	Retention Time	Area Percent	Lambda Max
1	1,993	0,520	202
2	3,800	49,595	208
3	4,573	49,885	208

Spectrum Max Plot Results

Pk #	Retention Time	Area Percent	Lambda Max
1	2,000	1,033	202
2	3,800	50,040	208
3	4,573	48,927	208

DAD-214 nm Results

Pk #	Retention Time	Area Percent	Lambda Max
1	3,800	49,829	208
2	4,573	50,171	208

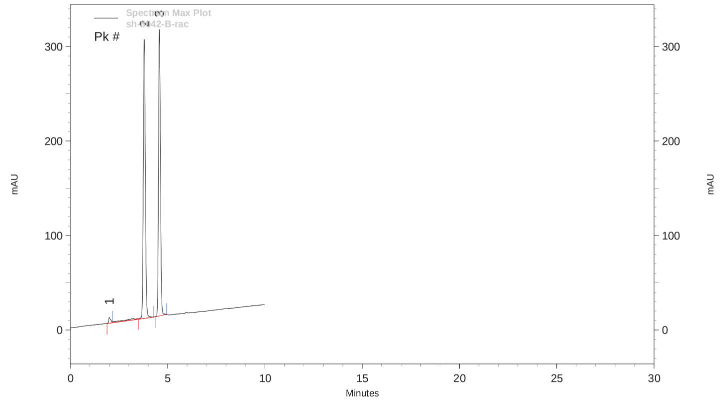

Supplement: Supplementary file 1 [file SC-007-C5SC04984A-s001.pdf]
